# Application of the Gross Motor Function Measure in children with conditions other than cerebral palsy: A systematic review

**DOI:** 10.1111/dmcn.16465

**Published:** 2025-08-14

**Authors:** Hirokazu Abe, Shuhei Higashi, Nobuaki Himuro

**Affiliations:** ^1^ Department of Health Care and Child Development Saitama Children's Medical Center Saitama Japan; ^2^ Department of Physical Therapy Kitakyushu Children's Rehabilitation Center Kitakyushu Japan; ^3^ Department of Public Health, School of Medicine Sapporo Medical University Sapporo Japan

## Abstract

**Aim:**

To investigate the application and evaluate the measurement properties of the Gross Motor Function Measure (GMFM) in children with conditions other than cerebral palsy (CP).

**Method:**

A systematic review was conducted using five electronic databases to identify studies that used the GMFM in children with conditions other than CP. Methodological quality and measurement properties were evaluated using established standards for assessing outcome measures.

**Results:**

We identified 210 studies across various paediatric conditions. Measurement property studies examined eight conditions: acquired brain injury, spinal muscular atrophy, Fukuyama congenital muscular dystrophy, Down syndrome, osteogenesis imperfecta, acute lymphoblastic leukaemia (ALL), leukodystrophy, and Pompe disease. Evidence quality was generally low to very low owing to small sample sizes and methodological limitations. Reliability showed sufficient ratings across most conditions. Content validity was examined only for ALL and demonstrated sufficient ratings. Responsiveness and construct validity showed variable results across conditions. Clinical application analysis revealed inadequate methodological reporting and widespread use without appropriate validation.

**Interpretation:**

GMFM validation for conditions other than CP remains insufficient despite widespread use. Content validity verification and enhanced methodological rigor are critically needed. Clinicians should interpret results cautiously until robust validation is established.

## Graphical abstract

This systematic review found that while the Gross Motor Function Measure (GMFM) is widely used across 208 studies involving various pediatric conditions beyond cerebral palsy, validation studies exist for only eight conditions with generally low‐quality evidence. Reliability showed sufficient ratings across conditions, but most studies had methodological limitations, highlighting the need for standardized reporting of GMFM versions, assessment dimensions, and scoring methods.

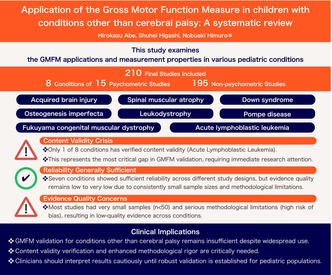

**What this paper adds**

- Content validity was tested for only one of eight paediatric conditions studied.
- Evidence quality was consistently low despite widespread use across 210 studies found.
- Reliability was good across conditions but validity evidence remains largely lacking.
- Many studies failed to report complete Gross Motor Function Measure method details.

**Abbreviations**
ALL: acute lymphoblastic leukaemia
COSMIN: Consensus‐based Standards for the selection of health Measurement Instruments
FCMD: Fukuyama congenital muscular dystrophy
GMFM: Gross Motor Function Measure
GRADE: Grading of Recommendations Assessment, Development and Evaluation
RoB: risk of bias

Gross motor function assessment is central to paediatric rehabilitation, particularly in measuring intervention effectiveness and tracking developmental progress. The Gross Motor Function Measure (GMFM) has emerged as one of the most widely used assessment tools in this field. It was initially developed and validated for children with cerebral palsy (CP),^1^ and its established reliability and validity for CP have made it a standard evaluation method both in clinical practice and in research settings.^2^, ^3^

Two versions of the GMFM are currently in use: the original 88‐item version (GMFM‐88) and a 66‐item version (GMFM‐66). The GMFM‐88 evaluates gross motor function across five dimensions: (A) lying and rolling; (B) sitting; (C) crawling and kneeling; (D) standing; and (E) walking, running, and jumping.^3^ For the GMFM‐88, the scores can be reported either as raw scores or as percentage scores, and they can be applied to children with various motor impairments other than CP.^3^ The GMFM‐66, developed through a Rasch analysis, converts ordinal scores into interval‐level measurements, providing a more precise assessment of gross motor function progression specifically for children with CP.^4^ Consequently, it is not recommended for use with children who have motor impairments other than CP.

The GMFM‐88 items were selected on the basis of clinicians’ knowledge of gross motor development in children with CP.^1^, ^3^ The substantial allocation of items to dimensions A, B, and C (51 items) and the 4‐point ordinal measurement scale reflect the tool's design to detect small changes in gross motor function, particularly in children with CP who may not achieve independent walking. These design characteristics distinguish the GMFM from standardized measures of motor development and motor function normed on typically developing children.

The measurement properties of both GMFM versions have been extensively evaluated through multiple validation studies. Research has consistently demonstrated strong reliability,^2^, ^5^, ^6^, ^7^ construct validity,^6^, ^8^, ^9^, ^10^ and responsiveness^5^, ^8^, ^10^ across different age groups and severity levels. A systematic review examining measures of motor and functional skills in CP populations confirmed that the GMFM‐88 and GMFM‐66 possess adequate reliability, validity, and measurement precision.^11^

Although the GMFM has demonstrated strong measurement properties for CP assessment, its validation for other paediatric conditions with motor impairments remains limited. The GMFM user's manual documents measurement properties for various conditions, including acquired brain injury, Down syndrome, spinal muscular atrophy, and several other paediatric conditions.^3^ Many clinicians and researchers have expanded its use beyond the documented conditions, suggesting its potential utility as a motor function assessment tool for different paediatric conditions. However, while the GMFM's use has broadened, the quality of its measurement properties for conditions other than CP has not been systematically evaluated.

The growing adoption of the GMFM across different paediatric conditions raises important questions about its validity and reliability in these new contexts. Understanding the tool's effectiveness across different conditions is crucial for ensuring accurate assessment and appropriate clinical decision‐making.

This systematic review addresses this knowledge gap by examining the application of GMFM in paediatric conditions other than CP. Its specific objectives are: (1) to determine the measurement properties and clinical utility of the GMFM in children with conditions other than CP; (2) to examine whether the GMFM can detect changes in gross motor function when applied as an outcome measure; (3) to identify the intended use of the GMFM in the studies; and (4) to determine why the GMFM was used to assess gross motor function.

## METHOD

### Registration

This systematic review has been registered in the PROSPERO database (CRD42023430171).^12^ The review was reported according to the updated Preferred Reporting Items for Systematic Reviews and Meta‐Analysis (PRISMA) statement guidelines^13^ and conducted using the Consensus‐based Standards for the selection of health Measurement Instruments (COSMIN) methodology for systematic reviews of patient‐reported outcome measures and COSMIN methodology for assessing the content validity of patient‐reported outcome measures.^14^, ^15^

### Study inclusion and exclusion criteria

This review aims to explore the use of the GMFM in children with conditions other than CP. The GMFM items cover the gross motor function to be reached by age 5 years in children with typical development and the GMFM is intended for use with children and adolescents with CP. Therefore, it was considered appropriate to apply the GMFM to children with conditions other than CP. The following selection criteria were established: (1) the participants were children aged 0 to 18 years with conditions other than CP; (2) the studies were published in peer‐reviewed journals; and (3) the studies involved the use of the GMFM. We excluded studies involving: (1) children diagnosed solely with CP or with CP in combination with other conditions; and (2) children under 2 years old who were later diagnosed with CP. Additionally, review articles, systematic reviews, protocol articles, and conference posters were excluded. If a study included children with various conditions, including CP, we only considered GMFM data for participants without CP.

### Search strategy and study selection

The search strategy was developed by the first and last authors. Searches included MEDLINE, PubMed, CINAHL, Web of Science, and Cochrane Library. All database searches were conducted on 3rd April 2023, using the following search terms for all databases: ‘gross motor function measure’ OR ‘gross motor function’ OR ‘GMFM’. No filters were applied for study design or publication date to ensure comprehensive retrieval of relevant articles.

The first and second authors conducted all screenings independently. Primary screening was based on the title and abstract of the articles, while secondary screening was performed after obtaining the full text. During secondary screening, studies examining measurement properties were specifically identified to evaluate the measurement characteristics of GMFM in different paediatric conditions. In cases of disagreement among the reviewers, a decision was made through discussion with the third author. Additionally, the references from the selected articles and hand‐searching were reviewed to identify additional articles meeting the eligibility criteria.

A systematic screening process was established for non‐English and non‐Japanese publications using two different artificial intelligence (AI) tools. The first author conducted screening using Claude AI (https://www.anthropic.com/claude), while the second author used NotebookLM (https://notebooklm.google.com/). For translation of these publications, we used the ‘English’ prompt shown in Table S1, which instructed the AI tools to provide outputs in English. This approach enabled us to perform screening and data extraction from these non‐English and non‐Japanese articles. We chose this dual‐AI approach to reduce potential errors in content interpretation and translation. Both authors met to discuss and resolve any differences in their findings.

### Data extraction and quality assessment

The first or second author performed data extraction, and the data were then checked by the two authors. The extracted data included characteristics of the study (author, year of publication, country, study design), characteristics of the participants (condition, sample size, age), the type of GMFM used (GMFM‐88, GMFM‐66, and modified GMFM), the purpose for using the GMFM, and the rationale for GMFM selection. In cases where the modified GMFM was used, the clinical utility of the assessment based on the ‘*CanChild* Outcome Measures Rating Form’^16^ was outlined.

We extracted studies that validated measurement properties and conducted a risk of bias (RoB) assessment. For evaluating the measurement properties of the GMFM by condition, we used the COSMIN methodology for systematic reviews of patient‐reported outcome measures^14^, ^17^ and the COSMIN methodology for assessing the content validity of patient‐reported outcome measures.^18^ However, because the GMFM is a performance‐based outcome measure, we followed the COSMIN Risk of Bias tool to assess the quality of studies on reliability and measurement error of outcome measurement instrument.^19^ Specifically, we replaced the standard boxes for reliability and measurement error from the COSMIN Risk of Bias checklist with the COSMIN Risk of Bias tool designed for assessing the quality of studies on reliability and measurement error in performance‐based outcome measures.^19^, ^20^

### COSMIN Risk of Bias assessment by condition and GMFM version

Given that the extent of measurement property validation across different paediatric conditions and GMFM versions was previously unknown, we conducted separate COSMIN assessments for each condition and GMFM version combination to systematically map the evidence base. This approach was necessary to address the substantial heterogeneity observed, including the diversity of paediatric conditions and GMFM versions used across studies (GMFM‐88, GMFM‐66, and modified GMFM). By analysing each condition–version combination separately, we aimed to determine which paediatric conditions and GMFM versions had sufficient measurement property evidence and which required further validation.

### Data extraction and identification of measurement properties

We extracted detailed information on the measurement properties evaluated from each study included in this review. The measurement properties were classified into patient‐reported outcome measure development, content validity, structural validity, internal consistency, cross‐cultural validity/measurement invariance, reliability, measurement error, criterion validity, hypotheses testing for construct validity, and responsiveness.^14^, ^17^ In cases where the study populations differed or different comparison measures were used to assess construct validity, we completed the same measurement property box multiple times to ensure accurate RoB assessment for each specific context. For example, when a study examined construct validity using two different comparison outcome measures, we filled out the construct validity box separately for each outcome measure because the methodological quality and potential biases may differ depending on the comparison measure used. This approach enabled a more detailed evaluation of each measurement property across various contexts, as different study conditions may present different methodological challenges and RoB. Additionally, for studies using different GMFM versions, we analysed measurement properties separately by version to account for potential differences in measurement performance and associated methodological considerations across versions.

### COSMIN Risk of Bias assessment and evaluation of updated criteria for good measurement properties

The RoB was assessed using the COSMIN checklist with a 4‐point scale: ‘very good’, ‘adequate’, ‘doubtful’, and ‘inadequate’.^14^, ^17^ Each item in a measurement property box was rated according to these criteria. We determined the overall methodological quality score for each measurement property by applying the ‘worst score counts’ principle, where the lowest rating among all items in a box was used as the final score.^14^ For example, if a measurement property box contained mostly ‘very good’ ratings but one ‘doubtful’ rating, the overall score for that property would be ‘doubtful’. Each study's findings were evaluated against the updated criteria for good measurement properties. Each result was rated as sufficient (+), insufficient (−), or indeterminate (?) on the basis of specific criteria for each measurement property.^14^, ^17^

Following current best practice recommendations,^21^ the RoB assessment was conducted using the COSMIN checklist with detailed item‐level evaluation. All assessment results, including individual item ratings and justifications, were reported completely rather than using summary scores only. Studies with high RoB were identified but were not automatically excluded from analysis.

The RoB assessment evaluates the methodological rigor of how studies were conducted (study design, sample size adequacy, statistical methods), while the criteria for good measurement properties evaluate whether the obtained results meet predetermined thresholds or hypotheses for acceptable measurement performance (e.g. correlation coefficients, reliability values).

### Assessment of clinical and methodological heterogeneity

We systematically evaluated clinical and methodological heterogeneity across the included studies to inform our analytical approach and determine the appropriateness of meta‐analysis.

The clinical heterogeneity assessment examined participant characteristics and disease severity across studies, including: (1) demographic variations (age ranges, sample sizes); (2) disease‐specific factors (severity levels, functional status, ambulatory capacity); and (3) condition‐specific characteristics (genetic subtypes, injury intervals). The methodological heterogeneity assessment evaluated differences in assessment methods (interrater reliability vs intrarater reliability, discriminant validity vs convergent validity for construct validity), and GMFM version differences (GMFM‐88, GMFM‐66, and modified GMFM).

On the basis of this heterogeneity assessment, we determined that the substantial clinical diversity across different paediatric conditions and considerable methodological variations precluded meaningful quantitative meta‐analysis. Therefore, we adopted a structured narrative synthesis approach, organizing results by diagnostic condition and GMFM version to ensure appropriate interpretation of findings in each clinical context.

### Evidence synthesis and modified GRADE approach

To assess the overall quality of each measurement property across all studies, we qualitatively pooled the results for each diagnostic group and GMFM version combination and compared them against the criteria for good measurement properties, taking into account the heterogeneity identified in the previous assessment. This condition‐specific pooled assessment resulted in an overall rating for each measurement property as sufficient (+), insufficient (−), inconsistent (±), or indeterminate (?).^14^, ^17^

Subsequently, we used a modified Grading of Recommendations Assessment, Development and Evaluation (GRADE) approach^14^, ^17^ to assess the quality of evidence for each measurement property of the GMFM across different diagnostic groups and GMFM versions. This approach considers four factors: RoB (methodological quality assessed by COSMIN checklist), inconsistency (unexplained heterogeneity across studies), imprecision (total sample size with downgrading for *n* < 100), and indirectness (differences between study populations/contexts and review targets).^14^, ^17^ For each factor, the quality of evidence can be downgraded by one or two levels depending on the severity of the issue. The assessment process begins with a ‘high’ quality rating, which can be downgraded on the basis of these factors. The final quality rating for each measurement property in each diagnostic group is categorized as ‘high,’ ‘moderate,’ ‘low,’ or ‘very low’.^14^, ^17^ The first and second authors independently conducted each measurement property assessment, with any disagreements resolved through discussion or by consulting the last author.

## RESULTS

### Search results

Figure S1 shows the search results. We found 15334 articles and removed 9262 duplicates. After screening the titles and abstracts of the remaining 6072 articles, we selected 427. A full‐text review of the 427 articles led to the selection of 205 studies. Additionally, we found two relevant studies through the authors’ previous research project and three studies from the reference list of an included article. This resulted in the final selection of 210 studies, with 15 focusing on GMFM measurement properties.

Although our inclusion criteria specified participants aged 0 to 18 years, some studies^22^, ^23^ reported mean or median ages below 18 years while including participants over 18 years. We included these studies as they aligned with our primary focus on paediatric conditions, and most participants fell within our target age range.

### Clinical utility of GMFM

Table 1 presents the clinical utility characteristics of the various GMFM versions. Our analysis revealed four specialized versions of the GMFM adapted for specific conditions, including the GMFM for Fukuyama congenital muscular dystrophy (GMFM‐FCMD),^24^ the GMFM for Down syndrome (GMFM‐DS),^25^ the GMFM for acute lymphoblastic leukaemia (GMFM‐ALL),^26^ and an infant‐specific version (GMFM in infancy).^27^ These adaptations differ from the standard GMFM‐88 and GMFM‐66 in several key aspects. The GMFM‐ALL focuses exclusively on advanced motor functions (standing and walking dimensions),^26^ while the GMFM‐DS incorporates parent‐reported scoring (‘R’ marking) and standard scoring.^25^ The GMFM‐FCMD uses a modified 68‐item set based on Rasch analysis and clinical decisions specific to the motor characteristics of children with FCMD,^24^ and the GMFM in infancy features adjusted scoring criteria with partial credits for assisted movements and developmental progression.^27^ Among these four versions, measurement properties were verified for GMFM‐FCMD,^24^ GMFM‐DS,^25^ and GMFM‐ALL.^26^

**TABLE 1:** Clinical utility of the GMFM.

Type	Dimensions and number of items	Scoring	Interpretability	Special equipment	Clarity of instructions	Time required	Examiners’ qualifications	Cost
GMFM‐88^1^	A. Lying and rolling (17)	4‐point original scale:	Order scale: raw (range 0–264) or percentage scores for each dimension or total	Standard physiotherapy equipment (mat, bench, toys), stairs (5+ steps), smooth floor, large space (4.5m minimum)	Detailed guidelines (chapter 6), standardized 4‐point scoring with specific descriptors, practice recommended, online certification available (GMFM Criterion Test)	45–60 minutes	Paediatric therapists with motor assessment experience, GMFM training/certification recommended	GMFM manual (GBP/£85.00)
	B. Sitting (20)	0 (does not initiate the task) to 3 (completes the task)						GMFM Criterion Test (CA$60)
	C. Crawling and kneeling (14)							
	D. Standing (13)							
	E. Walking, running, and jumping (24)							
GMFM‐66^4^	66 items identified by Rasch analysis and clinical decisions from 88 items	4‐point original scale and not tested	Interval scale (range 0–100): use Rasch analysis software (GMFM app or GMAE‐2)	Same as GMFM‐88	Same as GMFM‐88	Less than GMFM‐88	Same as GMFM‐88	GMFM App + (CA$150–1200)
GMFM‐ALL^26^	D: Standing (7)	4‐point original scale:	Order scale: percentage scores for each dimension or total	NA	NA	15–30 minutes	Physical therapists or occupational therapists	NA
	E: Walking, running, and jumping (13)	0 (does not initiate the task) to 3 (completes the task)		(Standard GMFM equipment assumed)	(following GMFM manual guidelines)			(GMFM manual required)
	(item list in supplementary material of original paper)	Special considerations:						
		Credits given for lower‐level items when higher‐level items performed (on the basis of guidelines for Down syndrome)						
GMFM‐DS^25^	Same as GMFM‐88	4‐point original scale:	Same as GMFM‐88	NA	NA	NA	Physical therapists and/or occupational therapists (with paediatric experience recommended)	NA
		0 (does not initiate the task) to 3 (completes the task)		(Standard GMFM equipment assumed)	(following GMFM manual guidelines)			(GMFM manual required)
		Special considerations:						
		Reported scoring:						
		Record ‘R’ for items regularly performed at home but not during assessment						
		Calculate additional score incorporating parent‐reported items (‘R’ marked)						
GMFM‐FCMD^24^	68 items identified by Rasch analysis and clinical decisions from 88 items (item list in supplementary material of original paper)	Same as GMFM‐88	Order scale: raw scores (range 0–204)	NA	Simple instructions appropriate for severe cognitive impairment	30 minutes (range 13–77)	Physical therapists	NA
				(Standard GMFM equipment assumed)				(GMFM manual required)
GMFM in Infancy^27^	A: Lying and rolling (16)	4‐point original scale:	Order score: percentage scores for each dimension or total	Standard GMFM equipment, age‐appropriate toys, minimum 6m × 4m area	NA	NA	Trained GMFM assessors	NA
	B: Sitting (17)	0 (does not initiate the task) to 3 (completes the task)			(following GMFM manual guidelines)			(GMFM manual required)
	C: Crawling and kneeling (12)	Special considerations:						
	D: Standing (11)	Award 80% score for dimension A when crawling is achieved						
	E: Walking, running, and jumping (21)	Award points for creeping if crawling is achieved						
	(item list in supplementary material of original paper)	Consider both voluntary and involuntary rolling						
		Give partial scores for assisted walking/jumping items						

Abbreviations: GMAE‐2, Gross Motor Ability Estimator‐2; GMFM, Gross Motor Function Measure; GMFM‐ALL, Gross Motor Function Measure for acute lymphoblastic leukemia; GMFM‐DS, Gross Motor Function Measure for Down syndrome; GMFM‐FCMD, Gross Motor Function Measure for Fukuyama congenital muscular dystrophy; NA, not available.

With regard to practical implementation characteristics, these specialized versions accommodate diverse clinical needs. Administration time varies considerably among versions, ranging from 15 to 30 minutes for the GMFM‐ALL, while the GMFM‐FCMD demonstrates flexibility in administration time of 13 to 77 minutes on the basis of individual patient needs and cognitive abilities. Equipment needs remain consistent with standard GMFM protocols across all specialized versions, using basic physiotherapy equipment including mats, benches, and appropriate toys. However, space requirements may differ, with the GMFM in infancy requiring a minimum 6m × 4m area for safe assessment of developing motor skills. Examiners were therapists or those who had received GMFM training. Cost considerations primarily involve the basic GMFM user's manual, as these specialized versions do not require additional software purchases. None of these assessments provide dedicated score sheets, requiring clinicians to refer to the original research papers for item details.

### GMFM measurement properties across multiple conditions

Tables 2, 3, and S2 to S9 present the characteristics of the 15 studies investigating GMFM measurement properties. The studies of traumatic brain injury^28^, ^29^ and head injury^1^ were integrated and analysed under the category of acquired brain injury. For consistency, we use ‘acquired brain injury’ as the umbrella term encompassing traumatic brain injury and other acquired brain injuries, reflecting current paediatric clinical practice.^30^ A total of 15 studies evaluated the measurement properties of GMFM across eight different conditions: acquired brain injury,^1^, ^28^, ^29^, ^31^ spinal muscular atrophy,^32^, ^33^, ^34^, ^35^ FCMD,^22^, ^24^ Down syndrome,^25^ osteogenesis imperfecta,^36^ ALL,^26^ leukodystrophy,^23^ and Pompe disease.^37^

**TABLE 2:** Measurement property study characteristics.

Diagnosis	Study	Year	*n*	Mean age (SD); range	Type of GMFM
Head injury	Russell et al.^1^	1989	25^a^	NA	Original GMFM (GMFM‐88)
Traumatic brain injury	Thomas‐Stonell et al.^28^	2006	27^a^	12.5 (4.5); 4–18 years^b^	GMFM^d^
	Linder‐Lucht et al.^29^	2007	73	11.4 (5.1); 0.8–18.9 years	GMFM‐88 total (raw)
					GMFM‐66
Acquired brain injury	Storm et al.^31^	2020	110	10.8 (4.1) years	GMFM‐88 D–E (%)
					GMFM‐88 total (%)
Spinal muscular atrophy	Iannaccone et al.^32^	2002	10	7.4; 2–14 years	GMFM‐88 A–B
					GMFM‐88 total
	Iannaccone et al.^33^	2003	34	2–17 years	GMFM‐88 A–E (raw and %)
					GMFM‐88 total (raw and %)
	Nelson et al.^35^	2006	40	10.90 (3.57) years	GMFM‐88 A–E (raw)
					GMFM‐88 total (raw)
	Chen et al.^34^	2014	38	8–31 years^c^	GMFM^d^
Fukuyama congenital muscular dystrophy	Sato et al.^22^	2017	41	8.6; 0.6–24.4 years	GMFM‐88 total (raw)
	Sato et al.^24^	2020	15	7.0; 2.0–15.3 years	GMFM‐FCMD
					GMFM‐88 total (raw)
Down syndrome	Russell et al.^25^	1998	123	28.7; 1.7–72.0 months	GMFM‐DS^e^ total (%)
					GMFM‐DS A–E (%)
					GMFM‐88 total (%)
					GMFM‐88 A–E (%)
Osteogenesis imperfecta	Ruck‐Gibis et al.^36^	2001	19	7.89 years; 8 months–17 years 11 months	GMFM‐88 A–E (%)
					GMFM‐88 total (%)
Acute lymphoblastic leukaemia	Wright et al.^26^	2007	91	Median 8.5; 2.8–15.9 years^c^	GMFM‐ALL (%)
					GMFM‐88 D–E (%)
					GMFM‐88 total (%)
Leukodystrophy	Gavazzi et al.^23^	2021	21	9.6 (11.0); 1.3–52.5 years	GMFM‐88 total (raw and %)
Pompe disease	Duong et al.^37^	2022	110	5.2 (3.6); 1.0–15.5 years	GMFM‐88 total (%)

*Note*: Ages reported as in original studies (with decimals or years and months). Abbreviations: GMFM, Gross Motor Function Measure; GMFM‐ALL, Gross Motor Function Measure for acute lymphoblastic leukemia; GMFM‐DS, Gross Motor Function Measure for Down syndrome; GMFM‐FCMD, Gross Motor Function Measure for Fukuyama congenital muscular dystrophy; NA, not available; SD, standard deviation.

^a^ Number for GMFM assessment.

^b^ Age of all participants.

^c^ Age of subset participants.

^d^ The original text was retained and data were combined under GMFM‐88.

^e^ GMFM‐DS refers to GMFM‐88 using reported scoring method for children with Down syndrome.

**TABLE 3:** Methodological quality, ratings, and results of the GMFM in children with conditions other than CP by study.

Diagnosis	Measurement property	Study	Design^a^	*n*	Results	COSMIN RoB	Rating
Acquired brain injury	Reliability	Linder‐Lucht et al.^29^	Test–retest	10	ICC = 0.99	Doubtful	+
			(GMFM‐66 and GMFM‐88)				
	Responsiveness	Russell et al.^1^	Comparison with other groups	25	There was a significant difference between change scores for the head injury and CP groups.	Doubtful	+
		Thomas‐Stonell et al.^28^	Comparison before and after Neurorehabilitation Programme	27	Standardized response mean = 0.62	Doubtful	+
		Linder‐Lucht et al.^29^	Comparison with judgement by person	70	GMFM‐88: *r* = 0.531–0.737	Adequate	+
			(GMFM‐66 and GMFM‐88)		GMFM‐66: *r* = 0.536–0.679		
			Comparison by duration of injury	73	Changes in gross motor function became fewer as the interval between brain injury and baseline increased.	Doubtful	+
			(GMFM‐66 and GMFM‐88)				
		Storm et al.^31^	Comparison before and after Robot‐assisted gait training	110	GMFM‐88 (%): MCID = 1.1–5.3 (overall)	Adequate	+
					Dimension D (%): MCID = 2.3–6.5 (overall)		
					Dimension E (%): MCID = 2.8–6.5 (overall)		
Spinal muscular atrophy	Reliability	Iannaccone et al.^32^	Interrater	10	*κ* = 0.72	Doubtful	+
			(GMFM‐88 A‐B)				
			Interrater	10	In the Friedman test for repeated measurements, there were no significant differences between the total GMFM.	Doubtful	?
			(GMFM‐88 Total)				
		Iannaccone et al.^33^	Intrarater	34	ICC = 0.96–0.98 (total and A–E)	Adequate	+
		Chen et al.^34^	Intrarater at‐home	10	ICC = 0.9938	Inadequate	+
			Intrarater in‐hospital	28	ICC = 0.9968	Inadequate	+
	Construct validity	Nelson et al.^35^	Comparison with Quantitative Muscle Testing	40	*r* = 0.84 (total)	Very good	+
					*r* = 0.77–0.86 (A–E)		
			Comparison between ambulatory status	40	The walkers were significantly higher than the non‐walkers.	Adequate	+
			Comparison between BiPAP ventilation status	40	The BiPAP group was significantly lower than the non‐BiPAP group.	Doubtful	+
		Chen et al.^34^	Comparison with Modified Hammersmith Functional Motor Scale	38	*r* = 0.95	Adequate	+
			Comparison between in‐hospital and at‐home	38	There is a significant difference between the home and the hospital.	Doubtful	−
			Calculated intrasubject differences	3	There is a significant difference between the home and the hospital.	Inadequate	−
Fukuyama congenital muscular dystrophy	Structural validity	Sato et al.^24^	Structural validity	100^b^	Rasch analysis of the GMFM‐FCMD identified 18 misfit items, and after clinical review, 20 items were ultimately excluded, resulting in a modified 68‐item GMFM version.	Inadequate	+
			(GMFM‐FCMD)				
	Reliability	Sato et al.^22^	Interrater	20	ICC = 0.9739–0.9979	Adequate	+
	Construct validity	Sato et al.^22^	Comparison with Modified Ueda classification	41	rho = 0.930	Doubtful	+
			Comparison between three phenotype groups	41	GMFM scores were significantly higher for the mild phenotype, followed in order by the typical and severe phenotypes.	Doubtful	+
			Comparison with NPPV status	41	NPPV group was significantly lower than non‐NPPV group.	Doubtful	+
		Sato et al.^24^	Comparison with Ueda classification	15	*r* = 0.935	Doubtful	+
			(GMFM‐FCMD)				
			Comparison with GMFM‐88	15	*r* = 0.9951	Doubtful	+
			(GMFM‐FCMD)				
Down syndrome	Reliability	Russell et al.^25^	Test–retest	22	Standard scoring	Doubtful	+
			(GMFM‐DS and GMFM‐88)		ICC = 0.95 (total)		
					ICC = 0.62–0.98 (A–E)		
					Reported scoring		
					ICC = 0.96 (total)		
					ICC = 0.87–0.99 (A–E)		
			Interrater	22	Standard scoring	Doubtful	+
			(GMFM‐DS and GMFM‐88)		ICC = 0.96 (total)		
					ICC = 0.73–0.98 (A–E)		
					Reported scoring		
					ICC = 0.98 (total)		
					ICC = 0.82‐0.99 (A–E)		
	Responsiveness	Russell et al.^25^	Comparison with BSID‐II	110	Standard scoring	Adequate	+
			(GMFM‐DS and GMFM‐88)		GMFM demonstrated larger changes in lower severity groups, while BSID‐II showed no consistent pattern across groups.		
					Reported scoring		
					Enhanced responsiveness with more marked trends across age/severity groups compared with standard scoring.		
			Comparison with judgement by person	117	Standard scoring	Adequate	−
			(GMFM‐DS and GMFM‐88)	(Parent)	Total GMFM correlations below criterion (parent *r* = 0.16, intervenor *r* = 0.24, video *r* = 0.23; criterion *r* > 0.60).		
				80	Reported scoring		
				(Intervenor)	Improved correlations (parent *r* = 0.52, intervenor *r* = 0.40, video *r* = 0.34), stronger than standard administration but still below criterion for total score.		
				30			
				(Video)			
			Comparison among ages and among severities	123	Standard scoring	Inadequate	+
			(GMFM‐DS and GMFM‐88)		Significant gradient ‐ group 1 (young/mild): 15% improvement vs group 4 (older/moderate‐severe): 7% improvement.		
					Reported scoring		
					More pronounced gradient ‐ group 1: 16% improvement vs group 4: 6% improvement.		
Osteogenesis imperfecta	Reliability	Ruck‐Gibis et al.^36^	Interrater	19	ICC = 0.98–0.99	Very good	+
			Intrarater	19	ICC = 0.99	Very good	+
Acute lymphoblastic leukaemia	Content validity	Wright et al.^26^	Relevance		Selected items demonstrated high clinical utility scores (≥19) based on three paediatric oncology physiotherapists’ evaluation, with strong correlations with the original GMFM (D: *r* = 0.977, E: *r* = 0.983, both *p* < 0.001).	Doubtful	+
			(GMFM‐ALL)				
			Comprehensiveness		GMFM‐ALL retained 20 items (D: 7, E: 13) from the original 37 items, showing high reliability (0.90) and accommodating various functional levels including children with lower functioning.	Doubtful	+
			(GMFM‐ALL)				
	Reliability	Wright et al.^26^	Interrater	13	Generalizability coefficients = 0.99	Doubtful	+
			(GMFM‐ALL and GMFM‐88)				
			Test–retest	13	Generalizability coefficients = 0.94–0.97	Doubtful	+
			(GMFM‐ALL and GMFM‐88)				
	Construct validity	Wright et al.^26^	Comparison with GMFM‐88 D–E	91	Dimension D: *r* = 0.977	Adequate	+
			(GMFM‐ALL)		Dimension E: *r* = 0.983		
	Responsiveness	Wright et al.^26^	Comparison between time points	39	There was a significant change in scores between the first and second time.	Inadequate	+
			(GMFM‐ALL and GMFM‐88)				
			Comparison between age groups	39	There was a significant difference in the change in scores between younger and older children for dimension D scores. There was no significant difference in dimension E scores.	Inadequate	−
			(GMFM‐ALL and GMFM‐88)				
Leukodystrophy	Reliability	Gavazzi et al.^23^	Interrater	10	ICC = 0.99 (total)	Adequate	+
					ICC = 0.98 (A) to 0.99 (B–E)		
			Intrarater	6	ICC = 0.99 (total and A–E)	Adequate	+
	Measurement error	Gavazzi et al.^23^	Statistical analysis	21	Limit of agreement = −7.8 to 4.3 (interrater)	Adequate	?
					Limit of agreement = −2.3 to 2.7 (intrarater)		
Pompe disease	Measurement error	Duong et al.^37^	Statistical analysis	90	Overall	Doubtful	?
					Mean change: 3.7 ± 17.5		
					MDC range: 10.0–23.3		
					Age < 2 years (*n* = 19)		
					Mean change: 21.1 ± 14.1		
					MDC range: 5.7–13.3		
					Age ≥ 2 years (*n* = 71)		
					Mean change: −0.9 ± 15.3		
					MDC range: 10.8–25.2		
	Responsiveness	Duong et al.^37^	Effect size	90	Overall	Doubtful	+
					Effect size: 0.11		
					Age < 2 years (*n* = 19)		
					Effect size: 1.11		
					Age ≥ 2 years (*n* = 71)		
					Effect size: −0.03		

Abbreviations: +, sufficient rating; ?, indeterminate rating; −, insufficient rating; BiPAP, bilevel positive airway pressure; BSID‐II, Bayley Scales of Infant Development, Second Edition; COSMIN RoB, COnsensus‐based Standards for the selection of health Measurement INstruments Risk of Bias; CP, cerebral palsy; GMFM, Gross Motor Function Measure; GMFM‐ALL, Gross Motor Function Measure for acute lymphoblastic leukemia; GMFM‐DS, Gross Motor Function Measure for Down syndrome; GMFM‐FCMD, Gross Motor Function Measure for Fukuyama congenital muscular dystrophy; ICC, intraclass correlation coefficient; *κ*, kappa coefficient; MCID, minimal clinically important difference; MDC, minimal detectable change; NPPV, non‐invasive positive pressure ventilation; *r*, correlation coefficient; rho, Spearman's rank correlation coefficient.

^a^ Unless otherwise specified, all analyses were conducted using the GMFM‐88. Where different versions were used, they are explicitly indicated in parentheses after the relevant analysis (e.g. GMFM‐66 and GMFM‐88, GMFM‐FCMD, GMFM‐ALL).

^b^ Sample size was reported as 100, but may represent multiple assessments of the same participants from the study by Sato et al.^22^ (*n* = 41).

Substantial heterogeneity was observed across all condition groups, including diverse diagnostic terminology, varying assessment timepoints, different GMFM versions, and methodological variations in measurement property evaluations, making quantitative meta‐analysis unfeasible. Additionally, while some conditions had studies examining only single measurement properties (single boxes), others used multiple approaches to evaluate the same property (multiple boxes) (e.g. responsiveness assessed through age‐based comparisons, severity‐based comparisons, and correlations with external instruments). Therefore, results were synthesized through structured narrative synthesis. Complete details of all RoB assessments are provided in Tables S2 to S9.

### Acquired brain injury studies

Four measurement property studies in acquired brain injury/traumatic brain injury were identified, with sample sizes ranging from 25 to 110 participants (Tables 2, 3, and S2).^1^, ^28^, ^29^, ^31^ Participants were mainly children aged 10 months to 18 years 11 months. The studies included diverse diagnostic terminology (acquired brain injury, traumatic brain injury, and head injury) and varying time from injury to assessment (acute phase to more than 11 years post‐injury). Methodological heterogeneity was observed in different GMFM versions (GMFM‐88, GMFM‐66, and unspecified GMFM), diverse measurement property evaluation approaches, and different statistical analysis methods for responsiveness assessment.

The reliability was examined in one study using test–retest methodology for both GMFM‐66 and GMFM‐88, showing excellent levels, but COSMIN RoB was rated as doubtful.^29^ The responsiveness was evaluated in multiple studies through various approaches including comparisons with other diagnostic groups,^1^, ^29^ intervention‐based assessments,^28^, ^31^ and correlations with external judgements,^29^ demonstrating sufficient responsiveness and moderate to strong associations. However, the methodological quality showed considerable variation, with most studies ranging from adequate to doubtful RoB ratings, and only two studies (two boxes) achieving adequate RoB ratings. All measurement properties received sufficient (+) ratings.

The reliability evidence was very low quality owing to small sample size (*n =* 10) and methodological limitations in the single available study, and the overall rating was rated as sufficient (+). The responsiveness evidence was low quality, downgraded for varying study quality and analysis of mixed diagnostic groups, and was considered sufficient (+) rating for detecting changes during rehabilitation (Table 4).

**TABLE 4:** Evidence synthesis and quality rating of measurement properties for the GMFM in children with conditions other than cerebral palsy.

Diagnosis	Type of GMFM	Content validity		Structural validity		Reliability		Measurement error		Construct validity		Responsiveness	
		GRADE	Rating	GRADE	Rating	GRADE	Rating	GRADE	Rating	GRADE	Rating	GRADE	Rating
Acquired brain injury	GMFM‐88					Very low	+					Low	+
	GMFM‐66					Very low	+					Low	+
Spinal muscular atrophy	GMFM‐88					Low	+			Low	+		
Fukuyama congenital muscular dystrophy	GMFM‐88					Very low	+			Very low	+		
	GMFM‐FCMD			Very low	+					Very low	+		
Down syndrome	GMFM‐88 GMFM‐DS					Very low	+					Very low	±
Osteogenesis imperfecta	GMFM‐88					Low	+						
Acute lymphoblastic leukaemia	GMFM‐88					Very low	+					Very low	±
	GMFM‐ALL	Moderate	+			Very low	+			Very low	+	Very low	±
Leukodystrophy	GMFM‐88					Very low	+	Very low	?				
Pompe disease	GMFM‐88							Very low	?			Very low	+

Abbreviations: +, sufficient rating; ±, inconsistent; ?, indeterminate rating; GMFM, Gross Motor Function Measure; GMFM‐ALL, Gross Motor Function Measure for acute lymphoblastic leukemia; GMFM‐DS, Gross Motor Function Measure for Down syndrome; GMFM‐FCMD, Gross Motor Function Measure for Fukuyama congenital muscular dystrophy; GRADE, Grading of Recommendations Assessment, Development and Evaluation.

### Spinal muscular atrophy studies

Four measurement property studies in spinal muscular atrophy were identified, with sample sizes ranging from 10 to 40 participants (Tables 2, 3, and S3).^32^, ^33^, ^34^, ^35^ Participants were mainly children, but some studies included a wide age range (8–31 years).^34^ The studies included participants with spinal muscular atrophy type II and type III, with functional levels ranging from walkers to non‐walkers. Methodological heterogeneity was observed in different GMFM versions (GMFM‐88 total, dimensions A and B, dimensions A to E, and unspecified GMFM), diverse measurement property evaluation approaches, and different assessment environments (hospital vs home settings).

The reliability was examined in three studies (five boxes) using both interrater and intrarater reliability for GMFM‐88, showing good to excellent levels, but methodological quality based on COSMIN criteria varied from adequate to inadequate.^32^, ^33^, ^34^ The construct validity was evaluated in two studies (six boxes) through correlations with existing measures^34^, ^35^ and known‐groups comparisons,^34^, ^35^ confirming strong correlations and expected differences between walking and non‐walking groups. However, the methodological quality showed considerable variation, with COSMIN RoB ranging from very good to inadequate, and measurement property ratings showing diverse results from sufficient (+) to insufficient (−).

The reliability evidence was low quality, downgraded for small total sample size (*n =* 82) and varying methodological quality across studies, and the overall rating was rated as sufficient (+). The construct validity evidence was low quality owing to similar limitations, and was considered sufficient (+) for consistent findings across studies with very good to adequate methodology (Table 4).

### Fukuyama congenital muscular dystrophy studies

Two measurement property studies in FCMD were identified with sample sizes ranging from 15 to 41 participants (Tables 2, 3, and S4).^22^, ^24^ The studies examined children and young adults aged 7 months to 24 years 5 months. Both studies were conducted in Japan and showed similar genetic mutation patterns. Methodological heterogeneity was observed in different GMFM versions used (GMFM‐88 and GMFM‐FCMD), diverse measurement property evaluation approaches, and different statistical analysis methods for construct validity assessment.

The structural validity of GMFM‐FCMD was examined in one study using Rasch analysis, showing inadequate methodological quality and receiving a sufficient (+) rating.^24^ The interrater reliability of GMFM‐88 was evaluated in one study, demonstrating a sufficient (+) rating with adequate methodological quality.^22^ The construct validity (GMFM‐88 and GMFM‐FCMD) was assessed in multiple studies through various approaches including comparisons with classification systems,^22^, ^24^ phenotype groups,^22^ and clinical status indicators,^22^ showing strong correlations. However, methodological quality showed variation, with all construct validity studies receiving doubtful RoB ratings. All measurement properties received sufficient (+) ratings.

All evidence was very low quality owing to very small sample sizes (*n =* 15–41) and limitation to studies from a single research group. Both GMFM‐88 and GMFM‐FCMD overall ratings were rated as sufficient (+) rating (Table 4).

### Down syndrome study

One measurement property study in Down syndrome was identified.^25^ This study included 123 children with Down syndrome aged 1.7 to 72.0 months (Tables 2, 3, and S5). The study evaluated both standard GMFM‐88 scoring and a modified approach (referred to as GMFM‐DS) that incorporates parent reports of children's motor abilities not demonstrated during assessment sessions. Participants showed genetic heterogeneity, with most children (81.1%) diagnosed with trisomy 21, while others had different genetic backgrounds or unknown types. Methodological heterogeneity was observed in the evaluation of multiple measurement properties through various approaches, including test–retest and interrater reliability assessments, and responsiveness evaluations using age‐based comparisons, severity‐based comparisons, external instrument comparisons, and correlations with external judgements.

The reliability was examined using both test–retest and interrater methods, showing excellent levels with ICC values. The responsiveness was evaluated through multiple approaches, demonstrating that younger children with mild impairments showed significantly greater improvement compared with older groups, and the GMFM showed larger changes in lower severity groups compared with the Bayley Scales of Infant Development, Second Edition. However, the methodological quality based on COSMIN RoB was rated as doubtful for reliability assessments, while responsiveness evaluations showed varying quality ratings from adequate to inadequate. Most measurement properties received sufficient (+) ratings, although some construct validity assessments showed insufficient (−) results.

The reliability evidence was very low quality owing to small sample size (*n =* 22) and methodological concerns, and the overall rating was rated as sufficient (+). The responsiveness evidence was also very low quality, with inconsistent (**±**) ratings reflecting mixed findings across different age groups and functional levels (Table 4).

### Osteogenesis imperfecta study

One measurement property study in osteogenesis imperfecta was identified, with a sample size of 19 participants (Tables 2, 3, and S6).^36^ Participants were children aged 8 months to 17 years 11 months (mean age 7 years 11 months). The sample included multiple types (types I, III, and IV) of osteogenesis imperfecta severity.

The reliability was examined using a sample of 19 participants through both interrater and intrarater approaches. Both interrater and intrarater reliability showed excellent levels. The methodological quality based on COSMIN RoB was rated as very good for the reliability box. All measurement properties received sufficient (+) ratings.

The reliability evidence was low quality, downgraded only for small sample size (*n* = 19) as the study methodology was of very good quality. The overall rating was rated as sufficient (+) rating (Table 4).

### Acute lymphoblastic leukaemia study

One measurement property study in children with ALL was identified, with a total sample of 91 participants (Tables 2, 3, and S7).^26^ The study included children with a median age of 8 years 6 months (range 2 years 10 months–15 years 11 months) and incorporated both standard risk and high‐risk stratification groups. The measurement properties evaluated included content validity (relevance and comprehensiveness), interrater reliability, test–retest reliability, construct validity, and responsiveness.

The content validity was assessed through systematic quantitative analysis using a 7‐point Likert scale by three paediatric oncology physiotherapists, demonstrating sufficient (+) ratings for relevance and comprehensiveness with doubtful COSMIN RoB. The reliability showed doubtful COSMIN RoB owing to unclear blinding procedures and incomplete participant data, and achieved sufficient (+) rating with excellent generalizability coefficients. The construct validity of GMFM‐ALL had adequate COSMIN RoB with sufficient (+) rating, showing strong correlations with GMFM‐88. The responsiveness demonstrated inadequate COSMIN RoB owing to inappropriate statistical methods, resulting in mixed ratings. Time points comparisons showed sufficient (+) rating, while age group comparisons showed insufficient (−) rating.

The content validity of GMFM‐ALL evidence was moderate quality and was rated as sufficient (+). The reliability and construct validity evidence were very low quality owing to small sample sizes and methodological limitations, and both were rated as sufficient (+). The responsiveness evidence was also very low quality with inconsistent results across different comparisons and was rated as inconsistent (±) (Table 4).

### Leukodystrophy study

One measurement property study in leukodystrophy was identified, with a sample size of 21 participants (Tables 2, 3, and S8).^23^ Participants included children and adults with molecularly confirmed leukodystrophy diagnoses, aged 1 year 4 months to 52 years 6 months (mean 9 years 7 months SD 11 years). The study showed diagnostic diversity in the leukodystrophy group, including four disease types centred around Aicardi–Goutières syndrome. The study used GMFM‐88 for measurement property evaluation of reliability and measurement error.

The reliability was examined through both interrater and intrarater methodologies, showing excellent reliability coefficients. The measurement error was assessed using limits of agreement with a statistical analysis approach. The methodological quality based on COSMIN RoB showed adequate ratings for both reliability assessment and measurement error evaluation. While reliability received sufficient (+) ratings, measurement error received an indeterminate (?) rating owing to the undefined minimal important change.

The reliability evidence was very low quality owing to small sample size (*n* = 21) and single study design, and the overall rating was rated as sufficient (+). The measurement error evidence was also very low quality, with an indeterminate (?) rating owing to undefined clinical significance thresholds (Table 4).

### Pompe disease study

One measurement property study in Pompe disease was identified, with 90 participants used for measurement properties evaluation (Tables 2, 3, and S9).^37^ This study included children aged 1 year to 15 years 6 months (mean 5 years 2 months SD 3 years 7 months) with diverse motor function levels at baseline ranging from level I (walkers) to level V (restricted antigravity movement). The measurement error and responsiveness were examined in the overall population and by age groups (<2 years and ≥2 years).

The measurement error was examined using statistical analysis methodology over a 52‐week interval, with age‐dependent minimal detectable change ranges showing smaller values in children younger than 2 years and larger values in children 2 years or older. The responsiveness was evaluated through effect size calculations, demonstrating substantial variation across age groups, with higher effect sizes in children younger than 2 years and lower effect sizes in children 2 years and older. However, the methodological quality based on COSMIN RoB was rated as doubtful for both measurement properties. The measurement error received an indeterminate (?) rating while responsiveness achieved a sufficient (+) rating.

Both the measurement error and responsiveness evidence were very low quality owing to methodological concerns and moderate sample size (*n* = 90). The measurement error received an indeterminate (?) rating, while responsiveness was considered sufficient (+), particularly for children under 2 years of age (Table 4).

### GMFM use patterns across paediatric conditions other than cerebral palsy

There were 195 studies other than those on measurement properties (Table S10). Table 5 presents a comprehensive overview of these studies, categorized by paediatric condition. The studies covered various paediatric conditions, which were grouped into seven primary categories: central nervous system disorders (both congenital and acquired/progressive), neuromuscular disorders, genetic and chromosomal disorders, developmental disorders, metabolic/degenerative disorders, infectious disease‐related conditions, and neoplastic diseases with post‐treatment complications. The five most frequently studied conditions were Down syndrome (22 studies, 11.3%), acquired brain injury, including traumatic brain injury and brain injury (14 studies, 7.2%), Zika syndrome (11 studies, 5.6%), spinal muscular atrophy (10 studies, 5.1%), and Pompe disease (10 studies, 5.1%). Sample sizes varied widely, with 75 studies (38.5%) including 1 to 10 participants and 74 studies (37.9%) including 11 to 50 participants. Only 13 studies (6.7%) included more than 100 participants.

**TABLE 5:** Characteristics and distribution patterns of GMFM research studies.

**Temporal distribution of studies**	
Time period	*n* (%)
2020–2023 (3 April)	77 (39.5)
2015–2019	53 (27.2)
2010–2014	41 (21.0)
2005–2009	11 (5.6)
2000–2004	10 (5.1)
1998–1999	3 (1.5)
**Distribution of study designs**	
Category	*n* (%)
Case reports/series	46 (23.6)
Cross‐sectional studies	34 (17.4)
Within‐subject studies	28 (14.4)
Prospective studies	23 (11.8)
Retrospective studies	17 (8.7)
Randomized controlled trials	13 (6.7)
Clinical trials	12 (6.2)
Other/mixed designs	11 (5.6)
Cohort studies	8 (4.1)
Observational studies	3 (1.5)
**Geographical distribution of studies**	
Country	*n* (%)
USA	51 (26.2)
Brazil	20 (10.3)
Italy	18 (9.2)
Turkey	13 (6.7)
the Netherlands	13 (6.7)
South Korea	10 (5.1)
Canada	9 (4.6)
Germany	8 (4.1)
India	7 (3.6)
Japan	6 (3.1)
China	6 (3.1)
Taiwan	5 (2.6)
Denmark	4 (2.1)
Israel	3 (1.5)
UK	3 (1.5)
Australia	3 (1.5)
Others	15 (7.7)
NA	1 (0.5)
**Sample size distribution analysis**	
Sample size range	*n* (%)
Range 1–10	75 (38.5)
Range 11–50	74 (37.9)
Range 51–100	33 (16.9)
Range > 100	13 (6.7)
**Distribution of GMFM assessment (top 5)**	
Category	*n* (%)
GMFM‐88 total	122 (62.6)
GMFM dimensions	94 (48.2)
GMFM‐66	21 (10.7)
GMFM‐88	15 (7.7)
GMFM	9 (4.6)
**Most frequently used GMFM dimensions (top 5)**	
Dimension	*n* (%)
GMFM‐88 A–E	42 (21.5)
GMFM‐88 D–E	23 (11.8)
GMFM‐88 A–B	7 (3.6)
GMFM‐88 E	6 (3.1)
GMFM‐88 B	5 (2.6)
**GMFM validity descriptions**	
Category	*n* (%)
NA (no validity description)	97 (49.7)
Describing measurement properties other than CP	63 (32.3)
Describing validity in CP	24 (12.3)
Acknowledging lack of validation despite widespread use	7 (3.6)
**GMFM citation analysis**	
Citation	*n* (%)
Russell et al.^1^ (original GMFM)	31 (15.9)
Russell et al.^25^	25 (12.8)
Linder‐Lucht et al.^29^	19 (9.7)
Nelson et al.^35^	12 (6.2)
Ruck‐Gibis et al.^36^	6 (3.1)
Iannaccone et al. (2003)^33^	6 (3.1)
Iannaccone et al. (2002)^32^	4 (2.1)
Sato et al.^22^	3 (1.5)
**Disease categories in GMFM research (top 5)**	
Disease category	*n* (%)
Down syndrome	22 (11.3)
Acquired brain injury (including traumatic brain injury, brain injury)	14 (7.2)
Zika syndrome	11 (5.6)
Spinal muscular atrophy	10 (5.1)
Pompe disease	10 (5.1)
**GMFM applications by purpose**	
Purpose category	*n* (%)
Treatment evaluation	147 (75.4)
Natural history and disease progression	28 (14.4)
Clinical characterization	20 (10.3)

Abbreviations: CP, cerebral palsy; GMFM, Gross Motor Function Measure; NA, not available.

Most studies were published recently, with 77 studies (39.5%) published between 2020 and 2023. Earlier periods had fewer studies, with only three (1.5%) published between 1998 and 1999. Case reports and case series were the most common study design (46 studies, 23.6%), followed by cross‐sectional studies (34 studies, 17.4%).

The GMFM‐88 total score was used most frequently (122 studies, 62.6%). Many studies used specific GMFM dimensions (94 studies, 48.2%). Although the GMFM‐66 and GMFM‐66 item set were originally developed for CP,^3^, ^4^ they were used in 21 studies (10.7%).^27^, ^38^, ^39^, ^40^, ^41^, ^42^, ^43^, ^44^, ^45^, ^46^, ^47^, ^48^, ^49^, ^50^, ^51^, ^52^, ^53^, ^54^, ^55^, ^56^, ^57^ The most common dimension combination was GMFM‐88 A to E (42 studies, 21.5%), followed by GMFM‐88 D and E (23 studies, 11.8%). Nearly half of the studies (97 studies, 49.7%) did not describe the validity or rationale for using and adapting the GMFM, while 63 studies (32.3%) described measurement properties for conditions other than CP. Most studies reported total percentage scores; however, some did not specify the units used,^27^, ^54^, ^58^, ^59^, ^60^, ^61^, ^62^, ^63^, ^64^, ^65^, ^66^, ^67^, ^68^, ^69^, ^70^, ^71^, ^72^, ^73^ making it unclear whether they were raw scores or percentage scores. Additionally, several studies did not clearly specify which GMFM version (GMFM‐88 or GMFM‐66) was used in their methodology (Tables 5 and S10).^74^, ^75^, ^76^, ^77^, ^78^, ^79^, ^80^, ^81^, ^82^ Studies examining measurement properties also showed missing information about units^32^ and GMFM versions.^28^, ^34^

## DISCUSSION

This systematic review provides the first comprehensive analysis of GMFM measurement properties and application in children with conditions other than CP. The results show that GMFM measurement properties have been examined across eight conditions, and its applicability has been verified in various paediatric conditions. This widespread clinical use has shown an increasing trend in recent years, indicating that GMFM is recognized as an important tool for assessing gross motor function in children.

The first objective revealed measurement properties across eight conditions, but highlighted notable concerns. The systematic review of GMFM measurement properties showed that most studies met the criteria for good measurement properties and received sufficient (+) ratings across various paediatric conditions, despite being rated as ‘doubtful’ in terms of methodological quality. Furthermore, because of serious RoB and imprecision (total sample sizes < 50), the quality of evidence for measurement properties was generally low or very low according to modified GRADE criteria.

An important finding from these results is that RoB assessment and quality evaluation, or modified GRADE and overall rating, can show different results because these two assessments examine different aspects of study quality. Studies with poor methodology may still receive sufficient (+) ratings for measurement properties when their results meet the required statistical criteria or hypotheses. Future GMFM measurement property research requires improved study designs and methodological approaches.

Content validity is the most important measurement property that forms the foundation of measurement tools and is positioned as a prerequisite for all other measurement properties in COSMIN guidelines.^17^ However, content validity verification was conducted in only one study targeting ALL.^26^ This finding demonstrates problems in two important aspects. First, among the eight conditions where measurement properties were examined, content validity was verified only for ALL (GMFM‐ALL), while content validity verification was not conducted for the other seven conditions. Second, considering that four modified GMFM versions^24^, ^25^, ^26^, ^27^ adapted for specific conditions were identified, only GMFM‐ALL received formal content validity verification among these, while systematic expert evaluation was not conducted for the modified versions of GMFM‐DS,^25^ GMFM‐FCMD,^24^ and GMFM in infancy^27^ applications. This widespread lack of content validity verification raises important questions about the appropriateness of GMFM items for different conditions. While the modified versions incorporate condition‐specific modifications to scoring systems and item selection, systematic verification based on COSMIN guidelines is lacking. Since content validity forms the foundation for all other measurement properties, this verification deficit represents a particularly serious challenge.

Important findings were also obtained about the second objective of GMFM's change detection capability in conditions other than CP. Responsiveness varied across conditions, with acquired brain injury^1^, ^28^, ^29^, ^31^ and Pompe disease^37^ showing sufficient change detection capability, while Down syndrome^25^ and ALL^26^ showed inconsistent results. These differences between conditions may reflect the relationship between GMFM and each condition's disease progression patterns and treatment responsiveness, in addition to differences in study design.

The results show that age and severity can be important determinants of change detection capability. Pompe disease showed higher effect sizes in children under 2 years of age,^37^ and Down syndrome also showed greater improvement in younger children with mild impairments.^25^ ALL also showed changes in dimension D for younger children (under 5 years) compared with older children, but no significant changes were observed in dimension E.^26^ These results suggest the high plasticity of motor function in early childhood, while indicating that certain GMFM dimensions may not detect changes depending on the condition. This suggests the importance of conducting measurement property studies for each condition.

Regarding the second objective of change detection capability as an outcome measure, while most studies (75.4%) used GMFM for treatment evaluation, interpretation of change detection capability is complicated by insufficient measurement property studies. For the eight conditions with identified measurement properties, some evidence for change detection capability exists as discussed above. However, for conditions beyond these eight, change detection capability remains unclear because of lack of validation studies. This suggests that many treatment evaluation studies may be using an outcome measure without established sensitivity to change for their specific population. This emphasizes the need for condition‐specific measurement property studies to ensure valid interpretation of intervention outcomes.

The third objective of GMFM use purposes showed that more than 70% were primarily for treatment evaluation, suggesting interest in whether gross motor functions change through interventions or longitudinal studies. However, this finding was confirmed even in conditions where the aforementioned measurement properties have not been verified, highlighting an important gap between research validation and clinical practice.

Clarifying the fourth objective of why GMFM is used for gross motor function assessment also suggests an important gap between research validation and clinical practice. While studies targeting the eight conditions with identified measurement properties frequently referenced those condition‐specific measurement properties, for conditions other than these eight, cases were observed where the reason for applying GMFM was not documented, CP measurement properties were cited, or applications in the eight conditions other than CP were mentioned. This suggests that many clinicians and researchers have insufficient understanding of GMFM measurement properties or use it despite understanding that measurement properties are insufficient.

The GMFM was developed for CP and consists of items covering gross motor functions reached by typically developing children by age 5 years. Since many children with motor impairments are estimated not to have acquired the gross motor abilities that typically developing children reach by age 5 years, researchers and clinicians probably use this GMFM because it enables developmentally appropriate assessment. Furthermore, we found that researchers sometimes use only one or multiple GMFM dimensions rather than the complete GMFM‐88 total score. This selective use may reflect researchers’ attempts to address content validity concerns by focusing on motor areas most relevant to or targeted for specific diagnoses.

The quality of GMFM research is often compromised by insufficient methodological reporting. Specifically, many studies do not adequately describe the GMFM type, assessment dimensions, or GMFM‐88 units. This lack of basic information prevents clinicians from making meaningful comparisons of gross motor function changes across studies. The lack of consistency in reporting GMFM assessment details not only affects result interpretation but also limits the potential for future meta‐analyses, emphasizing the critical need for standardized reporting protocols. For modified versions of GMFM‐88, researchers should detail any changes to items or scoring methods, following the example presented in the clinical utility of GMFM in this study. GMFM‐66 was originally developed for children with CP^1^, ^3^ and has been validated for acquired brain injury.^29^ Our findings showed that it has been used for various other diagnostic groups. However, when applying GMFM‐66 to these other groups, clinicians and researchers should interpret results with caution. To ensure research quality and comparability, future studies should include three key elements: (1) GMFM version; (2) assessed dimensions; and (3) scoring methods and units.

The comprehensive analysis of 210 studies identified in this study revealed important challenges for GMFM use in conditions other than CP. Results from both the 15 measurement property studies and 195 clinical application studies revealed that measurement properties have not been sufficiently verified in many conditions where GMFM is currently widely used. Therefore, researchers and clinicians should interpret results cautiously when using GMFM in populations other than CP.

On the basis of these findings, future research should focus on the following points. First, content validity studies should be verified for each diagnostic group where GMFM is applied, including formal evaluation of existing modified versions. This work should include establishing expert panels comprising clinicians familiar with condition‐specific motor challenges, systematic review of GMFM items for relevance and appropriateness, and potential modification of items or rating scales when necessary.

Second, improvement in methodological quality of measurement property studies and establishment of clear GMFM reporting guidelines are needed. This includes standardized documentation of GMFM versions, assessed dimensions, and scoring methods to facilitate interpretation and comparison of results across studies.

From a clinical perspective, since validity, reliability, and responsiveness differ across conditions, condition‐specific verification of measurement properties is necessary when applying GMFM to conditions other than CP. Additionally, because measurement properties may be influenced by age and severity, conducting measurement property studies with clearly defined target population characteristics is important. Until such validity verification work is completed, clinicians and researchers should interpret results cautiously when using GMFM in populations other than CP. In particular, considering the current widespread use of GMFM across diverse diagnostic groups other than CP and the existence of modified versions without formal content validity verification, verification of content validity in each diagnostic group represents an urgent research priority.

This study has several important limitations. First, the small number of high‐quality studies and limited sample sizes precluded meta‐analysis, restricting our ability to establish robust evidence for measurement properties. Most of the studies included had small sample sizes (*n* < 50), and the methodological quality varied considerably from inadequate to very good according to the COSMIN criteria. Furthermore, studies used different statistical methods to assess the same measurement properties, making quantitative synthesis impossible. The identified methodological issues, particularly in sample size and statistical analysis, reflect common challenges in paediatric assessment tool validation. This suggests the need for collaborative multi‐centre studies to overcome these limitations.

The second limitation relates to our search comprehensiveness. Two additional studies^38^, ^83^ evaluating GMFM in children with hereditary spastic paraplegia were identified from the authors’ previous research. While these papers were not captured by our current search terms, we believe our search strategy was appropriate given the volume of literature identified. However, the existence of these uncaptured papers suggests that GMFM may be used in a broader range of conditions than that identified by our review.

Third, the evolving definition of CP may affect the classification of some conditions included in our review. The recently updated description by Dan et al. expands the conceptualization of CP to include genetic causes and early brain dysplasia or injury occurring in the first 2 to 3 years of life.^84^ This broader understanding may lead to the reclassification of some conditions previously considered as conditions other than CP, particularly those involving early‐onset brain injury or genetic factors affecting brain development. While our study was conducted on the basis of the diagnostic criteria available at the time of data collection (April 2023), future research may need to reconsider the classification of certain conditions in light of this evolving understanding of CP.

## CONCLUSION

The GMFM measurement properties for children with conditions other than CP are supported by low‐ to very‐low‐quality evidence across eight conditions. Clinicians and researchers should interpret GMFM results cautiously when assessing children with conditions outside these eight validated diagnostic groups. Future research should prioritize content validity verification for each condition where GMFM is applied, improve methodological quality through appropriate sample sizes, and develop standardized reporting guidelines specifying GMFM version and scoring methods.

## FUNDING INFORMATION

JSPS KAKENHI grant number JP 21K11315.

## CONFLICT OF INTEREST STATEMENT

The authors have stated that they had no interests that might be perceived as posing a conflict or bias.

## Supporting information


**Figure S1:** Flow diagram of the article selection process according to the PRISMA guidelines

**Table S1:** Review Prompts for Systematic AI‐Assisted Literature Screening of Non‐English and Non‐Japanese Publications

**Table S2:** Measurement properties of the Gross Motor Function Measure in children with acquired brain injury

**Table S3:** Measurement properties of the Gross Motor Function Measure in children with spinal muscular atrophy

**Table S4:** Measurement properties of the Gross Motor Function Measure in children with Fukuyama congenital muscular dystrophy

**Table S5:** Measurement properties of the Gross Motor Function Measure in children with Down syndrome

**Table S6:** Measurement properties of the Gross Motor Function Measure in children with osteogenesis imperfecta

**Table S7:** Measurement properties of the Gross Motor Function Measure in children with acute lymphoblastic leukemia

**Table S8:** Measurement properties of the Gross Motor Function Measure in children with leukodystrophy

**Table S9:** Measurement properties of the Gross Motor Function Measure in children with Pompe disease

**Table S10:** Non‐measurement studies using the Gross Motor Function Measure in children with conditions other than cerebral palsy

## Data Availability

The data that support the findings of this study are available from the corresponding author upon reasonable request.
