# Supplementary figures and images for: Application of the Gross Motor Function Measure in children with conditions other than cerebral palsy: A systematic review
Source: Dev Med Child Neurol. 2025 Aug 14;67(11):1421–42. doi: 10.1111/dmcn.16465 (PMC12521613; doi:10.1111/dmcn.16465)

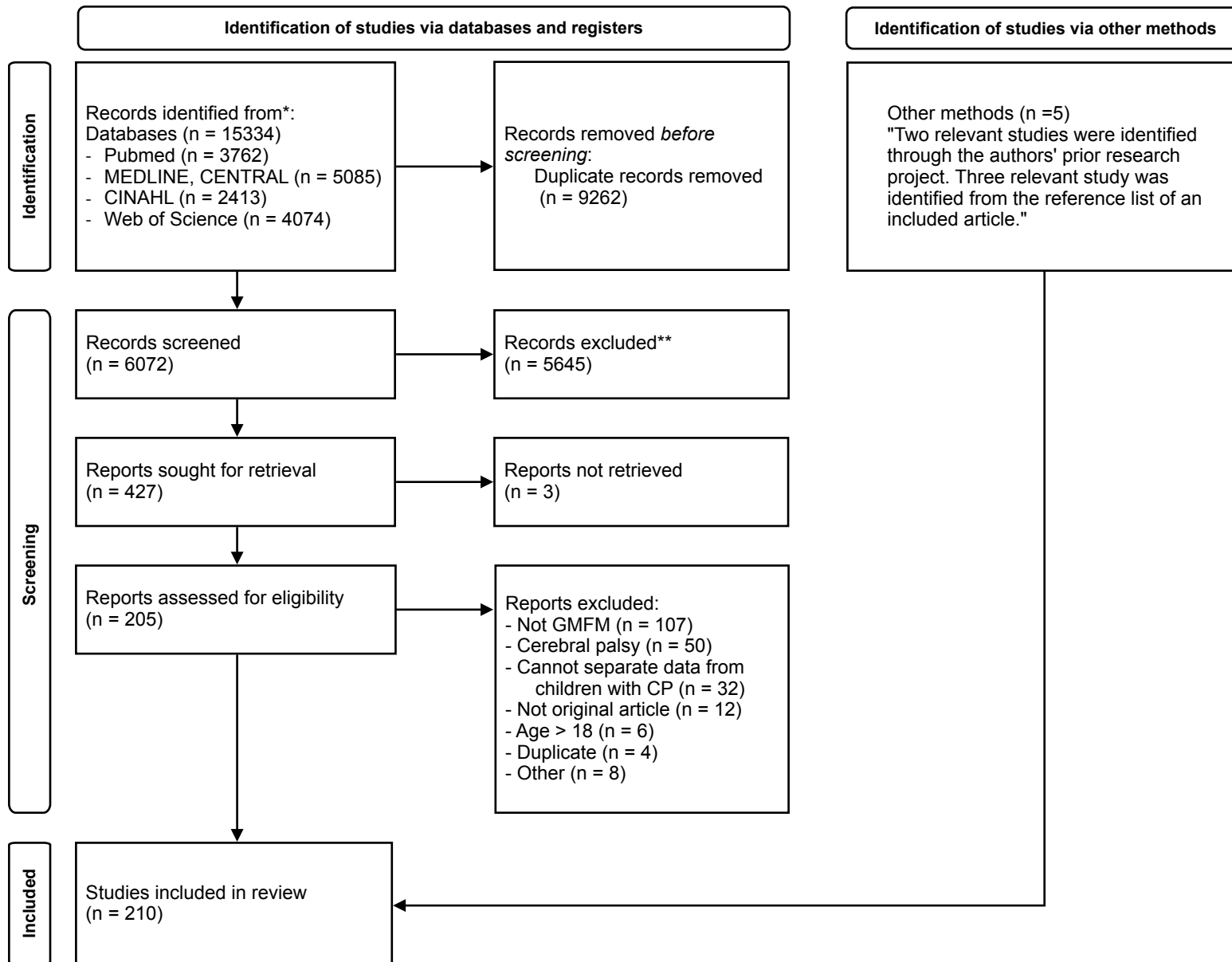

Figure S1. Flow diagram of the article selection process according to the PRISMA guidelines

Supplement: Supplementary file 1 — Figure S1: Flow diagram of the article selection process according to the PRISMA guidelines [file DMCN-67-1421-s005.pdf]
