# Supplementary material for: Application of the Gross Motor Function Measure in children with conditions other than cerebral palsy: A systematic review
Source: Dev Med Child Neurol. 2025 Aug 14;67(11):1421–42. doi: 10.1111/dmcn.16465 (PMC12521613; doi:10.1111/dmcn.16465)
Supplement: Supplementary file 2 — Table S1: Review Prompts for Systematic AI‐Assisted Literature Screening of Non‐English and Non‐Japanese Publications [file DMCN-67-1421-s009.docx]

| Table S1. Review Prompts for Systematic AI-Assisted Literature Screening of Non-English and Non-Japanese Publications |
| --- |
| Please analyze the attached paper and provide information according to the following criteria:  1. Patient Demographics:     - Identify the primary diagnosis/disorder     - Specify age range of participants  2. GMFM Assessment:     - Confirm if GMFM was used as an outcome measure     - If used, specify which version of GMFM  3. If the study meets these criteria:     - Includes patients aged 0-18 years     - Diagnoses other than cerebral palsy     - Uses GMFM as an outcome measure     Please extract and organize the following data in a table format:     - Year of publication     - Country of study     - Study design     - Diagnosis/Disorder     - Total sample size     - GMFM sample size     - Mean age (SD) and range     - Type of GMFM used     - Purpose of GMFM assessment     - Sex distribution (female/male) |
