# Supplementary material for: Application of the Gross Motor Function Measure in children with conditions other than cerebral palsy: A systematic review
Source: Dev Med Child Neurol. 2025 Aug 14;67(11):1421–42. doi: 10.1111/dmcn.16465 (PMC12521613; doi:10.1111/dmcn.16465)
Supplement: Supplementary file 3 — Table S2: Measurement properties of the Gross Motor Function Measure in children with acquired brain injury [file DMCN-67-1421-s002.docx]

Table S2. Measurement properties of the Gross Motor Function Measure in children with acquired brain injury

| Study characteristics and measurement property findings for the Gross Motor Function Measure in children with acquired brain injury | | | | | | | | | | | | |
| --- | --- | --- | --- | --- | --- | --- | --- | --- | --- | --- | --- | --- |
| **Study** | **Year** | **Country** | **Diagnosis** | **N** | **Mean age (SD); range** | **Time from Injury to Assessment** | **Type of GMFM** | **Measurement Property Evaluated** | **n** | **Results** | **COSMIN**  **BOX** |  |
| Russell et al.^1^ | 1989 | Canada | Head Injury (HI) | 25 ^a^ | NA | Acute/recovery phase | Original GMFM  (GMFM-88) | Responsiveness  (Comparison with other groups) | 24 | There was a significant difference between change scores for the HI and CP groups. | 10c |  |
| Thomas-Stonell et al.^28^ | 2006 | Canada | Traumatic brain injuries (TBI) | 27 ^a^ | 12.5 (4.5); 4–18 years ^b^ | 13±6 months  (range 2–23 months) | GMFM^b^ | Responsiveness  (Comparison before and after Neurorehabilitation Programme) | 27 | Standardized Response Mean = 0.62 | 10d |  |
| Linder-Lucht et al.^29^ | 2007 | Germany | TBI | 73 | 11.4 (5.1); 0.8–18.9 years | 0.86±2.0 years  (range 0.0-11.4 years) | GMFM-88 Total (raw)  GMFM-66 | Test-retest reliability  (GMFM-66 & GMFM-88) | 10 | ICC = 0.99 | 6 |  |
|  |  |  |  |  |  |  |  | Responsiveness  (Comparison with judgement by person) | 70 | GMFM-88  r = 0.657 and 0.737 (video assessor)  r = 0.555 (physiotherapists)  r = 0.531 (parents)  GMFM-66  r = 0.679 (video assessor 1)  r = 0.536 (video assessor 2)  r = 0.609 (physiotherapists)  r = 0.563 (parents) | 10b |  |
|  |  |  |  |  |  |  |  | Responsiveness  (Comparison by duration of injury) | 73 | Changes in gross motor function became fewer as the interval between brain injury and baseline increased. | 10c |  |
| Storm et al.^31^ | 2020 | Italy | Acquired brain injury | 110 | 10.8 (4.1) years | 1.7±1.9 years | GMFM-88 D-E (%)  GMFM-88 Total (%) | Responsiveness  (Comparison before and after Robot-assisted gait training) | 110 | GMFM-88 (%): MCID = 1.1–5.3 (overall)  Dimension D (%): MCID = 2.3–6.1 (overall)  Dimension E (%): MCID = 2.8–6.5 (overall) | 10d |  |
| Abbreviations: COSMIN, COnsensus-based Standards for the selection of health Measurement INstruments; CP, Cerebral Palsy; GMFM, Gross Motor Function Measure; HI, Head Injury; ICC, Intraclass Correlation Coefficient; MCID, Minimal Clinically Important Difference; N, total number of participants; n, number of participants in specific analysis; NA, not available; r, correlation coefficient; SD, standard deviation; TBI, Traumatic Brain Injury.  ^a^ N for GMFM assessment  ^b^ The original text was retained and data were combined under GMFM-88 | | | | | | | | | | | |  |

Risk of bias and quality assessment for reliability of the Gross Motor Function Measure in children with acquired brain injury

| Risk of Bias and reliability assessment | | | |
| --- | --- | --- | --- |
| ***Box 6. Reliability*** | | Linder-Lucht et al. | |
|  |  | Test-retest reliability (GMFM-88 & GMFM-66) | |
|  |  | Consensus | Rating Justification |
| 1 | Were patients stable in the time between the repeated measurements on the construct to be measured? | VG | The measurement interval was 3.8 days on average, and patient condition was judged to be stable. |
| 2 | Was the time interval between the measurements appropriate? | D | The measurement interval of 3.8 days on average is short. |
| 3 | Were the measurement conditions similar for the measurements – except for the condition being evaluated as a source of variation? | D | It is unclear whether habituation to measurement was considered. |
| 4 | Did the professional(s) administer the measurement without knowledge of scores or values of other repeated measurement(s) in the same patients? | D | No mention of blinding. |
| 5 | 5. Did the professional(s) assign scores or determine values without knowledge of the scores or values of other repeated measurement(s) in the same patients? | D | No mention of blinding. |
| 6 | Were there any other important flaws in the design or statistical methods of the study? | VG | No major defects. |
| 7 | For continuous scores: was an intraclass correlation coefficient (ICC) calculated? | A | No description of ICC model or formula. |
| 8 | For ordinal scores: was a (weighted) kappa calculated? | NA |  |
| 9 | For dichotomous/nominal scores: was Kappa calculated for each category against the other categories combined? | NA |  |
|  | **QUALITY OF THE STUDY** *Lowest score of standards 1-7* | **D** |  |
| **Rating** | | **＋** | ICC ≥ 0.70 |

| GRADE evaluation of reliability study (GMFM-88 & GMFM-66) | | |
| --- | --- | --- |
| Item | Judge | Justification |
| Risk of bias | −2: Very serious | One study of doubtful quality only. |
| Inconsistency | Non | Only one study |
| Imprecision | −2: total n<50 | Total sample size=10 |
| Indirectness | Non | Only one study |
| **GRADE** | **Very Low** | −4 grade down |
| **Rating** | **＋** | The results were deemed sufficient as more than 75% of them met the criteria. |

Abbreviations: A, adequate; D, doubtful; GMFM, Gross Motor Function Measure; GRADE, Grading of Recommendations Assessment, Development and Evaluation; ICC, Intraclass Correlation Coefficient; n, number of participants; NA, not available; VG, very good; +, sufficient rating.

Risk of bias and quality assessment for responsiveness of the Gross Motor Function Measure in children with acquired brain injury

| Risk of Bias and responsiveness assessment | | | | | | | |
| --- | --- | --- | --- | --- | --- | --- | --- |
| ***Box 10. Responsiveness*** | | Linder-Lucht et al. | |  | | | |
| **10b. Construct approach (comparison with other outcome measurement instruments)** | | Comparison with judgement by person (GMFM-88 & GMFM-66) | |  | |  | |
|  |  | Consensus | Rating Justification |  |  | |  |
| 4 | Is it clear what the comparator instrument(s) measure(s)? | VG | The purpose of evaluating gross motor function is clearly defined. |  |  | |  |
| 5 | Were the measurement properties of the comparator instrument(s) sufficient? | A | Because the same method as previous studies was used. |  |  | |  |
| 6 | Were design and statistical methods adequate for the comparisons being made? | VG | Hypothesis was present and tested. |  |  | |  |
| 7 | Were there any other important flaws? | VG | No major defects. |  |  | |  |
|  | **QUALITY OF THE STUDY** *Lowest score of standards 4-7* | **A** |  |  |  | |  |
| **Rating** | | **＋** | Conformed to the original authors' hypothesis. |  |  | |  |
|  |  | Russell et al. | | Linder-Lucht et al. | | | |
|  |  | Comparison with other groups (GMFM-88) | | Comparison by duration of injury (GMFM-88 & GMFM-66) | | | |
| **10c. Construct approach: (comparison between subgroups)** | | Consensus | Rating Justification | Consensus | Rating Justification | |  |
| 8 | Was an adequate description provided of important characteristics of the subgroups? | D | Limited description of group characteristics. | D | Limited description of group characteristics. | |  |
| 9 | Were design and statistical methods adequate for the subgroups being compared? | A | Hypothesis was present and tested. | A | Hypothesis was present and tested. | |  |
| 10 | Were there any other important flaws? | VG | No major defects. | VG | No major defects. | |  |
|  | **QUALITY OF THE STUDY** *Lowest score of standards 8-10* | **D** |  | **D** |  | |  |
| **Rating** | | **＋** | Conformed to the original authors' hypothesis. | **＋** | Conformed to the original authors' hypothesis. | |  |
|  |  | Thomas-Stonell et al. | | Storm et al. | | | |
|  |  | Comparison before and after Neurorehabilitation Programme  (GMFM-88) | | Comparison before and after Robot-assisted gait training  (GMFM-88) | | | |
| **10d. Construct approach: (comparison before and after intervention)** | | Consensus | Rating Justification | Consensus | Rating Justification | |  |
| 11 | Was an adequate description provided of the intervention given? | D | Details of intervention are not described. | VG | Details of intervention are described | |  |
| 12 | Was the statistical method appropriate for the before-after comparison being made? | A | Calculated using Standardized Response Mean (SRM). | A | Calculated minimum clinically important difference (MCID). | |  |
| 13 | Were there any other important flaws? | VG | No major defects. | VG | No major defects. | |  |
|  | **QUALITY OF THE STUDY** *Lowest score of standards 11-13* | **D** |  | **A** |  | |  |
| **Rating** | | **＋** | The review team hypothesized moderate or higher values (0.5 or above). | **＋** | The review team hypothesized that MCID would be higher in the ABI group than in the CP group. | |  |

| GRADE evaluation of responsiveness studies (GMFM-88) | | |  | GRADE evaluation of responsiveness study (GMFM-66) | | |
| --- | --- | --- | --- | --- | --- | --- |
| Item | Judge | Justification |  | Item | Judge | Justification |
| Risk of bias | −1: Serious | Multiple studies of at least adequate quality are available, but multiple studies of doubtful quality exist. |  | Risk of bias | −1: Serious | One study of adequate quality is available. |
| Inconsistency | Non | The research findings are consistent. |  | Inconsistency | Non | Only one study (Linder-Lucht et al.) |
| Imprecision | Non | Total sample size=234 |  | Imprecision | −1: total n=50-100 | Total sample size=73 |
| Indirectness | −1: Serious | Integrated analysis of TBI, ABI and head injury. |  | Indirectness | Non | Study population directly matched the review question. |
| **GRADE** | **Low** | −2 grade down |  | **GRADE** | **Low** | −1 grade down |
| **Rating** | **＋** | The results were deemed sufficient as more than 75% of them met the criteria |  | **Rating** | **＋** | The results were deemed sufficient as more than 75% of them met the criteria |

Abbreviations: A, adequate; ABI, Acquired Brain Injury; CP, Cerebral Palsy; D, doubtful; GMFM, Gross Motor Function Measure; GRADE, Grading of Recommendations Assessment, Development and Evaluation; MCID, Minimal Clinically Important Difference; n, number of participants; SRM, Standardized Response Mean; TBI, Traumatic Brain Injury; VG, very good; +, sufficient rating.
