# Supplementary material for: Application of the Gross Motor Function Measure in children with conditions other than cerebral palsy: A systematic review
Source: Dev Med Child Neurol. 2025 Aug 14;67(11):1421–42. doi: 10.1111/dmcn.16465 (PMC12521613; doi:10.1111/dmcn.16465)
Supplement: Supplementary file 4 — Table S3: Measurement properties of the Gross Motor Function Measure in children with spinal muscular atrophy [file DMCN-67-1421-s007.docx]

Table S3. Measurement properties of the Gross Motor Function Measure in children with spinal muscular atrophy

| Study characteristics and measurement property findings for the Gross Motor Function Measure in children with spinal muscular atrophy | | | | | | | | | | | |
| --- | --- | --- | --- | --- | --- | --- | --- | --- | --- | --- | --- |
| **Study** | **Year** | **Country** | **Diagnosis** | **N** | **Mean age (SD); range** | **SMA Type / Ambulatory Status** | **Type of GMFM** | **Measurement Property Evaluated** | **n** | **Results** | **COSMIN**  **BOX** |
| Iannaccone et al.^32^ | 2002 | United States | Spinal muscular atrophy (SMA) | 10 | 7.4; 2–14 years | Not reported; Walkers (1), Non-walkers (9) | GMFM-88 A-B  GMFM-88 Total | Inter-rater reliability  (GMFM-88 A-B) | 10 | κ = 0.72 | 6 |
|  |  |  |  |  |  |  |  | Inter-rater reliability  (GMFM-88 Total) | 10 | In the Friedman test for repeated measurements, there were no significant differences between the total GMFM. | 6 |
| Iannaccone et al.^33^ | 2003 | United States | SMA | 34 | 2–17 years | Not reported; Walkers (12), Non-walkers (22) | GMFM-88 A-E (raw & %)  GMFM-88 Total (raw & %) | Intra-rater reliability | 34 | ICC = 0.96–0.98 (Total & A-E) | 6 |
| Nelson et al.^35^ | 2006 | United States | SMA | 40 | 10.90 (3.57) years | Not reported; Walkers (13), Non-walkers (27) | GMFM-88 A-E (raw)  GMFM-88 Total (raw) | Construct validity  (Comparison with Quantitative Muscle Testing) | 40 | r = 0.84 (Total)  r = 0.77–0.86 (A-E) | 9a |
|  |  |  |  |  |  |  |  | Construct validity  (Comparison between ambulatory status) | 40 | The walkers were significantly higher than the non-walkers. | 9b |
|  |  |  |  |  |  |  |  | Construct validity  (Comparison between BiPAP ventilation status) | 40 | The BiPAP group was significantly lower than the non-BiPAP group. | 9b |
| Chen et al.^34^ | 2014 | Taiwan | SMA | 38 | 8–31 years^a^ | Type II (7), Type III (3); All nonwalking | GMFM^d^ | Intra-rater reliability at home | 10 | ICC = 0.9938 | 6 |
|  |  |  |  |  |  |  |  | Intra-rater reliability in hospital | 28 | ICC = 0.9968 | 6 |
|  |  |  |  |  |  |  |  | Construct validity  (Comparison with Modified Hammersmith Functional Motor Scale) | 38 | r = 0.95 | 9a |
|  |  |  |  |  |  |  |  | Construct validity (difference of in-hospital vs at-home) | 38 | There is a significant difference between the home and the hospital. | 9b |
|  |  |  |  |  |  |  |  | Construct validity (Calculated intrasubject differences) | 3 | There is a significant difference between the home and the hospital. | 9b |
| Abbreviations: BiPAP, Bi-level Positive Airway Pressure; COSMIN, COnsensus-based Standards for the selection of health Measurement INstruments; GMFM, Gross Motor Function Measure; ICC, Intraclass Correlation Coefficient; κ, kappa coefficient; N, total number of participants; n, number of participants in specific analysis; r, Pearson correlation coefficient; SD, standard deviation; SMA, Spinal Muscular Atrophy.  ^a^ Age of subset participants | | | | | | | | | | | |

Risk of bias and quality assessment for reliability of the Gross Motor Function Measure in children with spinal muscular atrophy

| Risk of Bias and reliability assessment | | | | | | | | | |
| --- | --- | --- | --- | --- | --- | --- | --- | --- | --- |
| ***Box 6. Reliability*** | | Iannaccone et al. 2002 | | Iannaccone et al. 2002 | | Iannaccone et al. 2003 | | Chen et al.^a^ | |
|  |  | Inter-rater reliability (GMGM-88 A-B) | | Inter-rater reliability (GMFM-88 Total) | | Intra-rater reliability  (GMGM-88 A-E & GMFM-88 Total) | | Intra-rater at-home &  Intra-rater in-hospital | |
|  |  | Consensus | Rating Justification | Consensus | Rating Justification | Consensus | Rating Justification | Consensus | Rating Justification |
| 1 | Were patients stable in the time between the repeated measurements on the construct to be measured? | NA |  | NA |  | VG | The procedure was conducted on the assumption that the condition was stable. | I | Children may be unstable due to being part of an RCT study. |
| 2 | Was the time interval between the measurements appropriate? | D | There is insufficient description of measurement intervals. | D | There is insufficient description of measurement intervals. | VG | The measurement period of 4 weeks is appropriate. | I | Time intervals are not specified. |
| 3 | Were the measurement conditions similar for the measurements – except for the condition being evaluated as a source of variation? | D | It is unclear whether habituation to measurement was considered. | D | It is unclear whether habituation to measurement was considered. | VG | There is a clear statement that learning sessions were provided. | D | It is unclear whether habituation to measurement was considered. |
| 4 | Did the professional(s) administer the measurement without knowledge of scores or values of other repeated measurement(s) in the same patients? | A | Other assessments such as muscle strength measurements were also conducted, but the measurement procedures followed the same order, so the impact is considered to be low. | A | Other assessments such as muscle strength measurements were also conducted, but the measurement procedures followed the same order, so the impact is considered to be low. | A | Other assessments such as muscle strength measurements were also conducted, but the measurement procedures followed the same order, so the impact is considered to be low. | D | Assessment was performed by a single physical therapist, and details are not provided. |
| 5 | Did the professional(s) assign scores or determine values without knowledge of the scores or values of other repeated measurement(s) in the same patients? | A | Although there is no clear description, it is assumed that the data was not referenced. | A | Although there is no clear description, it is assumed that the data was not referenced. | A | Although there is no clear description, it is assumed that the data was not referenced. | D | Assessment was performed by a single physical therapist, and details are not provided. |
| 6 | Were there any other important flaws in the design or statistical methods of the study? | VG | No major defects. | VG | No major defects. | VG | No major defects. | VG | No major defects. |
| 7 | For continuous scores: was an intraclass correlation coefficient (ICC) calculated? | NA |  | NA |  | A | No description of ICC model or formula. | A | No description of ICC model or formula. |
| 8 | For ordinal scores: was a (weighted) kappa calculated? | A | Calculated using kappa coefficient. | A | Calculated using the Friedman test for repeated measurements. | NA |  | NA |  |
| 9 | For dichotomous/nominal scores: was Kappa calculated for each category against the other categories combined? | NA |  | NA |  | NA |  | NA |  |
| **QUALITY OF THE STUDY** *Lowest score of standards 1-7* | | **D** |  | **D** |  | **A** |  | **I** |  |
| **Rating** | | **＋** | Kappa ≥ 0.70 | **?** | ICC or weighted Kappa not reported | **＋** | ICC ≥ 0.70 | **＋** | ICC ≥ 0.70 |

| GRADE evaluation of reliability studies | | |
| --- | --- | --- |
| Item | Judge | Justification |
| Risk of bias | −1: Serious | There are multiple studies of doubtful quality available, or there is only one study of adequate quality. |
| Inconsistency | Non | Results are consistent across studies except for data calculated using the Friedman test for repeated measurements. |
| Imprecision | −1: total n=50-100 | Total sample size=82 |
| Indirectness | Non | The majority of study participants consisted of pediatric participants with non-ambulatory status. |
| **GRADE** | **Low** | −2 grade down |
| **Rating** | **＋** | The results were deemed sufficient as more than 75% of them met the criteria. |

Abbreviations: A, adequate; D, doubtful; GMFM, Gross Motor Function Measure; GRADE, Grading of Recommendations Assessment, Development and Evaluation; I, inadequate; ICC, Intraclass Correlation Coefficient; n, number of participants; NA, not applicable; RCT, Randomized Controlled Trial; VG, very good; +, sufficient rating; ?, indeterminate rating.

^a^ Chen et al. assessed both intra-rater at-home and intra-rater in-hospital reliability, but since both evaluations received identical Risk of Bias ratings, they are combined into a single column.

Risk of bias and quality assessment for construct validity of the Gross Motor Function Measure in children with spinal muscular atrophy

| Risk of Bias and construct validity assessment | | | | | | | | | | | | |
| --- | --- | --- | --- | --- | --- | --- | --- | --- | --- | --- | --- | --- |
| ***Box 9. Hypotheses testing for construct validity*** | | Nelson et al. | | | | Chen et al. | | | | |  |  |
| **9a. Comparison with other outcome measurement instruments (convergent validity)** | | Comparison with Quantitative Muscle Testing | | | | Comparison with Modified Hammersmith Functional Motor Scale | | | | |  |  |
|  |  | Consensus | Rating Justification | | | Consensus | | Rating Justification | | |  |  |
| 1 | Is it clear what the comparator instrument(s) measure(s)? | VG | Quantitative muscle testing is clearly described as a muscle strength assessment. | | | VG | | The Modified Hammersmith Functional Motor Scale is a motor function assessment for SMA populations. | | |  |  |
| 2 | Were the measurement properties of the comparator instrument(s) sufficient? | VG | There is a description that it has been used in this population for over two decades. | | | VG | | The reliability of the Modified Hammersmith Functional Motor Scale has already been established in previous research. | | |  |  |
| 3 | Were design and statistical methods adequate for the comparisons being made? | VG | Spearman rank order (rho) correlations were used, and the distribution of the data is also shown. | | | A | | Pearson correlation was used, but distribution characteristics and standard deviations were not reported. | | |  |  |
| 4 | Were there any other important flaws? | VG | No major defects. | | | VG | | No major defects. | | |  |  |
| **QUALITY OF THE STUDY** *Lowest score of standards 1-4* | | **VG** |  | | | **A** | |  | | |  |  |
| **Rating** | | **＋** | The review team hypothesized that the correlation coefficient would be > 0.7. Over 75% of results were > 0.7. | | | **＋** | | The review team hypothesized that the correlation coefficient would be > 0.7. | | |  |  |
|  |  | Nelson et al. | | Nelson et al. | | Chen et al. | | | Chen et al. | | |  |
| **9b. Comparison between subgroups (discriminative or known-groups validity)** | | Comparison between ambulatory status | | Comparison between BiPAP ventilation status | | Difference of in-hospital vs at-home | | | Calculated intrasubject differences | | |  |
|  |  | Consensus | Rating Justification | Consensus | Rating Justification | Consensus | Rating Justification | | Consensus | Rating Justification | |  |
| 5 | Was an adequate description provided of important characteristics of the subgroups? | A | Age and gender are clearly described. | D | Characteristics of BiPAP users versus non-users are not described. | D | Characteristics of in-hospital group are not described. | | A | Characteristics are described in Table 1. | |  |
| 6 | Were design and statistical methods adequate for the subgroups being compared? | A | There is documentation about the overlap between groups. | D | BiPAP user group with only 5 subjects is too small for appropriate statistical analysis. | A | At-home group with only 10 subjects is too small for appropriate statistical analysis. | | I | The sample size of 3 participants for intrasubject comparisons is too small. | |  |
| 7 | Were there any other important flaws? | VG | No major defects. | D | BiPAP user group consisting entirely of non-walkers suggests possible confounding factors. | D | No description of methods used in the in-hospital setting. | | I | The study focused only on non-walking participants, who represent a highly heterogeneous subgroup within the SMA population. | |  |
| **QUALITY OF THE STUDY** *Lowest score of standards 5-7* | | **A** |  | **D** |  | **D** |  | | **I** |  | |  |
| **Rating** | | **＋** | The review team hypothesized that the walkers would be significantly higher than the non-walkers. | **＋** | The review team hypothesized that the BiPAP group would be significantly lower than the non-BiPAP group. | **−** | The review team hypothesized that there would be no significant differences between in-hospital and at-home environments. | | **−** | The review team hypothesized that there would be no significant differences between in-hospital and at-home environments. | |  |

| GRADE evaluation of construct validity studies | | |
| --- | --- | --- |
| Item | Judge | Justification |
| Risk of bias | −1: Serious | There is one study of very good quality and multiple studies of adequate quality, but there is one study of inadequate quality. |
| Inconsistency | Non | The research findings are consistent. |
| Imprecision | −1: total n=50-100 | Total sample size=78 |
| Indirectness | Non | Most of the participants were children with SMA. |
| **GRADE** | **Low** | −2 grade down |
| **Rating** | **＋** | While the agreement of research findings falls below the 75% threshold, with very good or adequate quality methodology, the research results are consistent. |

Abbreviations: A, adequate; BiPAP, Bi-level Positive Airway Pressure; D, doubtful; GMFM, Gross Motor Function Measure; GRADE, Grading of Recommendations Assessment, Development and Evaluation; I, inadequate; n, number of participants; rho, Spearman's rank correlation coefficient; SMA, Spinal Muscular Atrophy; VG, very good; +, sufficient rating; -, insufficient rating.
