# Supplementary material for: Application of the Gross Motor Function Measure in children with conditions other than cerebral palsy: A systematic review
Source: Dev Med Child Neurol. 2025 Aug 14;67(11):1421–42. doi: 10.1111/dmcn.16465 (PMC12521613; doi:10.1111/dmcn.16465)
Supplement: Supplementary file 5 — Table S4: Measurement properties of the Gross Motor Function Measure in children with Fukuyama congenital muscular dystrophy [file DMCN-67-1421-s001.docx]

Table S4. Measurement properties of the Gross Motor Function Measure in children with Fukuyama congenital muscular dystrophy

| Study characteristics and measurement property findings for the Gross Motor Function Measure in children with Fukuyama congenital muscular dystrophy | | | | | | | | | | | | |
| --- | --- | --- | --- | --- | --- | --- | --- | --- | --- | --- | --- | --- |
| **Study** | **Year** | **Country** | **Diagnosis** | **N** | **Mean age (SD); range** | **Genetic Background** | **Type of GMFM** | **Measurement Property Evaluated** | **n** | **Results** | **COSMIN**  **BOX** |  |
| Sato et al.^22^ | 2017 | Japan | Fukuyama congenital muscular dystrophy (FCMD) | 41 | 8.6; 0.6–24.4 years | Homozygous founder: 33  Compound heterozygous: 8 | GMFM-88 Total (raw) | Inter-rater reliability | 20 | ICC = 0.9739–0.9979 | 6 |  |
|  |  |  |  |  |  |  |  | Construct validity  (Comparison with Modified Ueda classification) | 41 | rho = 0.930 | 9a |  |
|  |  |  |  |  |  |  |  | Construct validity  (Comparison between three phenotype groups) | 41 | GMFM scores were significantly higher for the mild phenotype, followed in order by the typical and severe phenotypes. | 9b |  |
|  |  |  |  |  |  |  |  | Construct validity  (Comparison with NPPV status) | 41 | NPPV group was significantly lower than non-NPPV group. | 9b |  |
| Sato et al.^24^ | 2020 | Japan | FCMD | 15 | 7.0; 2.0–15.3 years | Homozygous founder: 12  Compound heterozygous: 3 | GMFM-FCMD (raw) | Structural validity | 100^a^ | Rasch analysis of the GMFM for FCMD identified 18 misfit items, and after clinical review, 20 items were ultimately excluded, resulting in a modified 68-item GMFM version. | 3 |  |
|  |  |  |  |  |  |  |  | Construct validity  (Comparison with Ueda classification) | 15 | r = 0.935 | 9a |  |
|  |  |  |  |  |  |  |  | Construct validity  (Comparison with original GMFM) | 15 | r = 0.9951 | 9a |  |
| Abbreviations: COSMIN, COnsensus-based Standards for the selection of health Measurement INstruments; FCMD, Fukuyama Congenital Muscular Dystrophy; GMFM, Gross Motor Function Measure; GMFM-FCMD, Gross Motor Function Measure for Fukuyama Congenital Muscular Dystrophy; ICC, Intraclass Correlation Coefficient; N, total number of participants; n, number of participants in specific analysis; NPPV, Non-invasive Positive Pressure Ventilation; r, Pearson correlation coefficient; rho, Spearman's rank correlation coefficient; SD, standard deviation.  ^a^ Sample size was reported as 100, but may represent multiple assessments of the same participants from Sato et al. 2017 study (n = 41). | | | | | | | | | | | |  |

Risk of bias and quality assessment for structural validity of the Gross Motor Function Measure in children with Fukuyama congenital muscular dystrophy

| Risk of Bias and structural validity assessment (GMFM-FCMD) | | | |
| --- | --- | --- | --- |
| ***Box 3. Structural validity*** | | Sato et al. 2020 | |
|  |  | Consensus | Rating Justification |
| 1 | For CTT: Was exploratory or confirmatory factor analysis performed? | NA |  |
| 2 | For IRT/Rasch: does the chosen model fit to the research question? | A | The study used a Rasch model |
| 3 | Was the sample size included in the analysis adequate? | I | It states n = 100, but the previous study had n = 41, suggesting the possibility that the same subjects were assessed multiple times. |
| 4 | Were there any other important flaws? | VG | No major defects. |
| **QUALITY OF THE STUDY** *Lowest score of standards 1-4* | | **I** |  |
| **Rating** | | **＋** | The study meets appropriate model fit criteria and reports detailed item fit data. |

| GRADE evaluation of structural validity study (GMFM-FCMD) | | |
| --- | --- | --- |
| Item | Judge | Justification |
| Risk of bias | −3: Extremely serious | Inadequate quality only |
| Inconsistency | Non | Only one study |
| Imprecision | −2: total n<50 | Total sample size=41 |
| Indirectness | Non | Study population directly matched the review question. |
| **GRADE** | **Very Low** | −5 grade down |
| **Rating** | **＋** | Sufficient (＋) rating only |

Abbreviations: CTT, Classical Test Theory; GMFM, Gross Motor Function Measure; GMFM-FCMD, Gross Motor Function Measure for Fukuyama Congenital Muscular Dystrophy; GRADE, Grading of Recommendations Assessment, Development and Evaluation; I, inadequate; IRT, Item Response Theory; n, number of participants; NA, not applicable; VG, very good; +, sufficient rating.

Risk of bias and quality assessment for reliability of the Gross Motor Function Measure in children with Fukuyama congenital muscular dystrophy

| Risk of Bias and reliability assessment (GMFM-88) | | | |
| --- | --- | --- | --- |
| ***Box 6. Reliability*** | | Sato et al. 2017 | |
|  |  | Inter-rater reliability | |
|  |  | Consensus | Rating Justification |
| 1 | Were patients stable in the time between the repeated measurements on the construct to be measured? | NA |  |
| 2 | Was the time interval between the measurements appropriate? | NA |  |
| 3 | Were the measurement conditions similar for the measurements – except for the condition being evaluated as a source of variation? | A | As video assessment was used, it is considered that there was no variation in the condition being measured. |
| 4 | Did the professional(s) administer the measurement without knowledge of scores or values of other repeated measurement(s) in the same patients? | VG | The assessments were blinded. |
| 5 | 5. Did the professional(s) assign scores or determine values without knowledge of the scores or values of other repeated measurement(s) in the same patients? | VG | The assessments were blinded, and the influence between raters was minimal. |
| 6 | Were there any other important flaws in the design or statistical methods of the study? | VG | No major defects. |
| 7 | For continuous scores: was an intraclass correlation coefficient (ICC) calculated? | A | No description of ICC model or formula. |
| 8 | For ordinal scores: was a (weighted) kappa calculated? | NA |  |
| 9 | For dichotomous/nominal scores: was Kappa calculated for each category against the other categories combined? | NA |  |
| **QUALITY OF THE STUDY** *Lowest score of standards 1-7* | | **A** |  |
| **Rating** | | **＋** | ICC ≥ 0.70 |

| GRADE evaluation of reliability study (GMFM-88) | | |
| --- | --- | --- |
| Item | Judge | Justification |
| Risk of bias | −1: Serious | Adequate quality only. |
| Inconsistency | Non | Only one study |
| Imprecision | −2: total n<50 | Total sample size=20 |
| Indirectness | Non | Study population directly matched the review question. |
| **GRADE** | **Very Low** | −3 grade down |
| **Rating** | **＋** | Only sufficient (＋) rating |

Abbreviations: A, adequate; GMFM, Gross Motor Function Measure; GRADE, Grading of Recommendations Assessment, Development and Evaluation; ICC, Intraclass Correlation Coefficient; n, number of participants; NA, not applicable; VG, very good; +, sufficient rating.

Risk of bias and quality assessment for construct validity of the Gross Motor Function Measure in children with Fukuyama congenital muscular dystrophy

| Risk of Bias and construct validity assessment (GMFM-88) | | | | | | |
| --- | --- | --- | --- | --- | --- | --- |
| ***Box 9. Hypotheses testing for construct validity*** | | Sato et al. 2017 | |  |  |  |
| **9a. Comparison with other outcome measurement instruments (convergent validity)** | | Comparison with Modified Ueda classification | |  |  |  |
|  |  | Consensus | Rating Justification |  |  |  |
| 1 | Is it clear what the comparator instrument(s) measure(s)? | VG | The Ueda classification categorizes FCMD motor function into 9 levels. |  |  |  |
| 2 | Were the measurement properties of the comparator instrument(s) sufficient? | D | Previous research exists that has used the Ueda classification to monitor the clinical course. |  |  |  |
| 3 | Were design and statistical methods adequate for the comparisons being made? | A | The magnitude of correlation and other aspects were not considered. |  |  |  |
| 4 | Were there any other important flaws? | D | There is a possibility that multiple data points from the same subjects were used. |  |  |  |
| **QUALITY OF THE STUDY** *Lowest score of standards 1-4* | | **D** |  |  |  |  |
| **Rating** | | **＋** | The review team hypothesized that the correlation coefficient would be >0.7. |  |  |  |
|  |  | Sato et al. 2017 | | Sato et al. 2017 | |  |
| **9b. Comparison between subgroups (discriminative or known-groups validity)** | | Comparison between three phenotype groups | | Comparison with NPPV status | |  |
|  |  | Consensus | Rating Justification | Consensus | Rating Justification |  |
| 5 | Was an adequate description provided of important characteristics of the subgroups? | D | Only the number of subjects in each group is reported. | D | Only the number of subjects in each group is reported. |  |
| 6 | Were design and statistical methods adequate for the subgroups being compared? | D | Only p-values are reported. | D | Only p-values are reported. |  |
| 7 | Were there any other important flaws? | D | There is a possibility that multiple data points from the same subjects were used. | D | There is a possibility that multiple data points from the same subjects were used. |  |
| **QUALITY OF THE STUDY** *Lowest score of standards 5-7* | | **D** |  | **D** |  |  |
| **Rating** | | **＋** | The review team hypothesized that the correlation coefficient would be >0.7. | **＋** | The review team hypothesized that GMFM scores would be higher in non-NPPV subjects. |  |

| GRADE evaluation of construct validity study (GMFM-88) | | |
| --- | --- | --- |
| Item | Judge | Justification |
| Risk of bias | −1: Serious | One study (3 boxes) of doubtful quality only. |
| Inconsistency | Non | Only one study |
| Imprecision | −2: total n<50 | Total sample size=41 |
| Indirectness | Non | Study population directly matched the review question. |
| **GRADE** | **Very Low** | −3 grade down |
| **Rating** | **＋** | Only sufficient (＋) rating |

Abbreviations: D, doubtful; FCMD, Fukuyama Congenital Muscular Dystrophy; GMFM, Gross Motor Function Measure; GRADE, Grading of Recommendations Assessment, Development and Evaluation; n, number of participants; NPPV, Non-invasive Positive Pressure Ventilation; VG, very good; +, sufficient rating.

Risk of bias and quality assessment for construct validity of the Gross Motor Function Measure in children with Fukuyama congenital muscular dystrophy

| Risk of Bias and construct validity assessment (GMFM-FCMD) | | | | | | |
| --- | --- | --- | --- | --- | --- | --- |
| ***Box 9. Hypotheses testing for construct validity*** | | Sato et al. 2020 | | Sato et al. 2020 | |  |
| **9a. Comparison with other outcome measurement instruments (convergent validity)** | | Comparison with Ueda classification | | Comparison with original GMFM | |  |
|  |  | Consensus | Rating Justification | Consensus | Rating Justification |  |
| 1 | Is it clear what the comparator instrument(s) measure(s)? | VG | The Ueda classification categorizes FCMD motor function into 9 levels. | VG | The measure evaluates gross motor function. |  |
| 2 | Were the measurement properties of the comparator instrument(s) sufficient? | D | Previous research exists that has used the Ueda classification to monitor the clinical course. | A | It is unclear whether GMFM is applicable to the target population. |  |
| 3 | Were design and statistical methods adequate for the comparisons being made? | A | No reporting of mean values or standard deviations. | A | No reporting of mean values or standard deviations. |  |
| 4 | Were there any other important flaws? | D | Two subjects underwent two assessments, but there is no specification of how these repeated measures were analyzed. | D | Two subjects underwent two assessments, but there is no specification of how these repeated measures were analyzed. |  |
| **QUALITY OF THE STUDY** *Lowest score of standards 1-4* | | **D** |  | **D** |  |  |
| **Rating** | | **＋** | The review team hypothesized that the correlation coefficient would be >0.7. | **＋** | The review team hypothesized that the correlation coefficient would be >0.7. |  |

| GRADE evaluation of construct validity study (GMFM-FCMD) | | |
| --- | --- | --- |
| Item | Judge | Justification |
| Risk of bias | −2: Very serious | One study (2 boxes) of doubtful quality only. |
| Inconsistency | Non | Only one study |
| Imprecision | −2: total n<50 | Total sample size=15 |
| Indirectness | Non | Study population directly matched the review question. |
| **GRADE** | **Very Low** | −4 grade down |
| **Rating** | **＋** | Only sufficient (＋) rating |

Abbreviations: A, adequate; D, doubtful; FCMD, Fukuyama Congenital Muscular Dystrophy; GMFM, Gross Motor Function Measure; GMFM-FCMD, Gross Motor Function Measure for Fukuyama Congenital Muscular Dystrophy; GRADE, Grading of Recommendations Assessment, Development and Evaluation; n, number of participants; VG, very good; +, sufficient rating.
