# Supplementary material for: Application of the Gross Motor Function Measure in children with conditions other than cerebral palsy: A systematic review
Source: Dev Med Child Neurol. 2025 Aug 14;67(11):1421–42. doi: 10.1111/dmcn.16465 (PMC12521613; doi:10.1111/dmcn.16465)
Supplement: Supplementary file 6 — Table S5: Measurement properties of the Gross Motor Function Measure in children with Down syndrome [file DMCN-67-1421-s004.docx]

Table S5. Measurement properties of the Gross Motor Function Measure in children with Down Syndrome

| Study characteristics and measurement property findings for the Gross Motor Function Measure in children with Down Syndrome | | | | | | | | | | | |  |  |
| --- | --- | --- | --- | --- | --- | --- | --- | --- | --- | --- | --- | --- | --- |
| **Study** | **Year** | **Country** | **Diagnosis** | **N** | **Mean age (SD); range** | **Type of GMFM** | **Genetic Background** | **Measurement Property Evaluated** | **n** | **Results** | **COSMIN**  **BOX** | | |
| Russell et al.^25^ | 1998 | Canada | Down Syndrome | 123 | 28.7; 1.7–72.0 months | GMFM-88 A–E (%)  GMFM-88 Total (%) | Trisomy 21: 81.1%  Translocation: 4.9%  Mosaic: 2.5%  Mixed: 0.8%  Unknown: 11.4% | Test-retest reliability | 22 | Standard scoring: ICC = 0.95 (total)  ICC = 0.62-0.98 (A-E)  Reported scoring^a^: ICC = 0.96 (total)  ICC = 0.87-0.99 (A-E) | 6 | | |
|  |  |  |  |  |  |  |  | Inter-rater reliability | 22 | Standard scoring: ICC = 0.96 (total)  ICC = 0.73-0.98 (A-E)  Reported scoring: ICC = 0.98 (total)  ICC = 0.82-0.99 (A-E) | 6 | | |
|  |  |  |  |  |  |  |  | Responsiveness  (Comparison with BSID-II) | 110 | Standard scoring: GMFM demonstrated larger changes in lower severity groups, while BSID-II showed no consistent pattern across groups.  Reported scoring: Enhanced responsiveness with more marked trends across age/severity groups compared to standard scoring. | 10b | | |
|  |  |  |  |  |  |  |  | Responsiveness  (Comparison with judgement by person) | 117 (Parent)  80 (Intervenor)  30 (Video) | Standard scoring: Total GMFM correlations below criterion (Parent r=0.16, Intervenor r=0.24, Video r=0.23; criterion r>0.60).  Reported scoring: Improved correlations (Parent r=0.52, Intervenor r=0.40, Video r=0.34), stronger than standard administration but still below criterion for total score. | 10b | | |
|  |  |  |  |  |  |  |  | Responsiveness  (Comparison among ages and among severities) | 123 | Standard scoring: Significant gradient - Group 1 (young/mild): 15% improvement vs Group 4 (older/moderate-severe): 7% improvement.  Reported scoring: More pronounced gradient - Group 1: 16% improvement vs Group 4: 6% improvement. | 10c | | |
| Abbreviations: BSID-II, Bayley Scales of Infant Development-II; COSMIN, COnsensus-based Standards for the selection of health Measurement INstruments; GMFM, Gross Motor Function Measure; ICC, Intraclass Correlation Coefficient; N, total number of participants; n, number of participants in specific analysis; SD, standard deviation.  ^e^ GMFM-DS refers to GMFM-88 using reported scoring method for children with Down syndrome | | | | | | | | | | | | |  |

Risk of bias and quality assessment for reliability of the Gross Motor Function Measure in children with Down Syndrome

| Risk of Bias and reliability assessment (GMFM-DS & GMFM-88) | | | | | |
| --- | --- | --- | --- | --- | --- |
| ***Box 6. Reliability*** | | Russell et al. | | Russell et al. | |
|  |  | Test-retest reliability | | Inter-rater reliability | |
|  |  | Consensus | Rating Justification | Consensus | Rating Justification |
| 1 | Were patients stable in the time between the repeated measurements on the construct to be measured? | VG | Children continued to receive their usual programs during the maximum 2-week interval between assessments. | NA |  |
| 2 | Was the time interval between the measurements appropriate? | VG | The time interval between measurements was a maximum of 2 weeks. | NA |  |
| 3 | Were the measurement conditions similar for the measurements – except for the condition being evaluated as a source of variation? | D | It is unclear whether habituation to measurement was considered. | D | It is unclear whether habituation to measurement was considered. |
| 4 | Did the professional(s) administer the measurement without knowledge of scores or values of other repeated measurement(s) in the same patients? | VG | There is documentation regarding blinding to scores. | VG | There is documentation regarding blinding to scores. |
| 5 | 5. Did the professional(s) assign scores or determine values without knowledge of the scores or values of other repeated measurement(s) in the same patients? | VG | The circumstances make it unlikely that raters knew the scores of other assessments. | VG | The circumstances make it unlikely that raters knew the scores of other assessments. |
| 6 | Were there any other important flaws in the design or statistical methods of the study? | VG | No major defects. | VG | No major defects. |
| 7 | For continuous scores: was an intraclass correlation coefficient (ICC) calculated? | VG | ICC model was properly explained. | VG | ICC model was properly explained. |
| 8 | For ordinal scores: was a (weighted) kappa calculated? | NA |  | NA |  |
| 9 | For dichotomous/nominal scores: was Kappa calculated for each category against the other categories combined? | NA |  | NA |  |
| **QUALITY OF THE STUDY** *Lowest score of standards 1-7* | | **D** |  | **D** |  |
| **Rating** | | **＋** | Most results showed ICC values greater than 0.75. | **＋** | Most results showed ICC values greater than 0.75. |

| GRADE evaluation of reliability study (GMFM-DS & GMFM-88) | | |
| --- | --- | --- |
| Item | Judge | Justification |
| Risk of bias | −2: Very serious | One study (2 boxes) of doubtful quality only. |
| Inconsistency | Non | Only one study |
| Imprecision | −2: total n<50 | Total sample size=22 |
| Indirectness | Non | Study population directly matched the review question. |
| **GRADE** | **Very Low** | −3 grade down |
| **Rating** | **＋** | Only sufficient (＋) rating |

Abbreviations: D, doubtful; GMFM, Gross Motor Function Measure; GRADE, Grading of Recommendations Assessment, Development and Evaluation; ICC, Intraclass Correlation Coefficient; n, number of participants; NA, not applicable; VG, very good; +, sufficient rating.

Risk of bias and quality assessment for responsiveness of the Gross Motor Function Measure in children with Down Syndrome

| Risk of Bias and responsiveness assessment (GMFM-DS & GMFM-88) | | | | | |
| --- | --- | --- | --- | --- | --- |
| ***Box 10. Responsiveness*** | | Russell et al. | | Russell et al. | |
| **10b. Construct approach (comparison with other outcome measurement instruments)** | | Comparison with BSID-II | | Comparison with judgement by person | |
|  |  | Consensus | Rating Justification | Consensus | Rating Justification |
| 4 | Is it clear what the comparator instrument(s) measure(s)? | VG | BSID-II has a clear construct as a motor development assessment. | VG | The purpose of evaluating gross motor function is clearly defined. |
| 5 | Were the measurement properties of the comparator instrument(s) sufficient? | VG | The measurement properties of BSID-II are considered sufficient. | A | Moderate reliability has been confirmed. |
| 6 | Were design and statistical methods adequate for the comparisons being made? | A | It is unclear whether adjustments for multiple comparisons were made. | A | Hypotheses were formulated and considered appropriate. |
| 7 | Were there any other important flaws? | VG | No major defects. | VG | No major defects. |
| **QUALITY OF THE STUDY** *Lowest score of standards 4-7* | | **A** |  | A |  |
| **Rating** | | **＋** | Conformed to the original authors' hypothesis | − | Fell below the original authors' hypothesis |
|  |  | Russell et al. | |  | |
|  |  | Comparison among ages and among severities | |  |  |
| **10c. Construct approach: (comparison between subgroups)** | | Consensus | Rating Justification |  |  |
| 8 | Was an adequate description provided of important characteristics of the subgroups? | A | Characteristics of subgroups are barely described. |  |  |
| 9 | Were design and statistical methods adequate for the subgroups being compared? | I | t-tests were performed, which was judged to be insufficient. |  |  |
| 10 | Were there any other important flaws? | VG | No major defects. |  |  |
| **QUALITY OF THE STUDY** *Lowest score of standards 8-10* | | **I** |  |  |  |
| **Rating** | | **＋** | Conformed to the original authors' hypothesis |  |  |

| GRADE evaluation of responsiveness study (GMFM-DS & GMFM-88) | | |
| --- | --- | --- |
| Item | Judge | Justification |
| Risk of bias | −2: Very serious | Due to the presence of both Inadequate and Adequate quality. |
| Inconsistency | −1: Serious | There were inconsistencies in the results. |
| Imprecision | Non | Total sample size=123 |
| Indirectness | Non | Study population directly matched the review question. |
| **GRADE** | **Very Low** | −3 grade down |
| **Rating** | **±** | There were inconsistencies in the results. |

Abbreviations: A, adequate; BSID-II, Bayley Scales of Infant Development-II; GMFM, Gross Motor Function Measure; GRADE, Grading of Recommendations Assessment, Development and Evaluation; I, inadequate; n, number of participants; VG, very good; +, sufficient rating; ? insufficient rating; ±, inconsistent rating.
