# Supplementary material for: Application of the Gross Motor Function Measure in children with conditions other than cerebral palsy: A systematic review
Source: Dev Med Child Neurol. 2025 Aug 14;67(11):1421–42. doi: 10.1111/dmcn.16465 (PMC12521613; doi:10.1111/dmcn.16465)
Supplement: Supplementary file 7 — Table S6: Measurement properties of the Gross Motor Function Measure in children with osteogenesis imperfecta [file DMCN-67-1421-s003.docx]

Table S6. Measurement properties of the Gross Motor Function Measure in children with osteogenesis imperfecta

| Study characteristics and measurement property findings for the Gross Motor Function Measure in children with osteogenesis imperfecta | | | | | | | | | | | |  |
| --- | --- | --- | --- | --- | --- | --- | --- | --- | --- | --- | --- | --- |
| **Study** | **Year** | **Country** | **Diagnosis** | **N** | **Mean age (SD); range** | **OI type distribution** | **Type of GMFM** | **Measurement Property Evaluated** | **n** | **Results** | **COSMIN**  **BOX** | |
| Ruck-Gibis et al.^36^ | 2001 | Canada | Osteogenesis imperfecta | 19 | 7.89 years; 8 months–17 years 11 months | Type I: 2  Type III: 9  Type IV: 8 | GMFM-88 A-E (%)  GMFM-88 Total (%) | Inter-rater reliability | 19 | ICC = 0.99 (total)  ICC = 0.98 (A) to 0.99 (B-E) | 6 | |
|  |  |  |  |  |  |  |  | Intra-rater reliability | 19 | ICC = 0.99 (Total & A-E) | 6 | |
| Abbreviations: COSMIN, COnsensus-based Standards for the selection of health Measurement INstruments; GMFM, Gross Motor Function Measure; ICC, Intraclass Correlation Coefficient; N, total number of participants; n, number of participants in specific analysis; OI, Osteogenesis Imperfecta; SD, standard deviation. | | | | | | | | | | | | |

Risk of bias and quality assessment for reliability of the Gross Motor Function Measure in children with osteogenesis imperfecta

| Risk of Bias and reliability assessment | | | | | |
| --- | --- | --- | --- | --- | --- |
| ***Box 6. Reliability*** | | Ruck-Gibis et al. | | Ruck-Gibis et al. | |
|  |  | Inter-rater reliability | | Intra-rater reliability | |
|  |  | Consensus | Rating Justification | Consensus | Rating Justification |
| 1 | Were patients stable in the time between the repeated measurements on the construct to be measured? | NA |  | NA |  |
| 2 | Was the time interval between the measurements appropriate? | VG | It was implemented independently over a specific period. | VG | The minimum interval of six weeks was appropriate. |
| 3 | Were the measurement conditions similar for the measurements – except for the condition being evaluated as a source of variation? | VG | Conditions were standardized due to the video-based evaluation. | VG | Although there was a mix of direct evaluation and video evaluation, other conditions were deemed consistent. |
| 4 | Did the professional(s) administer the measurement without knowledge of scores or values of other repeated measurement(s) in the same patients? | VG | Since most evaluations were video-based, the influence from other measurements was deemed minimal. | VG | Since most evaluations were video-based, the influence from other measurements was deemed minimal. |
| 5 | 5. Did the professional(s) assign scores or determine values without knowledge of the scores or values of other repeated measurement(s) in the same patients? | VG | Raters were instructed not to discuss their scores. | VG | The measurement interval was appropriate, with no significant influence expected. |
| 6 | Were there any other important flaws in the design or statistical methods of the study? | VG | No major defects. | VG | No major defects. |
| 7 | For continuous scores: was an intraclass correlation coefficient (ICC) calculated? | VG | ICC model was properly explained. | VG | ICC model was properly explained. |
| 8 | For ordinal scores: was a (weighted) kappa calculated? | NA |  | NA |  |
| 9 | For dichotomous/nominal scores: was Kappa calculated for each category against the other categories combined? | NA |  | NA |  |
|  | **QUALITY OF THE STUDY** *Lowest score of standards 1-7* | **VG** |  | **VG** |  |
| **Rating** | | **＋** | ICC ≥ 0.70 | **＋** | ICC ≥ 0.70 |

| GRADE evaluation of reliability study | | |
| --- | --- | --- |
| Item | Judge | Justification |
| Risk of bias | Non | Very good quality only. |
| Inconsistency | Non | Only one study |
| Imprecision | −2: total n<50 | Total sample size=19 |
| Indirectness | Non | Study population directly matched the review question. |
| **GRADE** | **Low** | −2 grade down |
| **Rating** | **＋** | Only sufficient (＋) rating |

Abbreviations: GMFM, Gross Motor Function Measure; GRADE, Grading of Recommendations Assessment, Development and Evaluation; ICC, Intraclass Correlation Coefficient; n, number of participants; NA, not applicable; VG, very good; +, sufficient rating.
