# Supplementary material for: Application of the Gross Motor Function Measure in children with conditions other than cerebral palsy: A systematic review
Source: Dev Med Child Neurol. 2025 Aug 14;67(11):1421–42. doi: 10.1111/dmcn.16465 (PMC12521613; doi:10.1111/dmcn.16465)
Supplement: Supplementary file 8 — Table S7: Measurement properties of the Gross Motor Function Measure in children with acute lymphoblastic leukemia [file DMCN-67-1421-s008.docx]

Table S7. Measurement properties of the Gross Motor Function Measure in children with acute lymphoblastic leukemia

| Study characteristics and measurement property findings for the Gross Motor Function Measure in children with acute lymphoblastic leukemia | | | | | | | | | | | | |  |
| --- | --- | --- | --- | --- | --- | --- | --- | --- | --- | --- | --- | --- | --- |
| **Study** | **Year** | **Country** | **Diagnosis** | **N** | **Mean age (SD); range** | **Risk Stratification** | **Type of GMFM** | **Measurement Property Evaluated** | **n** | **Results** | **COSMIN**  **BOX** | | |
| Wright et al.^26^ | 2007 | Canada | Acute lymphoblastic leukemia (ALL) |  |  |  | GMFM-ALL | Content validity  Relevance |  | Selected items demonstrated high clinical utility scores (≥19) based on three pediatric oncology physiotherapists' evaluation, with strong correlations with the original GMFM (D: r = 0.977, E: r = 0.983, both p<0.001). | 2d | | |
|  |  |  |  |  |  |  |  | Content validity Comprehensiveness |  | GMFM-ALL retained 20 items (D: 7, E: 13) from the original 37 items, showing high reliability (0.90) and accommodating various functional levels including children with lower functioning. | 2e | | |
|  |  |  |  | 91 | median 8.5;  2.8–15.9 years^a^ | Standard risk: 12^c^  High risk: 8  (Only 13 of the 20 children completed two consecutive trials with consistent evaluators.) | GMFM-ALL (%)  GMFM-88 D-E (%) | Inter-rater reliability | 13 | Generalizability Coefficients = 0.99 | 6 |  |  |
|  |  |  |  |  |  |  |  | Test-retest reliability | 13 | Generalizability Coefficients = 0.94–0.97 | 6 | | |
|  |  |  |  |  |  |  |  | Construct validity  (Comparison with GMFM-88 D–E) | 91 | Dimension D: r = 0.977  Dimension E: r = 0.983 | 9a | | |
|  |  |  |  |  |  |  |  | Responsiveness  (Comparison between time points) | 39 | There was a significant change in scores between the first and second time. | 10d | | |
|  |  |  |  |  |  |  |  | Responsiveness  (Comparison between age groups) | 39 | There was a significant difference in the change in scores between younger and older children for dimension D scores. There was no significant difference, for dimension E scores. | 10c | | |
| Abbreviations: ALL, Acute Lymphoblastic Leukemia; COSMIN, COnsensus-based Standards for the selection of health Measurement INstruments; GMFM, Gross Motor Function Measure; GMFM-ALL, Gross Motor Function Measure for Acute Lymphoblastic Leukemia; N, total number of participants; n, number of participants in specific analysis; r, Pearson correlation coefficient; SD, standard deviation.  ^a^ Age of subset participants | | | | | | | | | | | | | |

Risk of bias and quality assessment for content validity of the Gross Motor Function Measure in children with acute lymphoblastic leukemia

| Risk of Bias and content validity assessment | | | |  |  |
| --- | --- | --- | --- | --- | --- |
| **Box 2. Content validity** | | Wright et al. | | |  |
| ***2d. Asking professionals about relevance*** | | Consensus | Rating Justification |  |  |
| 22 | Was an appropriate method used to ask professionals whether each item is relevant for the construct of interest? | A | Multiple categories were examined using a 7-point Likert scale. |  |  |
| 23 | Were professionals from all relevant disciplines included? | D | Three physiotherapists working in pediatric oncology were involved |  |  |
| 24 | Was each item tested in an appropriate number of professionals? | D | There were three professionals. |  |  |
| 25 | Was an appropriate approach used to analyse the data? | VG | Systematic quantitative analysis with clear criteria for item relevance assessment. |  |  |
| 26 | Were at least two researchers involved in the analysis? | D | How the analysis was conducted is unclear/uncertain |  |  |
|  | **QUALITY OF THE STUDY** *Lowest score of standards 22-26* | **D** |  |  |  |
|  |  | Wright et al. | | |  |
| ***2e. Asking professionals about comprehensiveness*** | | Consensus | Rating Justification | | |
| 27 | Was an appropriate method used for assessing the comprehensiveness of the PROM? | A | Enjoyment, safety, and ALL-specific impairments are reflected/considered. | | |
| 28 | Were professionals from all relevant disciplines included? | D | Three physiotherapists working in pediatric oncology were involved | | |
| 29 | Was each item tested in an appropriate number of professionals? | D | There were three professionals. | | |
| 30 | Was an appropriate approach used to analyse the data? | A | Analysis was conducted using clinical utility scores and Pearson correlation coefficients. | | |
| 31 | Were at least two researchers involved in the analysis? | D | Reasonable consideration of relevant domains, but limited systematic assessment of comprehensiveness. | | |
|  | **QUALITY OF THE STUDY** *Lowest score of standards 27-31* | **D** |  | | |

| Content validity evidence synthesis and quality rating for GMFM-ALL | | | | |
| --- | --- | --- | --- | --- |
| **GMFM-ALL** | **Wright et al.**^26^ | **Rating of Reviewers** | **Rating** | **GRADE** |
| **Criteria** | **+/−/±/?** | **+/−/±/?** | **+/−/±** | High, Moderate, Low, Very Low |
| Relevance | | | | |
| 1 | + | + |  |  |
| 2 | ? | + |  |  |
| 3 | + | + |  |  |
| 4 | + | + |  |  |
| 5 | NA | NA |  |  |
| Rating | + | + | + | Moderate |
| Comprehensiveness | | | | |
| 6 | + | + |  |  |
| Rating | + | + | + | Moderate |
| Comprehensibility | | | | |
| 7 | ? |  |  |  |
| 8 | ? |  |  |  |
| 9 |  | + |  |  |
| 10 |  | + |  |  |
| Rating | ? | + | + | Moderate |
| Content validity Rating |  |  | + | Moderate |

**Rating Justification**

Wright et al. study:

-Criteria 1, 3, 4, 6: Systematic quantitative analysis using a 7-point Likert scale showed that ≥85% of items met the criteria for relevance and comprehensiveness.

-Criterion 2: Indeterminate due to lack of relevance evaluation from participants, with only professionals involved in the assessment.

-Criterion 6: ALL-specific impairments, enjoyment, and safety were reflected, and comprehensiveness was confirmed through analysis using clinical utility scores and Pearson correlation coefficients.

-Criteria 7, 8: Indeterminate due to no systematic evaluation of comprehensibility from participants being reported.

Rating of Reviewers:

-Criteria 1-4, 6: Item selection specialized for gross motor function assessment in ALL participants and appropriate evaluation criteria led to the judgment that ≥85% of items met each criterion

-Criteria 9, 10: Item wording is clear, suitable for 12-year-old reading level, and response options appropriately correspond to question content

| GRADE & Rating (GMFM-ALL) | | |
| --- | --- | --- |
| Item | Judge | Justification |
| Risk of bias | −1: Serious | One content validity study of doubtful quality. |
| Inconsistency | Non | Only one content validity study available. No inconsistency between study and reviewers' rating (both rated +). |
| Imprecision | Non | Not applicable for content validity assessment (qualitative research). |
| Indirectness | Non | Study population directly matched the review question. Same construct of interest (gross motor function). |
| **GRADE** | **Moderate** | −1 grade down |
| **Rating** | **+** | Sufficient (+) rating for relevance, comprehensiveness, comprehensibility, and overall content validity |

Abbreviations: A, adequate; ALL, Acute Lymphoblastic Leukemia; D, doubtful; GMFM, Gross Motor Function Measure; GMFM-ALL, Gross Motor Function Measure for Acute Lymphoblastic Leukemia; GRADE, Grading of Recommendations Assessment, Development and Evaluation; NA, not applicable; PROM, Patient-Reported Outcome Measure; r, Pearson correlation coefficient; VG, very good; +, sufficient rating; ?, indeterminate rating.

Risk of bias and quality assessment for reliability of the Gross Motor Function Measure in children with acute lymphoblastic leukemia

| Risk of Bias and reliability assessment (GMFM-ALL & GMFM-88) | | | | | |
| --- | --- | --- | --- | --- | --- |
| ***Box 6. Reliability*** | | Wright et al. | | Wright et al. | |
|  |  | Inter-rater reliability | | Test-retest reliability | |
|  |  | Consensus | Rating Justification | Consensus | Rating Justification |
| 1 | Were patients stable in the time between the repeated measurements on the construct to be measured? | NA |  | VG | Considering the environment and time to be consistent and stable. |
| 2 | Was the time interval between the measurements appropriate? | NA |  | VG | Three-week intervals are appropriate. |
| 3 | Were the measurement conditions similar for the measurements – except for the condition being evaluated as a source of variation? | D | It is unclear whether habituation to measurement was considered. | D | It is unclear whether habituation to measurement was considered. |
| 4 | Did the professional(s) administer the measurement without knowledge of scores or values of other repeated measurement(s) in the same patients? | D | No mention of blinding. | D | No mention of blinding. |
| 5 | 5. Did the professional(s) assign scores or determine values without knowledge of the scores or values of other repeated measurement(s) in the same patients? | D | No mention of blinding. | D | No mention of blinding. |
| 6 | Were there any other important flaws in the design or statistical methods of the study? | D | It was carried out in 13 out of 20 eligible participants, but the reasons for drop-outs are not stated. | D | It was carried out in 13 out of 20 eligible participants, but the reasons for drop-outs are not stated. |
| 7 | For continuous scores: was an intraclass correlation coefficient (ICC) calculated? | A | Calculated with Generalizability Coefficients. | A | Calculated with Generalizability Coefficients. |
| 8 | For ordinal scores: was a (weighted) kappa calculated? | NA |  | NA |  |
| 9 | For dichotomous/nominal scores: was Kappa calculated for each category against the other categories combined? | NA |  | NA |  |
| **QUALITY OF THE STUDY** *Lowest score of standards 1-7* | | **D** |  | **D** |  |
| **Rating** | | **＋** | Generalizability Coefficients ≥ 0.70 | **＋** | Generalizability Coefficients ≥ 0.70 |

| GRADE evaluation of reliability study (GMFM-ALL & GMFM-88) | | | |
| --- | --- | --- | --- |
| Item | Judge | Justification |  |
| Risk of bias | −2: Very serious | One study (2 boxes) of doubtful quality only. |  |
| Inconsistency | Non | Only one study |  |
| Imprecision | −2: total n<50 | Total sample size = 19 |  |
| Indirectness | Non | Age and gender is stated. Study population directly matched the review question. |  |
| **GRADE** | **Very Low** | −4 grade down |  |
| **Rating** | **＋** | Only sufficient (＋) rating |  |

Abbreviations: A, adequate; D, doubtful; GMFM, Gross Motor Function Measure; GMFM-ALL, Gross Motor Function Measure for Acute Lymphoblastic Leukemia; GRADE, Grading of Recommendations Assessment, Development and Evaluation; ICC, Intraclass Correlation Coefficient; n, number of participants; NA, not applicable; +, sufficient rating.

Risk of bias and quality assessment for construct validity of the Gross Motor Function Measure in children with acute lymphoblastic leukemia

| Risk of Bias and construct validity assessment | | | |
| --- | --- | --- | --- |
| ***Box 9. Hypotheses testing for construct validity*** | | Wright et al. | |
| **9a. Comparison with other outcome measurement instruments (convergent validity)** | | Comparison with GMFM-88 D–E (GMFM-ALL) | |
|  |  | Consensus | Rating Justification |
| 1 | Is it clear what the comparator instrument(s) measure(s)? | VG | The measure evaluates gross motor function. |
| 2 | Were the measurement properties of the comparator instrument(s) sufficient? | A | It is unclear whether GMFM is applicable to the target population. |
| 3 | Were design and statistical methods adequate for the comparisons being made? | VG | Means and standard deviations of the data are described. |
| 4 | Were there any other important flaws? | VG | No major defects. |
| **QUALITY OF THE STUDY** *Lowest score of standards 1-4* | | **A** |  |
| **Rating** | | **＋** | The review team hypothesized that the correlation coefficient would be >0.7. |

| GRADE evaluation of construct validity study (GMFM-ALL) | | |
| --- | --- | --- |
| Item | Judge | Justification |
| Risk of bias | −1: Serious | Adequate quality only. |
| Inconsistency | Non | Only one study |
| Imprecision | −1: total n=50-100 | Total sample size = 91 |
| Indirectness | −1: Serious | No description of basic information such as age and gender. |
| **GRADE** | **Very Low** | −3 grade down |
| **Rating** | **＋** | Only sufficient (＋) rating |

Abbreviations: A, adequate; GMFM, Gross Motor Function Measure; GMFM-ALL, Gross Motor Function Measure for Acute Lymphoblastic Leukemia; GRADE, Grading of Recommendations Assessment, Development and Evaluation; n, number of participants; VG, very good; +, sufficient rating.

Risk of bias and quality assessment for responsiveness of the Gross Motor Function Measure in children with acute lymphoblastic leukemia

| Risk of Bias and responsiveness assessment | | | | | | |
| --- | --- | --- | --- | --- | --- | --- |
| ***Box 10. Responsiveness*** | | Wright et al. | | | |  |
|  |  | Comparison between age groups (GMFM-ALL & GMFM-88) | | | |  |
| **10c. Construct approach: (comparison between subgroups)** | | Consensus | Rating Justification | |  |  |
| 8 | Was an adequate description provided of important characteristics of the subgroups? | D | No description of the characteristics of each group. | |  |  |
| 9 | Were design and statistical methods adequate for the subgroups being compared? | I | Tested with Paired t-test. | |  |  |
| 10 | Were there any other important flaws? | VG | No major defects. | |  |  |
|  | **QUALITY OF THE STUDY** *Lowest score of standards 8-10* | **I** |  | |  |  |
| **Rating** | | **−** | The review team hypothesised that the change would be greater at younger ages in dimensions D & E. | |  |  |
| **10d. Construct approach: (comparison before and after intervention)** | | Wright et al. | | | |  |
|  |  | Comparison between time points (GMFM-ALL & GMFM-88) | | | |  |
|  |  | Consensus | | Rating Justification | |  |
| 11 | Was an adequate description provided of the intervention given? | VG | | Chemotherapy is described in detail. | |  |
| 12 | Was the statistical method appropriate for the before-after comparison being made? | I | | Tested with Paired t-test. | |  |
| 13 | Were there any other important flaws? | VG | | No major defects. | |  |
| **QUALITY OF THE STUDY** *Lowest score of standards 11-13* | | **I** | |  | |  |
| **Rating** | | **＋** | | The review team hypothesised that there would be improvements in GMFM. | |  |

| GRADE evaluation of responsiveness study (GMFM-ALL & GMFM-88) | | |
| --- | --- | --- |
| Item | Judge | Justification |
| Risk of bias | −2: Very serious | One study (2 boxes) of inadequate quality only. |
| Inconsistency | Non | Only one study |
| Imprecision | −2: total n<50 | Total sample size = 39 |
| Indirectness | −1: Serious | No description of basic information such as age and gender. |
| **GRADE** | **Very Low** | −5 grade down |
| **Rating** | **±** | There were inconsistencies in the results. |

Abbreviations: D, doubtful; GMFM, Gross Motor Function Measure; GMFM-88, Gross Motor Function Measure-88; GMFM-ALL, Gross Motor Function Measure for Acute Lymphoblastic Leukemia; GRADE, Grading of Recommendations Assessment, Development and Evaluation; I, inadequate; n, number of participants; VG, very good; +, sufficient rating; -, insufficient rating; ±, inconsistent rating.
