# Supplementary material for: Application of the Gross Motor Function Measure in children with conditions other than cerebral palsy: A systematic review
Source: Dev Med Child Neurol. 2025 Aug 14;67(11):1421–42. doi: 10.1111/dmcn.16465 (PMC12521613; doi:10.1111/dmcn.16465)
Supplement: Supplementary file 9 — Table S8: Measurement properties of the Gross Motor Function Measure in children with leukodystrophy [file DMCN-67-1421-s010.docx]

Table S8. Measurement properties of the Gross Motor Function Measure in children with leukodystrophy

| Study characteristics and measurement property findings for the Gross Motor Function Measure in children with leukodystrophy | | | | | | | | | | |  |
| --- | --- | --- | --- | --- | --- | --- | --- | --- | --- | --- | --- |
| **Study** | **Year** | **Country** | **Diagnosis** | **N** | **Mean age (SD); range** | **Type of GMFM** | **Measurement Property Evaluated** | **n** | **Results** | **COSMIN**  **BOX** | |
| Gavazzi et al.^23^ | 2021 | United States | Leukodystrophy  ・Aicardi Goutières Syndrome 17  ・POLR3-related leukodystrophy 2  ・Alexander Disease 1  ・TUBB4A-related leukodystrophy 1 | 21 | 9.6 (11.0); 1.3–52.5 years | GMFM-88 Total (raw & %) | Inter-rater reliability | 10 | ICC (95%CI) = 0.996 (0.964–0.999) | 6 | |
|  |  |  |  |  |  |  | Intra-rater reliability | 6 | ICC (95%CI) = 0.999 (0.985–1.000) | 6 | |
|  |  |  |  |  |  |  | Measurement error  (Statistical analysis) | 21 | Limit of agreement = -7.8 to 4.3 (Inter-rater)  Limit of agreement = -2.3 to 2.7 (Intra-rater) | 7 | |
| Abbreviations: CI, Confidence Interval; COSMIN, COnsensus-based Standards for the selection of health Measurement INstruments; GMFM, Gross Motor Function Measure; ICC, Intraclass Correlation Coefficient; N, total number of participants; n, number of participants in specific analysis; SD, standard deviation. | | | | | | | | | | | |

Risk of bias and quality assessment for reliability of the Gross Motor Function Measure in children with leukodystrophy

| Risk of Bias and reliability assessment | | | | | |
| --- | --- | --- | --- | --- | --- |
| ***Box 6. Reliability*** | | Gavazzi et al. | | Gavazzi et al. | |
|  |  | Inter-rater reliability | | Intra-rater reliability | |
|  |  | Consensus | Rating Justification | Consensus | Rating Justification |
| 1 | Were patients stable in the time between the repeated measurements on the construct to be measured? | A | The measurement interval was 2 weeks and subjects were considered to be in a stable condition. | NA |  |
| 2 | Was the time interval between the measurements appropriate? | VG | The measurement interval of 2 weeks was deemed appropriate. | VG | The measurement interval of approximately 1 week was deemed appropriate. |
| 3 | Were the measurement conditions similar for the measurements – except for the condition being evaluated as a source of variation? | VG | Information regarding the environment and documentation was provided to minimize variation. | VG | Information regarding the environment and documentation was provided to minimize variation. |
| 4 | Did the professional(s) administer the measurement without knowledge of scores or values of other repeated measurement(s) in the same patients? | A | It was determined that blind scoring was performed between evaluations. | A | The documentation of blind scoring was considered appropriate. |
| 5 | 5. Did the professional(s) assign scores or determine values without knowledge of the scores or values of other repeated measurement(s) in the same patients? | A | It was considered that assessment was done via remote assessments, and it was determined that no other information was available. | A | Regarding other measurements, no information is provided. |
| 6 | Were there any other important flaws in the design or statistical methods of the study? | VG | No major defects. | VG | No major defects. |
| 7 | For continuous scores: was an intraclass correlation coefficient (ICC) calculated? | VG | ICC model was properly explained. | A | No description of ICC model or formula |
| 8 | For ordinal scores: was a (weighted) kappa calculated? | NA |  | NA |  |
| 9 | For dichotomous/nominal scores: was Kappa calculated for each category against the other categories combined? | NA |  | NA |  |
|  | **QUALITY OF THE STUDY** *Lowest score of standards 1-7* | **A** |  | **A** |  |
| **Rating** | | **＋** | ICC ≥ 0.70 | **＋** | ICC ≥ 0.70 |

| GRADE evaluation of reliability study | | |
| --- | --- | --- |
| Item | Judge | Justification |
| Risk of bias | −1: Serious | One study (2 boxes) of adequate quality only. |
| Inconsistency | Non | Only one study |
| Imprecision | −2: total n<50 | Total sample size=21 |
| Indirectness | Non | Study population directly matched the review question. |
| **GRADE** | **Very Low** | −3 grade down |
| **Rating** | **＋** | Only sufficient (＋) rating |

Abbreviations: A, adequate; GMFM, Gross Motor Function Measure; GRADE, Grading of Recommendations Assessment, Development and Evaluation; ICC, Intraclass Correlation Coefficient; n, number of participants; NA, not applicable; VG, very good; +, sufficient rating.

Risk of bias and quality assessment for measurement error of the Gross Motor Function Measure in children with leukodystrophy

| Risk of Bias and measurement error assessment | | | |
| --- | --- | --- | --- |
| ***Box 7. Measurement error*** | | Gavazzi et al. | |
|  |  | Statistical analysis | |
|  |  | Consensus | Rating Justification |
| 1 | Were patients stable in the time between the repeated measurements on the construct to be measured? | A | The measurement interval was 2 weeks and subjects were considered to be in a stable condition. |
| 2 | Was the time interval between the measurements appropriate? | VG | The measurement interval of 2 weeks or approximately 1 week were deemed appropriate. |
| 3 | Were the measurement conditions similar for the measurements – except for the condition being evaluated as a source of variation? | VG | Information regarding the environment and documentation was provided to minimize variation. |
| 4 | Did the professional(s) administer the measurement without knowledge of scores or values of other repeated measurement(s) in the same patients? | A | It was determined that blind scoring was performed between evaluations. |
| 5 | 5. Did the professional(s) assign scores or determine values without knowledge of the scores or values of other repeated measurement(s) in the same patients? | A | It was considered that assessment was done via remote assessments, and it was determined that no other information was available. |
| 6 | Were there any other important flaws in the design or statistical methods of the study? | VG | No major defects. |
| 7 | For continuous scores: was the Standard Error of Measurement (SEM), Smallest Detectable Change (SDC), Limits of Agreement (LoA) or Coefficient of Variation (CV) calculated? | A | LoA are calculated, but there is no reporting of systematic error. |
| 8 | For dichotomous/nominal/ordinal scores: Was the percentage specific (e.g. positive and negative) agreemnt calculated? | NA |  |
|  | **QUALITY OF THE STUDY** *Lowest score of standards 1-6* | **A** |  |
| **Rating** | | **?** | MIC not defined |

| GRADE evaluation of measurement error study | | |
| --- | --- | --- |
| Item | Judge | Justification |
| Risk of bias | −1: Serious | One study of adequate quality only. |
| Inconsistency | Non | Only one study |
| Imprecision | −2: total n<50 | Total sample size=21 |
| Indirectness | Non | Study population directly matched the review question. |
| **GRADE** | **Very Low** | −3 grade down |
| **Rating** | **?** | MIC not defined |

Abbreviations: A, adequate; CV, Coefficient of Variation; GMFM, Gross Motor Function Measure; GRADE, Grading of Recommendations Assessment, Development and Evaluation; LoA, Limits of Agreement; MIC, Minimal Important Change; n, number of participants; NA, not applicable; SDC, Smallest Detectable Change; SEM, Standard Error of Measurement; VG, very good; ?, indeterminate rating.
