# Supplementary material for: Application of the Gross Motor Function Measure in children with conditions other than cerebral palsy: A systematic review
Source: Dev Med Child Neurol. 2025 Aug 14;67(11):1421–42. doi: 10.1111/dmcn.16465 (PMC12521613; doi:10.1111/dmcn.16465)
Supplement: Supplementary file 10 — Table S9: Measurement properties of the Gross Motor Function Measure in children with Pompe disease [file DMCN-67-1421-s006.docx]

Table S9. Measurement properties of the Gross Motor Function Measure in children with Pompe disease

| Study characteristics and measurement property findings for the Gross Motor Function Measure in children with Pompe disease | | | | | | | | | | | |  |
| --- | --- | --- | --- | --- | --- | --- | --- | --- | --- | --- | --- | --- |
| **Study** | **Year** | **Country** | **Diagnosis** | **N** | **Mean age (SD); range** | **Pompe Motor Function Levels** | **Type of GMFM** | **Measurement Property Evaluated** | **n** | **Results** | **COSMIN**  **BOX** | |
| Duong et al.^37^ | 2022 | United States | Pompe disease | 110 | 5.2 (3.6); 1.0–15.5 years | Level I (Walkers): 23  Level II (Supported walkers): 25  Level III (Supported standers): 15  Level IV (Sitters): 21  Level V (Restricted antigravity movement): 26 | GMFM-88 Total (%) | Measurement error  (Statistical analysis) | 90 | Overall  Mean change: 3.7 ± 17.5  MDC range: 10.0–23.3  Age <2 years (n=19)  Mean change: 21.1 ± 14.1  MDC range: 5.7–13.3  Age ≥2 years (n=71)  Mean change: -0.9 ± 15.3  MDC range: 10.8–25.2 | 7 | |
|  |  |  |  |  |  |  |  | Responsiveness  (Effect size) | 90 | Overall: Effect size: 0.11  Age <2 years (n=19): Effect size: 1.11  Age ≥2 years (n=71): Effect size: -0.03 | 10d | |
| Abbreviations: COSMIN, COnsensus-based Standards for the selection of health Measurement INstruments; GMFM, Gross Motor Function Measure; MDC, Minimal Detectable Change; N, total number of participants; n, number of participants in specific analysis; SD, standard deviation. | | | | | | | | | | | | |

Risk of bias and quality assessment for measurement error of the Gross Motor Function Measure in children with Pompe disease

| Risk of Bias and measurement error assessment | | | |
| --- | --- | --- | --- |
| ***Box 7. Measurement error*** | | Duong et al. | |
|  |  | Statistical analysis | |
|  |  | Consensus | Rating Justification |
| 1 | Were patients stable in the time between the repeated measurements on the construct to be measured? | NA |  |
| 2 | Was the time interval between the measurements appropriate? | NA |  |
| 3 | Were the measurement conditions similar for the measurements – except for the condition being evaluated as a source of variation? | NA |  |
| 4 | Did the professional(s) administer the measurement without knowledge of scores or values of other repeated measurement(s) in the same patients? | D | No information is provided about blinding of assessors to previous measurements. |
| 5 | 5. Did the professional(s) assign scores or determine values without knowledge of the scores or values of other repeated measurement(s) in the same patients? | D | No information is provided about blinding of assessors when assigning scores. |
| 6 | Were there any other important flaws in the design or statistical methods of the study? | VG | No major defects |
| 7 | For continuous scores: was the Standard Error of Measurement (SEM), Smallest Detectable Change (SDC), Limits of Agreement (LoA) or Coefficient of Variation (CV) calculated? | D | This is not the standard method for calculating MDC (SEM). |
| 8 | For dichotomous/nominal/ordinal scores: Was the percentage specific (e.g. positive and negative) agreemnt calculated? | NA |  |
|  | **QUALITY OF THE STUDY** *Lowest score of standards 1-6* | **D** |  |
| **Rating** | | **?** | MIC not defined |

| GRADE evaluation of measurement error study | | |
| --- | --- | --- |
| Item | Judge | Justification |
| Risk of bias | −2: Very serious | One study of doubtful quality only. |
| Inconsistency | Non | Only one study |
| Imprecision | −1: total n=50-100 | Total sample size=90 |
| Indirectness | Non | Only one study |
| **GRADE** | **Very Low** | −3 grade down |
| **Rating** | **?** | MIC not defined |

Abbreviations: CV, Coefficient of Variation; D, doubtful; GMFM, Gross Motor Function Measure; GRADE, Grading of Recommendations Assessment, Development and Evaluation; LoA, Limits of Agreement; MDC, Minimal Detectable Change; MIC, Minimal Important Change; n, number of participants; NA, not applicable; SDC, Smallest Detectable Change; SEM, Standard Error of Measurement; VG, very good; ?, indeterminate rating.

Risk of bias and quality assessment for responsiveness of the Gross Motor Function Measure in children with Pompe disease

| GRADE evaluation of responsiveness study | | |
| --- | --- | --- |
| Item | Judge | Justification |
| Risk of bias | −2: Very serious | One study of doubtful quality only. |
| Inconsistency | Non | Only one study |
| Imprecision | −1: total n=50-100 | Total sample size=90 |
| Indirectness | Non | Only one study |
| **GRADE** | **Very Low** | −3 grade down |
| **Rating** | **＋** | One study with a sufficient (＋) rating |

| Risk of Bias and responsiveness assessment | | | |
| --- | --- | --- | --- |
| ***Box 10. Responsiveness*** | | Duong et al. | |
|  |  | Effect size | |
| **10d. Construct approach: (comparison before and after intervention)** | | Consensus | Rating Justification |
| 11 | Was an adequate description provided of the intervention given? | VG | The intervention is clearly described. |
| 12 | Was the statistical method appropriate for the before-after comparison being made? | D | No hypotheses were established for the analysis. |
| 13 | Were there any other important flaws? | VG | No major defects |
|  | **QUALITY OF THE STUDY** *Lowest score of standards 11-13* | **D** |  |
| Rating | | **＋** | Based on the review team's hypothesis of a moderate effect in children under 2 years of age. |

Abbreviations: D, doubtful; GMFM, Gross Motor Function Measure; GRADE, Grading of Recommendations Assessment, Development and Evaluation; n, number of participants; VG, very good; +, sufficient rating..
