# Supplementary material for: Application of the Gross Motor Function Measure in children with conditions other than cerebral palsy: A systematic review
Source: Dev Med Child Neurol. 2025 Aug 14;67(11):1421–42. doi: 10.1111/dmcn.16465 (PMC12521613; doi:10.1111/dmcn.16465)
Supplement: Supplementary file 11 — Table S10: Non‐measurement studies using the Gross Motor Function Measure in children with conditions other than cerebral palsy [file DMCN-67-1421-s011.docx]

| Table S10. Non-measurement studies using the Gross Motor Function Measure in children with conditions other than cerebral palsy | | | | | | | | | | | | | |
| --- | --- | --- | --- | --- | --- | --- | --- | --- | --- | --- | --- | --- | --- |
| **Diagnosis/Disorders Group** | **Study** | **Year** | **Country**^a^ | **Study design** | **Diagnosis / Disorder** | **Total**  **sample** | **GMFM**^b^  **sample** | **Mean age (SD); range** | **Type of GMFM** | **Purpose of using GMFM** | **GMFM purpose categories** | **Why GMFM was adapted** |  |
| Central Nervous System Disorders (Congenital) | Pacheco et al.^85^ | 2014 | Brazil | Case report | Agenesis of corpus callosum | 1 |  | 2 years | GMFM-88 A-E (%)  GMFM-88 Total (%) | To assess pre- and post-intervention functional performance. | Treatment evaluation | NA (citing CP study) |  |
|  | Jeong et al.^86^ | 2014 | Korea | Case report | Arnold-Chiari Malformation Type III with Meningoencephalocele | 1 |  | 22 months | GMFM-88 A-E (%)  GMFM-88 Total (%) | To provide the details of magnetic resonance imaging, swallowing status and rehabilitation treatment methods. | Treatment evaluation | NA (no citing) |  |
|  | Bican et al.^87^ | 2022 | United States | Case report | Biallelic mutations in the TBCD gene | 1 |  | 23 months and 12 days old | GMFM-88 A-E (raw) | To indicate the post-intervention outcomes that formed the basis for the clinical decision of whether or not to provide high-dose PT intervention. | Treatment evaluation | Describing measurement properties other than CP (citing Nelson, Linder-Lucht, Russell 1989 and Sato 2017 study)  “GMFM-88 has been used as an outcome measure in other pediatric populations.” |  |
|  | Devesa et al.^88^ | 2017 | Spain | Case report | Caudal regression syndrome | 1 |  | 9 months | GMFM-88 Total (%) | To show the progress of the patient's rehabilitation. | Treatment evaluation | NA (no citing) |  |
|  | Arai et al.^89^ | 2022 | Japan | Case report | Cerebellar mutism syndrome (Postoperative ependymoma) | 1 |  | 9 years | GMFM-88 Total (%) | To describe gross motor function in the case. | Natural history and disease progression | NA (citing Russell 1989 study) |  |
|  | Martakis et al.^53^ | 2019 | Germany | A Retrospective Study (within-subjects study) | Congenital ataxia  (subtypes)  Non-progressive ataxia  Progressive ataxia | 45 | 40 | Non-progressive: 7.6 (4.31) years  Progressive: 8.1 (5.96) years | GMFM-66 | To describe changes in motor function after receiving intensive training including vibration-assisted home-training within the rehabilitation program “Auf die Beine”. | Treatment evaluation | Describing validity in CP (citing CP study) |  |
|  | Li et al.^74^ | 2022 | China | Prospective observational study | Congenital neural tube defect with lipomyelomeningocele | 85 |  | Study group: 0.79 (0.11) months  Control group: 1.34 (0.18) months | GMFM | To evaluate change in gross motor function after surgery. | Treatment evaluation | NA (citing CP study) |  |
|  | Geva-Dayan et al.^75^ | 2010 | Israel | Retrospective review study | Hereditary Spastic Paraparesis (HSP) | 12 |  | 4.8 (2.5); 0.5–8.2 years | GMFM | To report our experience with botulinum toxin type A injections to the lower limbs. | Treatment evaluation | NA (citing Russell 1989 study) |  |
|  | Icagasioglu et al.^90^ | 2014 | Turkey | Case series | HSP | 2 |  | 4 and 6 years | GMFM-88 Total (raw) | To report the effectiveness of Botulinum Toxin type A injections and rehabilitation programs. | Treatment evaluation | NA (no citing) |  |
|  | Sharma et al.^83^ | 2016 | Canada | Retrospective review study | HSP | 4 |  | 3, 7, 6 and 18 years | GMFM-88 A-E (%)  GMFM-88 Total (%) | To determine the outcomes for children who underwent selective dorsal rhizotomy. | Treatment evaluation | NA (no citing) |  |
|  | Grootveld et al.^38^ | 2016 | Netherlands | Case report | HSP | 3 |  | 12, 6, and 7 years | GMFM-66 | To report is to record sudden falls as a persistent complication of SDR and draw attention to this complication. | Natural history and disease progression | NA (no citing) |  |
|  | İpek et al.^91^ | 2017 | Turkey | Case report | Joubert Syndrome | 1 |  | 19 months | GMFM-88 A-E (raw)  GMFM-88 Total (raw) | To report the efficiency of the physiotherapy and rehabilitation program. | Treatment evaluation | NA (citing CP and Russell study) |  |
|  | Stark et al.^39^ | 2015 | Germany | Retrospective analysis | Meningomyelocele and Spina bifida | 66 | 27 | 8.71 (4.70) years | GMFM-66  GMFM-88 D-E (%) | To determine the effect of a vibration-assisted neuromuscular rehabilitation on motor function. | Treatment evaluation | Describing measurement properties other than CP (citing Linder-Lucht and Ruck-Gibis study and user’s manual).  “Meanwhile, the GMFM has been  validated for traumatic brain injury, Down syndrome, and osteogenesis imperfecta.” |  |
|  | Aizawa et al.^92^ | 2017 | Brazil | Randomized controlled trial | Myelomeningocele | 12 |  | Intervention group 18.2 (15.6) months  Control group 18.3 (12.4) months | GMFM-88 A-E (%)  GMFM-88 Total (%) | To validate a physical therapy program based on reflex stimulation. | Treatment evaluation | Acknowledging lack of validation despite widespread use and describing measurement properties other than CP (citing non-measurement study other than CP).  “Although the use of the Gross Motor Function Measure in children with spinal dysraphism has not been validated, it is the most-used scale in the literature. It has been used to measure the motor function of children with cerebral palsy, Down syndrome and spinal cord diseases” |  |
|  | Luz et al.^93^ | 2017 | Brazil | Cross-sectional study | Myelomeningocele | 47 |  | Median 10; 5–20 years | GMFM-88 Total (%) | To investigate the relationships between motor function and other variables in myelomeningocele patients. | Clinical characterization | NA (citing Russell study). |  |
|  | Dekopov et al.^94^ | 2013 | Russia | Prospective study | Spine maldevelopment | 18 | 1 | 8 years^c^ | GMFM-88 Total (%) | To estimate the effectiveness of selective peripheral neurotomy. | Treatment evaluation | NA (no citing). |  |
| Central Nervous System Disorders (Acquired/Progressive) | Beretta et al.^95^ | 2015 | Italy | Within-subjects study | Acquired brain injury (ABI) | 34 |  | Experimental group: 13 years 11 months (5 years 5 months)  Control group: NA | GMFM-88 A-E (%)  GMFM-88 Total (raw & %) | The objective is to investigate the functional changes induced by the combined administration of robotic-aided gait training and physical therapy. | Treatment evaluation | Describing measurement properties other than CP (citing Linder-Lucht study).  “Linder-Lucht also demonstrated the reliability of GMFM in the evaluation of functional abilities in children with ABI.” |  |
|  | Kelly et al.^54^ | 2015 | UK | Observational study | ABI | 74 |  | Median 11.3; 0.3–17.3 years | GMFM-88 Total  GMFM-66 | To explore the appropriateness of using the interval-scale version of the Gross Motor Function Measure (GMFM-66) in pediatric acquired brain injury. | Clinical characterization | Describing measurement properties other than CP (citing Linder-Lucht study).  “It has been recommended as an outcome measure after paediatric ABI, although experience of its use in this context is limited. Linder-Lucht et al. validated the GMFM-88 for children with TBI using video analysis as a criterion standard.” |  |
|  | Biffi et al.^96^ | 2017 | Italy | Within-subjects study | ABI | 12 |  | 12.1 (3.8) years | GMFM-88 E (%)  GMFM-88 Total (%) | To evaluate the efficacy of a rehabilitation treatment on a Gait Real-time Analysis Interactive Lab system for the improvement of walking abilities. | Treatment evaluation | Describing measurement properties other than CP (citing Linder-Lucht study).  “Linder-Lucht demonstrated the validity of supporting the use  of the GMFM as an evaluative measure of gross motor function in children with Traumatic Brain Injury.” |  |
|  | Beretta et al.^97^ | 2018 | Italy | Prospective two-cohorts study | ABI | 41 |  | Experimental group: 11.2 years  Control group: 11.9 years | GMFM-88 A-E (%)  GMFM-88 Total (raw & %) | To compare conventional physical therapy based treatment with a combined program of Robotically-Driven Orthoses training. | Treatment evaluation | Describing measurement properties other than CP (citing Russell study and Linder-Lucht et al.).  “Linder-Lucht demonstrated the validity of GMFM in the evaluation of gross motor function also in children with ABI.” |  |
|  | Beretta et al.^98^ | 2020 | Italy | Cohort, pretest-posttest retrospective study | ABI | 182 | 110 | 10.8 (4.1) years^c^ | GMFM-88 D-E (raw)  GMFM-88 Total (raw) | To evaluate the effect of robotic-aided gait training and physical therapy. | Treatment evaluation | NA (citing Russell and Linder-Lucht study). |  |
|  | Biffi et al.^99^ | 2021 | Italy | Propensity score matched cohort study | ABI | 30 |  | Robot assisted gait training (RAGT): 15.0 (10.6) years  Virtual reality plus treadmill training (VRTT): 11.3 (5.2) years | GMFM-88 D-E (%)  GMFM-88 Total (%) | To compare the effectiveness of RAGT and VRTT. | Treatment evaluation | Describing measurement properties other than CP (citing Linder-Lucht study).  “The validity of GMFM-88 in the evaluation of gross motor function in children with ABI has been previously demonstrated.” |  |
|  | Ryan et al.^100^ | 2022 | Canada | Retrospective observational study | ABI  (subtypes)  Stroke  Brain tumour  Traumatic brain injury  Epilepsy  Post-infectious  Neuroinflammatory  Hypoxia | 266 |  | 11 years 9 months (3 years 9 months) | GMFM-88 Total (%) | To obtain clinical estimates of gross motor change in school-aged children with subacute ABI, explore differences in gross motor change based on age, sex, diagnosis and baseline motor function, and summarize physiotherapy Goal Attainment Scaling goal areas relative to the child's gross motor function. | Clinical characterization | Describing measurement properties other than CP (citing Linder-Lucht study).  “The Gross Motor Function Measure  (GMFM­88) contains five domains (lying and rolling; sitting; crawling and kneeling; standing; walking, running, and jumping), and is validated for children with ABI.” |  |
|  | Forsyth et al.^55^ | 2022 | United Kingdom | Observational study | ABI | 158 |  | Median 13.1; IQR 7.1–17.9 years | GMFM-66 | To examine relationships between functional outcomes. | Clinical characterization | Describing measurement properties other than CP (citing non-measurement property study other than CP).  “We have previously demonstrated the validity of the GMFM in the severe ABI population.” |  |
|  | Liem et al.^58^ | 2020 | Vietnam | Case series | ABI due to non-fatal drowning | 5 |  | 26, 36, 63, 37 and 25months | GMFM-88 | To evaluate the efficacy of bone marrow-derived mononuclear cell therapy. | Treatment evaluation | NA (citing CP study). |  |
|  | Peri et al.^101^ | 2019 | Italy | Within-subjects study | ABI with ataxia | 11 |  | 16.0 (5.0); 9–27 years | GMFM-88 D-E (%)  GMFM-88 Total (%) | To assess changes in locomotion and balance in adolescents affected by ataxia secondary to acquired brain injury aſter a rehabilitation treatment with physiotherapy and the Gait Real-time Analysis Interactive Lab, an immersive virtual reality platform. | Treatment evaluation | NA (citing Linder-Lucht study). |  |
|  | Panzeri et al.^102^ | 2022 | Italy | Single series study | ABI-related hemiparesis | 6 |  | 9.8, 18.3, 7.8, 15.9, 12.8 and 14.3 years | GMFM-88 D-E (%)  GMFM-88 Total (%) | To evaluate how a personalized program using visual biofeedback in a virtual reality setting can impact on gross motor function of subjects with ABI-related hemiparesis. | Treatment evaluation | Describing measurement property other than CP (citing Linder-Lucht study).  “The reliability of GMFM in patients with ABI has been reported in the  literature” |  |
|  | Molteni et al.^103^ | 2015 | Italy | Within-subjects study | Acquired Hemiplegia  (subtypes)  Traumatic brain injury  Brain tumor  Ictus  Encephalitis  Arachnoidal cyst  Cerebral angiopathy | 15 |  | Experimental group: 10.2 (4.1) months  Control group: 10.3 (7.7) months | GMFM-88 A-E (%)  GMFM-88 Total (raw & %) | To evaluate gross motor function before and after Robotic-Aided Gait Training, and compare with control. | Treatment evaluation | Describing measurement property other than CP (citing Linder-Lucht study).  “Linder-Lucht also demonstrated the reliability of GMFM in the evaluation of functional abilities in children with acquired brain injuries.” |  |
|  | Masoud et al.^104^ | 2017 | United States | Cross-sectional study | Alternating hemiplegia of childhood (AHC) | 23 | 6 | 15 months–43 years | GMFM-88 A-E (%)  GMFM-88 Total (%) | To characterize the profile of motor function domains, and to investigate interrelationships that these domains may have with each other and with age. | Clinical characterization | Describing measurement property other than CP (citing Russell and Linder-Lucht study).  “it has also been used in patients with traumatic brain injuries, and for CP in adult patients.” |  |
|  | Lardinois et al.^105^ | 2019 | United States | Case report | AHC | 1 |  | 14 months | GMFM-88 A-E (%)  GMFM-88 Total (%) | To examine gross motor function. | Clinical characterization | NA (citing CP study) |  |
|  | Nguyen Thanh et al.^106^ | 2022 | Vietnam | Case report | Anti-N-methyl-d-aspartate (NMDA) receptor encephalitis | 1 |  | 65 months | GMFM-88 A-E (raw)  GMFM-88 Total (raw) | To report the efficacy of allogeneic umbilical cord-derived mesenchymal stem/stromal cells for autoimmune encephalitis on gross motor function. | Treatment evaluation | NA (citing GMFM score sheet) |  |
|  | Liu et al.^59^ | 2016 | China | Randomized controlled trial | Brain Damage Syndrome | 62 |  | Comprehensive group: 4.46 (0.80) years  Control group: 4.46 (0.73) years | GMFM-88 A&B  GMFM-88 | To assess whether the intelligence seven needle therapy would improve patients' gross motor function. | Treatment evaluation | NA (no citing) |  |
|  | Shi et al.^107^ | 2019 | (China) | Quasi-experimental comparative study | Brain injury | 100 |  | NA | GMFM-88 Total (raw) | To evaluate head control ability recovery. | Treatment evaluation |  |  |
|  | Lee et al.^108^ | 2021 | South Korea | Single‐subject experimental study | Cerebellar ataxia (after brainstem glioma surgery) | 1 |  | 5 years | GMFM-88 Total (%) | To investigate the effects of problem‐based task training on motor function. | Treatment evaluation | Describing validity in CP (citing CP study). |  |
|  | Tustin et al.^109^ | 2019 | United Kingdom | Prospective observational study | Childhood-onset dystonia | 60 |  | Median 11 years 1 month; 7 years 4 months–14 years 8 months | GMFM-88 Total (%) | To examine the impact of deep brain stimulation on gross motor function. | Treatment evaluation | NA (citing CP study) |  |
|  | Eggink et al.^110^ | 2019 | Netherlands | Prospective observational cross-sectional study | Dystonia | 60 |  | 13.6 (5.9) years | GMFM-88 Total (%) | To investigate the relationship between motor and non-motor symptoms, and health-related quality of life. | Clinical characterization | NA (citing CP study) |  |
|  | van Empelen et al.^111^ | 2004 | Netherlands | Prospective observational study | Epilepsy (after functional hemispherectomy)  (subtypes)  Congenital middle cerebral artery infarction  Sturge–Weber syndrome  Rasmussen encephalitis  Hemiplegia, hemiconvulsions, epilepsy syndrome | 12 |  | 5.9; 0.3–11.1 years | GMFM-88 A-E (%)  GMFM-88 Total (%) | To assess gross motor functioning before and three times over a 2-year period after hemispherectomy. | Treatment evaluation | Describing validity in CP (citing CP study). |  |
|  | van Empelen et al.^112^ | 2005 | Netherlands | Prospective observational study | Epilepsy (pharmacologically untreatable)  (subtypes)  Rasmussen encephalitis  Sturge–Weber syndrome  Cerebral tumour  Unclear | 37 | 9 | 5 months–10 years 3 months | GMFM-88 Total (%) | To assess the impact of epilepsy surgery on motor performance. | Treatment evaluation | Describing validity in CP (citing CP study). |  |
|  | van der Kolk et al.^113^ | 2013 | Netherlands | Retrospective consecutive cohort study | Epilepsy (after functional hemispherectomy)  (subtypes)  Hemimegalencephaly  Cortical dysplasia  Interhemispheric cystic malformation, bilateral colpocephaly and delayed myelination  Periventricular hemorrhages  Perinatally acquired ischemic cortico-subcortical lesions  Hemispheric atrophy in the context of hemiconvulsion—hemiplegia—epilepsy syndrome  Rasmussen's encephalitis  Progressive atrophy and calcifications due to Sturge—Weber syndrome | 35 |  | Developmental disorders: 1.53 (1.22) years  Acquired lesions: 5.1 (3.74) years  Progressive disorders: 7.0 (5.05) years | GMFM-88 Total (%) | To assess motor function before hemispherectomy and 24 months after hemispherectomy. | Treatment evaluation | Describing validity in CP (citing CP study and Russell study). |  |
|  | Graveline et al^114^. | 1999 | Canada | Cross-sectional study | Hemidecortication | 18 |  | Group 1: 21–243 months  Group 2: 56–201 months | GMFM-88 A-B (%)  GMFM-88 Total (%) | To determine the levels of gross and fine motor functions in children before and after hemidecortication. | Treatment evaluation | NA (citing CP study). |  |
|  | Petrarca et al.^115^ | 2011 | Italy | Case report | Ischemic stroke | 1 |  | 6 years | GMFM-88 Total (%) | To evaluate gait performance before and after selected treatments. | Treatment evaluation | NA (no citing) |  |
|  | Mann et al.^116^ | 2020 | United States | Single case study,an ABA design | Kernicterus spectrum disorder | 1 |  | 28 months | GMFM-88 A-C (raw & %) | To assess gross motor function before and after treatment. | Treatment evaluation | Acknowledging lack of validation despite widespread use　(citing CP study and user’s manual)  “it is generally accepted that is sensitive to changes in children with other neuromotor impairments.” |  |
|  | Çubukçu et al.^117^ | 2018 | Turkey | Prospective study | Multidrug-Resistant Epilepsy | 62 |  | Median 5.0; 4.0–7.0 years | GMFM-88 A-E (%)  GMFM-88 Total (%) | To investigate the effect of ketogenic diet. | Treatment evaluation | NA (citing CP study) |  |
|  | Smith^40^ | 2014 | United States | Case report | Sydenham chorea | 1 |  | 5 years | GMFM-66 | To assess the patient’s gross motor function and to allow the therapist to track change over time and the response to interventions provided. | Treatment evaluation | Describing measurement properties other than CP and Acknowledging lack of validation despite widespread use (citing Linder-Lucht study)  "Although the GMFM has not been validated for children with Sydenham chorea, the decision was made to use the GMFM as children with CP and traumatic brain injuries can present with similar movement impairments as the child in this case study." |  |
|  | Kuhtz-Buschbeck et al.^118^ | 2003 | Germany | Prospective study | Traumatic brain injury (TBI) | 23 | 20 | Median 9 years 6 months; 4 years 7months–15 years 10 months | GMFM-88 Total (%) | To demonstrate improvement at a 5-month interval of rehabilitation. | Treatment evaluation | Describing validity in CP.  (citing CP study and Russell study). |  |
|  | Beretta et al.^119^ | 2009 | Italy | Prospective study | TBI | 14 |  | 8.7; 4.4–12.1 years | GMFM-88 E (%)  GMFM-88 Total (%) | To determine if there is a correlation between parameters obtained by 3-Dimensional Gait Analysis, clinical assessment, and trauma-related indicators. | Treatment evaluation | Describing measurement properties other than CP (citing Russell and Linder-Lucht study)  “Linder-Lucht demonstrated the validity of supporting the use of the GMFM as an evaluative measure of gross motor function in children with TBI.” |  |
|  | Cimolin et al.^120^ | 2012 | Italy | Pre-post study | TBI | 10 |  | 8.8–12.9 years | GMFM-88 Total (%) | To evaluate the effectiveness of constraint-induced movement therapy. | Treatment evaluation | Describing measurement properties other than CP (citing Russell and Linder-Lucht study)  “Linder-Lucht demonstrated the validity of supporting the use of the GMFM as an evaluative measure of gross motor function in children with TBI.” |  |
|  | Ryan et al.^121^ | 2023 | Canada | Single case study | TBI | 1 |  | NA | GMFM-88 D-E (%)  GMFM-88 Total (%) | To describe gross motor progress. | Treatment evaluation | Describing measurement properties other than CP (citing Linder-Lucht and Storm study)  “The GMFM-88 is a reliable and responsive outcome measure that has been validated for children with ABI.”  “The minimum clinically important difference for the GMFM-88 and COPM are 5% and 2 points, respectively.” |  |
|  | Son et al.^122^ | 2018 | South Korea | Case report | White matter injury | 1 |  | 5 months | GMFM-88 A-C (raw & %) | To know the motor function prognosis. | Treatment evaluation | NA (no citing) |  |
| Neurological Disorders | Civil et al.^123^ | 2023 | United States | Case report | Arthrogryposis multiplex congenital  Spinal stroke | 2 |  | 4 and 13 years | GMFM-88 D-E (%)  GMFM-88 Total (%) | To evaluate via functional measures in barefoot, ankle-foot orthosis, and soft ankle support. | Treatment evaluation | NA (citing CP study) |  |
|  | Grunt et al.^41^ | 2010 | Netherlands | Retrospective study | Congenital hydrocephalus  Spinal process  HIV-Encephalo(myelo)pathy  Laurence moon syndrome | 36 | 5 | 8, 10, 6, 8, and 3 years^c^ | GMFM-66 | To identify possible relationships between the magnetic resonance imaging findings with the level of gross motor function of the patient before selective dorsal rhizotomy (SDR) and the change in functioning after SDR. | Treatment evaluation | Describing validity in CP (citing Russell study) |  |
|  | Jensen-Willett et al.^42^ | 2022 | United States | Case Series | Dravet syndrome  CDKL5 deficiency disorder  Infantile spasms  Seizure disorder  Sturge-Weber syndrome  Prader-Willi syndrome  Hydrocephalus | 13 | 7 | 10.9; 8-17 months^c^ | GMFM-66-IS | To examine the response to Sitting Together and Reaching to Play intervention. | Treatment evaluation | NA (citing CP study) |  |
|  | Çelik et al.^124^ | 2021 | Turkey | Cross-sectional descriptive research | Obstetric brachial plexus palsy (OBPP) | 106 |  | Type 2a: 13.07 (2.84) months  Type 2b: 13.44 (2.38) months  Type 3: 12.67 (2.87) months  Type 4: 13.47 (2.80) months | GMFM-88 B (%) | To assess sitting skills and investigate the effect of these skills on upper extremity function. | Treatment evaluation | Describing measurement properties other than CP (citing Russell 1998, Linder-Lucht and Ruck-Gibis study)  “it has been validated for populations living with neurological problems such as Down’s syndrome, traumatic brain injury, and osteogenesis imperfecta.” |  |
|  | Çelik et al.^125^ | 2021 | Turkey | Cross-sectional descriptive research | OBPP | 106 |  | Type 2a: 13.07 (2.84) months  Type 2b: 13.44 (2.38) months  Type 3: 12.67 (2.87) months  Type 4: 13.47 (2.80) months | GMFM-88 A (%) | To investigate the gross motor functions and the relationship between these developmental skills and upper extremity skill quality. | Treatment evaluation | Describing measurement properties other than CP (citing Russell 1998, Linder-Lucht and Ruck-Gibis study)  “It has also been validated for populations living with neurological problems, including Down’s syndrome traumatic brain injury and osteogenesis imperfecta.” |  |
| Infectious Disease Related Conditions | Frota et al.^126^ | 2020 | Brazil | Retrospective study | Congenital Zika syndrome (CZS) | 50 | 46 | GMFM-88 evaluation at 24 months^c^ | GMFM-88 A-E (raw)  GMFM-88 Total (raw & %) | To describe the gross motor function at 24 months of age and to evaluate the association between presence of symptoms, comorbidities and gross motor development. | Natural history and disease progression | NA (citing CP study) |  |
|  | Melo et al.^127^ | 2020 | Brazil | A cross-sectional observational study | CZS | 59 |  | 14.7 (3.9); 5–29 months | GMFM-88 A-E (raw & %)  GMFM-88 Total (raw & %) | To evaluate gross motor function and associated factors. | Natural history and disease progression | NA (citing CP and Russell study) |  |
|  | Massetti et al.^128^ | 2020 | Brazil | Cross-sectional, prospective, and descriptive study | CZS | 31 |  | 6–18 months | GMFM-88 A (raw & %) | To describe gross motor performance, and compare the clinical outcomes of infants infected in the first trimester and in the second trimester of pregnancy, and investigate possible correlations between outcomes. | Natural history and disease progression | NA (citing CP study) |  |
|  | Gama et al.^129^ | 2021 | Brazil | Retrospective Cohort Study | CZS | 7 |  | 14–18 months | GMFM-88 A-B (raw)  GMFM-88 Total (raw) | To investigate the effect of intensive physiotherapy training. | Treatment evaluation | NA (citing CP and Russell study) |  |
|  | Takahasi et al.^43^ | 2021 | Brazil | A prospective cohort | CZS  (subtypes)  Confirmed CZS  Presumed CZS | 100 |  | Confirmed CZS: Median 25.6; IQR 5.1 months  Presumed CZS: Median 25.5; IQR 5.8 months | GMFM-66  GMFM-88 Total (%) | To evaluate gross motor function in CZS children aged up to 3 years, and its associated factors and changes in a minimum interval of 6 months. | Natural history and disease progression | NA (citing CP and Russell study) |  |
|  | Muller et al.^60^ | 2022 | Brazil | Prospective interventional cohort study (Within-subjects study) | CZS | 30 |  | 30.1 (3); 21–35 months | GMFM-88 A-E  GMFM-88 Total | To evaluate the effects of Neurodevelopmental Treatment (NDT) on motor function in children with CZV. | Treatment evaluation | NA (citing Russell study) |  |
|  | Ribeiro et al.^130^ | 2022 | Brazil | Cross-sectional Study | CZS | 72 |  | 13.9 (4.4); 7–24 months | GMFM-88 A-E (%)  GMFM-88 Total (%) | To assess gross motor function in children with CZS. | Natural history and disease progression | NA (citing CP study) |  |
|  | Gama et al.^131^ | 2023 | Brazil | Prospective Cohort Study | CZS | 52 |  | 46.07 (3.76) months | GMFM-88 A-B (raw)  GMFM-88 Total (raw) | To describe the impact of social during the COVID-19 pandemic. | Treatment evaluation | NA (citing CP study) |  |
|  | Ventura et al.^44^ | 2020 | Brazil | Prospective observational study | Congenital Zika Virus Syndrome (ZIKV) | 77 |  | Median 4 months | GMFM-66 | To describe how gross motor function develops over time in children exposed to congenital ZIKV infection. | Natural history and disease progression | Describing validity in CP.  (citing CP and Russell study) |  |
|  | Hamanaka et al.^132^ | 2022 | Brazil | Prospective cohort study | ZIKV | 74 |  | Median 13.0; 8–24 months | GMFM-88 Total (%) | To describe the evolution of gross motor function. | Natural history and disease progression | NA (citing CP study) |  |
|  | Gama et al.^45^ | 2023 | Brazil | Case report | ZIKV | 1 |  | 14 months | GMFM-88 A-B (raw)  GMFM-88 Total (raw)  GMFM-66 | To provide a detailed description of the development. | Treatment evaluation | NA (citing CP and Russell study) |  |
|  | Mann et al.^133^ | 2017 | South Africa | Cross-sectional study | HIV encephalopathy (with bilateral lower limb spasticity) | 30 |  | Median 8; IQR 7–11 years | GMFM-88 A-E (%)  GMFM-88 Total (%) | To describe gross motor function, and to investigate the association between age, CD4 percentage, and viral load at initiation of antiretroviral therapy and current gross motor function. | Natural history and disease progression | Describing validity in CP (citing CP study) |  |
|  | Naik et al.^134^ | 2018 | South Africa | Cross-sectional study | HIV encephalopathy (with spastic diplegia) | 33 |  | Non-ambulant group: 8.06 (2.35) years  Ambulant group: 7.53 (2.76) years | GMFM-88 A-E (%)  GMFM-88 Total (%) | To determine whether the clinical presentation of HIV-infected children with spastic diplegia is similar to that of children with cerebral palsy (CP) spastic diplegia who are HIV uninfected with regards to gross motor function. | Natural history and disease progression | Describing validity in CP (citing CP study) |  |
|  | Kunjarkar et al.^135^ | 2022 | India | Case report | Japanese encephalitis | 1 |  | 5 years | GMFM-88 A-B (raw) | To report on the rehabilitation progress of the case. | Treatment evaluation | NA (citing CP study) |  |
|  | Konno et al.^136^ | 2019 | Malawi | Case report | Malaria | 2 |  | 10 and 9 years | GMFM-88 A-E (%)  GMFM-88 Total (%) | To report on treatment to find effective approaches. | Treatment evaluation | NA (no citing) |  |
| Neuromuscular Disorders | Buckon et al.^137^ | 2016 | United States | Prospective multi-site study | Duchenne Muscular Dystrophy (DMD) | 85 |  | 90; 49–180 months | GMFM-88 D-E (raw) | To determine how corticosteroid therapy alters the natural course of the disease. | Treatment evaluation | Describing measurement properties other than CP (citing Russell, Iannaccone 2003, Russell 1998 and Nelson study)  “The GMFM has been shown to be valid, reliable and responsive in children with Down syndrome and spinal muscular atrophy, thus it is reasonable to consider the GMFM as an indicator of changes in muscle function in DMD.” |  |
|  | Buckon et al.^138^ | 2022 | United States | Prospective study | DMD | 85 |  | 93; 49–180 months | GMFM-88 D-E (raw) | To determine the effect of corticosteroid therapy on the rate of decline in gross motor skill. | Treatment evaluation | Describing measurement properties other than CP (citing Russell 1998 and Nelson study)  “It has been reported to be a reliable and valid assessment of change in children with Down syndrome as well as pediatric onset neuromuscular disease such as Spinal Muscular Atrophy (SMA).” |  |
|  | Li et al.^61^ | 2022 | China | Randomized controlled trial | Exercise-induced muscle fatigue | 110 |  | NA | GMFM-88 | To verify the effect of treatments on gross motor function. | Treatment evaluation | NA (no citing). |  |
|  | Murakami et al.^139^ | 2021 | Japan | Open-label, single-arm clinical study (within-subjects study) | Fukuyama congenital muscular dystrophy (FCMD) | 9 |  | 8.10 (2.14); 4.3–10.9 years | GMFM-88 A-E (%)  GMFM-88 Total (%) | To evaluate the efficacy of steroid therapy for restoring motor functions in FCMD patients. | Treatment evaluation | Describing measurement properties other than CP. (citing Sato 2017 and Sato 2020 study)  “it has also been reported to be useful for assessing the motor functions of patients with FCMD” |  |
|  | Sato et al.^140^ | 2021 | Japan | Cross-sectional study | FCMD | 18 | 15 | 6.0; 2–15 years | GMFM-88 (raw) | To investigate the correlation between urinary titin concentration and GMFM score. | Treatment evaluation | NA (citing Sato 2017 study). |  |
|  | Roth et al.^141^ | 2014 | United States | Natural history study (Prospective Observational Study) | Giant Axonal Neuropathy (GAN) | 13 | 12 | 4–22 years | GMFM-88 Total (raw) | To explore the relationship between the presence of curly hair and gross motor function among patients with GAN. | Natural history and disease progression | NA (citing Iannaccone 2002, Iannaccone 2003 study). |  |
|  | Roth et al.^142^ | 2015 | United States | Natural history study (Prospective Observational Study) | GAN | 10 |  | 4–20 years | GMFM-88 Total (raw) | To show change of gross motor function over six months. | Treatment evaluation | NA (citing CP study) |  |
|  | Liu et al.^76^ | 2021 | China | Within-subjects study | Guillain-Barré Syndrome | 62 |  | Experimental group: 9.27 (1.3) years  Control group: 9.31 (1.1) years | GMFM | To investigate the effect of electromyographic biofeedback therapy or physical therapy and occupational therapy on limb mobility. | Treatment evaluation | NA (no citing) |  |
|  | Prater et al.^46^ | 2012 | United States | Retrospective observational study | Infantile Pompe disease | 11 | 7 | 8.0; 5.4–12.0 years | GMFM-66 | To assess gross motor function before and after enzyme replacement therapy with alglucosidase alfa. | Treatment evaluation | NA (citing CP study) |  |
|  | Chien et al.^143^ | 2017 | Taiwan | Within-subjects study | Infantile-onset Pompe disease (IOPD) | 12 |  | 5.6; 2–12 years | GMFM-88 D-E (%) | To investigate whether an adjuvant therapy with albuterol. | Treatment evaluation | NA (no citing) |  |
|  | Chien et al.^62^ | 2020 | Taiwan | Retrospective multicenter observational study | IOPD | 28 |  | Median 8.3; 0.8–17.3 years | GMFM-88 E | To evaluate the efficacy of different enzyme replacement therapy regimens. | Treatment evaluation | NA (no citing) |  |
|  | Winkel et al.^144^ | 2004 | United States | Single-center, open-label pilot study | Pompe disease (PD) | 3 | 2 | 11 and 16 years^c^ | GMFM-88 Total (%) | To evaluate the efficacy of enzyme replacement therapy. | Treatment evaluation | NA (no citing) |  |
|  | Case et al.^47^ | 2015 | United States | Open-label study | PD | 13 |  | 19.8 (21.29); 1.8–60.1 years | GMFM-66 | To examine the efficacy and safety of alternative regimens of alglucosidase alfa. | Treatment evaluation | Describing measurement properties other than CP (citing Russell 1998, Iannaccone 2003 study and non-measurement study other than CP)  “validity in the use of the GMFM has been expanded to additional diagnoses, including spinal muscular atrophy, which has clinical characteristics of weakness that are similar to those of Pompe disease.” |  |
|  | Bar-Yoseph et al.^145^ | 2018 | Israel | Prospective case control study | PD | 5 | 4 | 10, 11, 12 and 16 years^c^ | GMFM-88 Total(%) | To examine the acute effect of enzyme replacement therapy. | Treatment evaluation | NA (citing CP study) |  |
|  | Hahn et al.^146^ | 2018 | United States | Phase IV, open-label, prospective study | PD | 113 | 90 | ≥ 1 year old | GMFM-88 Total (%) | To evaluate 4,000 L recombinant human acid α-glucosidase efficacy/safety. | Treatment evaluation | Describing validity in CP (citing CP study) |  |
|  | Kishnani et al.^147^ | 2019 | United States | Prospective, open-label, single-arm study | PD | 113 | 108 | Infantile-onset PD: 4.4 (3.52); 1.0–15.5 years  Late-onset PD: 6.0 (4.19) 1.1–18.7 years | GMFM-88 Total (%) | To characterize clinical characteristics and genotypes of patients. | Natural history and disease progression | Acknowledging lack of validation despite widespread use (citing non-measurement study other than CP)  “Motor skills were assessed via Gross Motor Function Measure, 88 items (GMFM-88), which has precedent (though not formal validation) for use in Pompe disease.” |  |
|  | Khan et al.^148^ | 2020 | United States | Retrospective study | PD | 11 |  | Median 15.2; 10.2–18.2 years | GMFM-88 Total (%) | To present higher enzyme replacement therapy dose findings. | Treatment evaluation | NA (citing CP study) |  |
|  | Bar-Yoseph et al.^149^ | 2021 | Israel | Prospective and partially retrospective case-control study | PD | 4 |  | 5.5, 6, 10 and 11 years | GMFM-88 E (%)  GMFM-88 Total (%) | To examine the acute effect and long-term of enzyme replacement therapy. | Treatment evaluation | NA (citing CP study) |  |
|  | O'Hagen et al.^150^ | 2007 | United States | Measurement property study | Spinal muscular atrophy (SMA) | 38 |  | Type Ⅱ: Median 5.7; 2.3–32.5 years  Type Ⅲ: Median 9.1; 3.9–45.1 years | GMFM-88 (%)  GMFM-75 (raw) | To develop an expanded version of the Hammersmith Functional Motor Scale allowing for evaluation of ambulatory SMA patients, and to examine concurrent validity. | Natural history and disease progression | Describing measurement properties other than CP (citing Nelson, Iannaccone 2002, and Iannaccone 2003 study)  “The GMFM provides a sensitive measure of gross motor function in children with type II and III SMA.” |  |
|  | Chen et al.^63^ | 2010 | Taiwan | Randomized, double-blind, placebo-controlled trial | SMA | 57 |  | Hydroxyurea group: 16.6 (10.3); 5–41 years  Placebo group: 14.6 (9.2); 6–40 years | GMFM-88 | To evaluate the safety and efficacy of hydroxyurea. | Treatment evaluation | NA (citing Nelson study) |  |
|  | Stratigopoulos et al.^64^ | 2010 | United States | Cross-sectional study | SMA | 88 |  | Male patients < 11 y: 3 years  Male patients ≧ 11 y: 15 years  Female patients Prepubertal: 5 years  Female patients Postpubertal: 20 years | GMFM-88 | To investigate correlation between plastin 3 expression levels in the blood and gross motor function. | Natural history and disease progression | Describing measurement properties other than CP (citing Nelson study)  “The gross motor function measure (GMFM), an 88-item test measuring motor function that has been validated for SMA types II and III,8 was administered to type II and type III SMA patients.” |  |
|  | Bulut et al.^151^ | 2019 | Turkey | Case report | SMA type II | 1 |  | 60 months | GMFM-88 Total (%) | To identify the effects of two different aerobic training methods. | Treatment evaluation | Describing measurement properties other than CP (citing Nelson study)  “The GMFM-88 scale is also a valid and sensitive outcome measure for children with SMA.” |  |
|  | Glanzman et al.^152^ | 2011 | United States | Cross-sectional study | SMA type II and III | 70 |  | 11.4 (9.1) years | GMFM-88  GMFM-75 (raw) | To clarify the validity of the Expanded Hammersmith Functional Motor Scale. | Natural history and disease progression | Describing measurement properties other than CP (citing Nelson study)  “The Gross Motor Function Measure has been demonstrated to be a valid outcome measure for spinal muscular atrophy types II and III and showed good validity whether or not items requiring prone position on the Gross Motor Function Measure were eliminated from the analysis.” |  |
|  | Wang et al.^153^ | 2013 | Taiwan | Prospective Observational Study | SMA type II and III | 56 |  | 16.0 (9.9); 5–41 years | GMFM-88 A-E (%)  GMFM-88 Total (%) | To examine the correlations between change scores on the Manual Muscle Test for muscle strength and the GMFM for motor function. | Treatment evaluation | Describing measurement properties other than CP (citing Nelson study)  “the Gross Motor Function Measure  (GMFM), has been demonstrated to be a valid and sensitive tool to represent motor function in individuals with SMA.” |  |
|  | Stark et al.^56^ | 2018 | Germany | retrospective observational study | SMA type II and III | 38 | 16 | 4.64 (1.95) years | GMFM-66 | To determine the effect of a vibration-assisted neuromuscular rehabilitation on motor function. | Treatment evaluation | Describing measurement properties other than CP (citing Nelson and Iannaccone 2002 study)  “It contains all motor tasks typically seen in children with spinal muscular  atrophy types II and III. The reliability of the Gross Motor Function Measurement 88 was tested in patients with spinal muscular atrophy.” |  |
|  | Kaufmann et al.^154^ | 2011 | United States | Longitudinal, observational study | SMA type II or III | 65 |  | 11.2 (9.1) years | GMFM-88 Total (%) | To report the results of a prospective multicenter study to follow the clinical evolution. | Treatment evaluation | Describing measurement properties other than CP (citing Nelson study)  “The GMFM is another standardized instrument originally designed to measure change in gross motor function over time in children with cerebral palsy and later validated for SMA.” |  |
|  | Salem et al.^155^ | 2010 | United States | Case report | SMA type III | 1 |  | 34 months | GMFM-88 A-E (raw & %)  GMFM-88 Total (%) | To evaluate changes in motor function by an aquatic therapy program. | Treatment evaluation | Describing measurement properties other than CP (citing Nelson and Iannaccone 2003 study)  “The GMFM-88 was used in this case report because studies that examined the reliability and validity of GMFM in children with SMA used the GMFM-88. GMFM includes items that are responsive to change in children with type III SMA.” |  |
|  | Wong et al.^65^ | 2007 | United States | Randomized, double-blind, placebo-controlled trial | SMA | 55 |  | 2–18 years | GMFM-88 Total | To assess functional strength and motor function. | Treatment evaluation | NA (citing Iannaccone 2002 and Iannaccone 2003 study)  “All equipment and methods have been previously described.” |  |
| Genetic and Chromosomal Disorders | Schreiber^156^ | 2004 | United States | Case report | 18p- | 1 |  | 31 months | GMFM-88 A-E (%)  GMFM-88 Total (%) | To measure motor performance. | Treatment evaluation | Acknowledging lack of validation despite widespread use (citing CP study)  “I chose the GMFM for its applicability and ease of administration. The measurements must be interpreted cautiously, however, because the reliability and validity of the GMFM have not been established for children with 18p-.” |  |
|  | Adang et al.^157^ | 2020 | United States | Retrospective natural history study | Aicardi Goutières Syndrome (AGS) | 61 | 24 | 28.1; 0–186 months | GMFM-88 Total (%) | To compare GMFM-88 and AGS scale. | Natural history and disease progression | NA (citing CP study) |  |
|  | Cattalini et al.^158^ | 2021 | Italy | Case report | AGS | 1 |  | 5 years | GMFM-88 A-E (%)  GMFM-88 Total (%) | To describe the treatment response of the Janus kinase inhibitor ruxolitinib. | Treatment evaluation | NA (no citing) |  |
|  | Dan et al.^82^ | 2001 | Belgium | Comparative study | Angelman Syndrome (AS) | 10 |  | 9.5 (2.1); 7–13 years | GMFM | To assess and compare functional limitations between different patient groups. | Treatment evaluation | NA (citing Russell study) |  |
|  | Kara et al.^159^ | 2010 | Turkey | Case report | AS | 1 |  | 6 months | GMFM-88 Total (%) | To investigate the benefits of physiotherapy programme. | Treatment evaluation | NA (citing CP study) |  |
|  | Aghakhanyan et al.^160^ | 2016 | Italy | Cross-sectional study | AS | 16 |  | 7.7 (3.6) years | GMFM-88 A-E (%) | Relationship between grey matter topography and GMFM-88. | Clinical characterization | Acknowledging lack of validation despite widespread use (citing non-measurement study other than CP)  “The GMFM-88 has been already reported to be valid measures in patients with AS syndrome.” |  |
|  | Micheletti et al.^161^ | 2016 | Italy | Cross-sectional study | AS | 10 |  | 7 years 1 month (1 year 7 months); 5years 2months–11years | GMFM-88 A-E (%)  GMFM-88 Total (%) | To attempt at providing a detailed definition of neurodevelopmental profile. | Natural history and disease progression | NA (citing CP study) |  |
|  | Han et al.^77^ | 2022 | Korea | Case report | AS | 1 |  | 15 years | GMFM | To investigate the effects of robot interactive gait training. | Treatment evaluation | NA (citing CP and Russell study) |  |
|  | Steinbrücker et al.^162^ | 2023 | Australia | Case report | Ataxia telangiectasia (AT) | 1 |  | 3 years 4 months | GMFM-88 A-E (raw)  GMFM-88 Total (raw) | To monitor treatment effect on gross motor function. | Treatment evaluation | NA (citing CP study) |  |
|  | Unes et al.^163^ | 2021 | Turkey | Case report | AT | 1 |  | 9 years | GMFM-88 A-E (%)  GMFM-88 Total (%) | To investigate the benefits of a 12-week physical therapy program to motor skills | Treatment evaluation | Describing measurement properties other than CP (no citing)  “The GMFM has shown to be a valid  and reliable tool for children with motor disability.” |  |
|  | Ardolino^57^ | 2017 | United States | Case series | Chromosome 3 deletion and agenesis of the callosum | 2 | 1 | 24 months^c^ | GMFM-66 | To evaluate gross motor function outcomes after dynamic weight-bearing. | Treatment evaluation | Describing validity in CP (citing Russell 1998 study) |  |
|  | Palisano et al.^164^ | 2001 | Canada | Cross-sectional study | Down syndrome (DS) | 121 |  | 28.9 (20.7); 1.7–72 months | GMFM-88 Total (%) | To create gross motor function growth curves for children with DS, and to estimate the probability that motor functions are achieved by different ages. | Natural history and disease progression | Describing measurement properties other than CP (citing Russell 1989 and Russell 1998 study)  “The total score varies from 0 to 100. The GMFM is reliable, valid, and responsive (ability to measure change) for children with cerebral palsy (CP) and DS.” |  |
|  | Martin^165^ | 2004 | United States | Within-subjects study | DS | 14 |  | 5 years 10 months (17.2 months); 3years 6months–8years | GMFM-88 D-E (%) | To explore the effects of a flexible supramalleolar orthosis on gross motor function. | Treatment evaluation | Describing measurement properties other than CP (citing Russell 1998 study)  “Russell et al. established the test–retest reliability (within 2 weeks) of the GMFM for a sample of children with Down syndrome” |  |
|  | LaForme Fiss et al.^166^ | 2009 | United States | Within-subjects study | DS | 10 |  | Treatment: 16.8 (6.8) months  Control: 20 (3.5) months | GMFM-88 A-E (%)  GMFM-88 Total (%) | To evaluate motor performance of each child, and to compare sensorimotor groups intervention and individual intervention. | Treatment evaluation | NA (citing CP study) |  |
|  | van den Heuvel et al.^167^ | 2009 | Netherlands | Measurement property study | DS | 41 |  | ≤ 2 years: 64.4 (26.6) weeks  2 to 3 years: 130.2 (15.8) weeks | GMFM-88 Total (%) | To examine external responsiveness of the Test of Basic Motor Skills. | Treatment evaluation | Describing measurement properties other than CP (citing CP and Russell 1998 study)  “Russell and colleagues tested the reliability and responsiveness of the GMFM for children with DS in Canada.” |  |
|  | Champagne et al.^168^ | 2010 | Canada | Case reports | DS | 2 |  | 28 and 37 months | GMFM-88 A-E (%) | To describe the impact of an 11-week hippotherapy program. | Treatment evaluation | Describing measurement properties other than CP (citing Russell 1998 study)  “The Gross Motor Function Measure (GMFM) is recognized as being reliable and sensitive enough to quantify gross motor changes in children with Down syndrome.” |  |
|  | Karimi et al.^66^ | 2010 | Iran | Prospective, single blinded study | DS | 18 |  | 6–18 months | GMFM-88 | To compare the effects of simultaneous sensory stimulations and current occupational therapy approaches. | Treatment evaluation | Describing measurement properties other than CP (citing CP study)  “first introduced by Russell and colleagues in 1993 to evaluate the motor function of the children with Down syndrome or cerebral palsy” |  |
|  | Looper et al.^169^ | 2010 | United States | Randomized controlled trial | DS | 17 |  | Experimental group: 6.9 (3.6) months  Control group: 8.9 (2.9) months | GMFM-88 B-E  GMFM-88 Total (raw) | To compare the gross motor skill acquisition between early orthosis use in combination with treadmill training and treadmill training alone. | Treatment evaluation | Describing measurement properties other than CP (citing Russell 1998 study)  “The test was developed for  children with cerebral palsy but has  since been validated for children  with DS.” |  |
|  | Tamminga et al.^170^ | 2012 | United States | Prospective single-subject, alternating treatment design | DS | 2 |  | 24 and 19 months | GMFM-88 D-E (raw) | To examine the effects of 2 types of supramalleolar orthoses on gross motor skills. | Treatment evaluation | Describing measurement properties other than CP (citing Russell 1998 study)  “The test-retest reliability (within 2 weeks) of the GMFM with a sample of children with DS has been established with an intraclass correlation coefficient of 0.98 for dimension D and 0.95 for dimension E.” |  |
|  | Wang et al.^171^ | 2012 | Taiwan | Within-subjects study | DS | 23 |  | 14.4 (2.8); 8.4–19.0 years | GMFM-88 D-E (%) | To investigate the relationships between task-oriented postural control and motor ability. | Clinical characterization | Describing measurement properties other than CP (citing Russell 1998 study)  “GMFM was determined to have appropriate psychometric properties of measuring gross motor function for children with DS.” |  |
|  | Malak et al.^172^ | 2013 | Poland | Cross-sectional study | DS | 70 |  | 4 years 6 months | GMFM-88 Total (%) | To assess the global motor functions and body balance in relation to age and mental development. | Natural history and disease progression | Describing measurement properties other than CP (citing CP study and non-measurement study other than CP)  “At present, 88 items may be used to assess children with DS.” |  |
|  | Nicolini-Panisson et al.^173^ | 2014 | Brazil | Cross-sectional observational study | DS | 40 |  | 10.6 (4.4) years | GMFM-88 D-E (%)  GMFM-88 Total (%) | To examine the Timed Up and Go test validity in relation to GMFM in a sample of individuals with DS. | Clinical characterization | Describing measurement properties other than CP (citing Russell 1998 study)  “it has been used in studies of children with Down syndrome, and it has already been validated for this use.” |  |
|  | Malak et al.^174^ | 2015 | Poland | Cross-sectional study | DS | 79 |  | 6 years 3 months (4 years 6 months) | GMFM-88 E (%)  GMFM-88 Total (%) | To examine gross motor function and determine which are significantly delayed in DS. | Natural history and disease progression | NA (citing CP study) |  |
|  | Beqaj et al.^175^ | 2018 | Republic of Kosovo | Cross-sectional study | DS | 44 |  | Female 149.69 (61.07); 39–227 months  Male 140.67 (48.03); 59–227 months | GMFM-88 Total (%) | To investigate the relationship between physical, motor, and functional domains. | Natural history and disease progression | Describing measurement properties other than CP (citing Russell 1998 study)  “it has also been validated for use with children with DS” |  |
|  | McGuire et al.^176^ | 2019 | United States | 1-group pretest/posttest design (within-subjects study) | DS | 6 |  | 4–3 years | GMFM-88 D-E (%) | To measure effects of an adapted dance program on motor abilities. | Treatment evaluation | Describing measurement properties other than CP (citing Russell 1998 study)  “The GMFM-88 has been validated and shown to be responsive to clinically meaningful change in children with DS” |  |
|  | Mizukami et al.^73^ | 2019 | Japan | Cross-sectional study | DS | 75 |  | 20.9 (7.4) months | GMFM-88 | To identify factors preventing the acquisition of masticatory function. | Treatment evaluation | NA (citing CP and Russell 1998 study) |  |
|  | Flores et al.^177^ | 2020 | United States | Cross-sectional study | DS | 18 |  | 13.67 (5.31); 6–23 months | GMFM-88 B (raw)  GMFM-88 Total (raw) | To determine concurrent validity of the Segmental Assessment of Trunk Control. | Treatment evaluation | Describing measurement properties other than CP (citing CP and Russell 1998 study)  “can be used for children with DS younger than 6 years” |  |
|  | Habib-hasan et al.^178^ | 2020 | Pakistan | Preliminary feasibility study | DS | 48 |  | 16.2 (10.8) months | GMFM-88 Total (%) | To evaluate the feasibility and effectiveness of the Parent Empowerment Program to help caretakers deliver a home program. | Treatment evaluation | Describing measurement properties other than CP (citing CP study and non-measurement study other than CP)  “This is a universally accepted scoring system for children with disabilities”  “international gold standard to measure gross motor capacity in children with DS or CP” |  |
|  | Moriello et al.^179^ | 2020 | United States | Case Series | DS | 4 |  | 38, 38, 53, 56 months | GMFM-88 D-E (%)  GMFM-88 Total (%) | To document gross motor function, gait parameter, and bladder control outcomes following physical therapy incorporating hippotherapy in children with DS. | Treatment evaluation | Describing measurement properties other than CP (citing non-measurement study other than CP)  “test-retest reliability and interrater reliability range from good to excellent (ICC = 0.62–0.98) and ICC = 0.73–0.98, respectively) in children with DS.” |  |
|  | Milligan et al.^180^ | 2022 | United States | Within-subjects study | DS | 20 |  | 10 months–5 years | GMFM-88 Total (raw) | To describe improvements in gross motor skills in a group of children with DS following a group aquatic therapy and an aquatic exercise program. | Treatment evaluation | Describing measurement properties other than CP (citing Russell 1998 study)  “The GMFM-88 has been shown to be a reliable and valid tool at predicting gross motor change in children with DS between the ages of 5 months and those with motor skills below the typical 5-year level.” |  |
|  | Pauletti Oliveira et al.^181^ | 2023 | Brazil | Cross-sectional Study | DS | 21 |  | 10.3 (2.3); 7–14 years | GMFM-88 D-E (raw) | To investigate factors related to postural sway during sit-to-stand movement in children and adolescents with DS. | Clinical characterization | Describing measurement properties other than CP (citing Russell 1998 study)  “developed by Russell et al. (1998); it evaluates gross motor function in children with cerebral palsy and with DS” |  |
|  | Belluscio et al.^182^ | 2019 | Italy | Cross-sectional study | DS  Prader-Willi syndrome (PWS) | 26 |  | DS: 6.63 years  PWS: 5.30 years | GMFM-88 A-E (%)  GMFM-88 Total (%) | To compare the gross motor capacity of DS and PWS. | Natural history and disease progression | Describing measurement properties other than CP (citing measurement properties other than CP study)  “This scale has been widely used to measure functional mobility in children with DS” |  |
|  | Looper et al.^67^ | 2020 | United States | Nonrandomized evaluation study (within-subjects study) | DS or mosaic trisomy 21 | 20 | 17 | 4.8 (2.4) years | GMFM-88 E | To determine if supramalleolar orthosis use improved activity level skill. | Treatment evaluation | Describing measurement properties other than CP (citing Russell 1998 study)  “This is a standardized criterion referenced test that has been validated for use in children with DS.” |  |
|  | Hong et al.^183^ | 2017 | Korea | Within-subjects study | Genetic abnormalities  Developmental delays | 103 | 23 | 32.62 (14.56) months | GMFM-88 Total (%) | To explore the short term effects of comprehensive intensive rehabilitative therapy. | Treatment evaluation | Describing validity in CP (citing CP study) |  |
|  | Salazar et al.^184^ | 2021 | United States | Cross-sectional study | HNRNPH2-related disorder | 17 |  | 11.2 (8.9); 2.7–37.1 years | GMFM-88 Total (raw & %) | To describe the gross motor function and determine the associations between clinician-measured motor function and caregiver-reported mobility scores. | Clinical characterization | Acknowledging lack of validation despite widespread use (citing the user’s manual and non-measurement study other than CP)  “Items from the GMFM-88 have been used to develop scales to measure motor function in　neurodevelopmental disorders such as Rett syndrome.” |  |
|  | Malloy et al.^185^ | 2023 | United States | Cross-sectional study | Hutchinson-Gilford Progeria Syndrome | 38 | 20 | 6.7 (3.8); 2.1–17.5 years | GMFM-88 A-E (raw & %)  GMFM-88 Total (raw & %) | To describe impairment in gross motor function, and evaluate the association between GMFM scores and other measures. | Clinical characterization | Describing measurement properties other than CP (citing Ruck-Gibis study  “The GMFM-88, an activity level measure, has been shown to be valid and reliable in measuring gross motor function across several conditions.” |  |
|  | Kara et al.^186^ | 2010 | Turkey | Case report | Incontinentia pigmenti (Bloch–Sulzberger disease) | 1 |  | 4 months | GMFM-88 Total (%) | To demonstrate the effectiveness of early physiotherapy. | Treatment evaluation | NA (citing CP study) |  |
|  | Paula et al.^187^ | 2022 | Brazil | Case report | MECP2 duplication syndrome | 1 |  | 6 years | GMFM-88 A-E (%)  GMFM-88 Total (%) | To quantify motor changes after physiotherapy treatment based on the Bobath concept. | Treatment evaluation | NA (no citing) |  |
|  | Qian et al.^48^ | 2022 | China | Case report | Microdeletion of 4p16.2 | 1 |  | 1 year | GMFM-66 | To describe gross motor function. | Natural history and disease progression | NA (no citing) |  |
|  | Çankaya et al.^188^ | 2022 | Turkey | Cross-sectional study | Neurofibromatosis type 1 | 68 | 40 | 9.7 (3.8) years^c^ | GMFM-88 D-E (%) | To assess gross motor capacity. | Natural history and disease progression | NA (citing Russell 1998 study) |  |
|  | Ruck et al.^189^ | 2011 | Canada | Retrospective observational study | Osteogenesis Imperfecta (OI) | 60 | 43 | 3 years 11 months (2 years 3 months); 1 year 2 months–11 years 8 months | GMFM-88 A-E (%)  GMFM-88 Total (%) | To examine gross motor function of children with OI following initial Fassier– Duval rodding to the femur at 1 year, and to determine which factors are associated with change in gross motor function, ambulation, and functional performance. | Clinical characterization | Describing measurement properties other than CP (citing CP and Ruck-Gibis study)  “The  GMFM-88 showed good interrater reliability in children with OI and has been used in children with OI in Italy.” |  |
|  | Hoyer-Kuhn et al.^49^ | 2014 | Germany | Within-subjects study | OI | 53 |  | 9.1; 2.54–24.81 years | GMFM-66 | To evaluate the effect on mobility after 12 months. | Treatment evaluation | Describing measurement properties other than CP (citing Russell, Linder-Lucht and Ruck-Gibis study)  “Meanwhile the GMFM-88 has been validated for traumatic braininjury, Down syndrome and OI.” |  |
|  | Hoyer-Kuhn et al.^190^ | 2016 | Germany | Single-arm, open-label phase-2-trial study | OI | 10 | 9 | 7.0 (2.12); 5.0–11.0 years | GMFM-88 Total (%) | To investigate safety and efficacy of osteoclast inhibition with denosumab. | Treatment evaluation | NA (citing Ruck-Gibis J study) |  |
|  | Hoyer-Kuhn et al.^191^ | 2019 | Germany | Retrospective study | OI | 10 | 9 | 8.60 (1.83); 6.16–12.13 years | GMFM-88 Total (%) | To evaluate of an individualized biomarker-associated treatment regime with denosumab. | Treatment evaluation | NA (citing Ruck-Gibis study) |  |
|  | Reus et al.^192^ | 2013 | Netherlands | Two-year longitudinal single-blinded controlled trial (RCT) | PWS | 22 |  | Treatment: 14.2 (8.1) months  Control: 11.7 (6.3) months | GMFM-88 Total (%) | To investigate effects of growth hormone treatment on gross motor function. | Treatment evaluation | Describing validity in CP (citing CP study) |  |
|  | Reus et al^193^. | 2014 | Netherlands | Two-year randomized, single-blind controlled trial (RCT) | PWS | 22 |  | Treatment: 14.2 (8.1) months  Control: 11.7 (6.3) months | GMFM-88 Total (%) | To investigate relationships between muscle thickness, muscle strength, and motor performance. | Clinical characterization | NA (citing CP study |  |
|  | Fyfe et al.^194^ | 2007 | Australia | validation study | Rett syndrome | 97 |  | 2–19+ years | Selected 20 items from GMFM-88 for mobility assessment | To assess mobility skills in video-based evaluation tool. | Treatment evaluation | NA (citing CP study) |  |
|  | Kovela et al.^195^ | 2021 | India | Case report | Rubinstein-Taybi syndrome | 1 |  | 17 months | GMFM-88 Total (%) | To present gross motor function during the intervention. | Treatment evaluation | NA (citing CP study) |  |
|  | Balikci et al.^196^ | 2023 | Turkey | Case report | Rubinstein-Taybi Syndrome | 1 |  | 3 years | GMFM-88 A-E (raw & %)  GMFM-88 Total (raw & %) | To examine changes in Ayres Sensory Integration Intervention. | Treatment evaluation | Acknowledging lack of validation despite widespread use (citing the user’s manual)  “it is stated in the manual that it may be used with children with developmental delays or severe motor, so was deemed appropriate for this child.” |  |
|  | Goyal et al.^197^ | 2021 | India | Case report | Trisomy 10p along with terminal 14q deletion | 1 |  | 14 months | GMFM-88 A-B (%)  GMFM-88 Total (%) | To report on the progress of rehabilitation. | Treatment evaluation | Describing measurement properties other than CP (citing the user’s manual)  “GMFM-88 was used for the  evaluation of motor skills because its reliability and validity have been established for conditions other than  cerebral palsy” |  |
|  | Conklin et al.^198^ | 2021 | United States | Case report | Williams syndrome | 1 |  | 18 months | GMFM-88 D-E (%) | To highlight the functional outcome of an 8-week treadmill training program. | Treatment evaluation | Describing measurement properties other than CP. Acknowledging lack of validation despite widespread use (citing Russell 1998 study)  “The GMFM-88 is a reliable measure of gross motor functional mobility that has been validated for use in children with DS. The GMFM-88 has not been validated for use in children with WS. However, it was the most appropriate tool available to capture gross motor functional change over time, due to similarities to children with DS.” |  |
|  | Goyal et al.^199^ | 2020 | India | Case report | Xia-Gibbs Syndrome | 1 |  | 27 months | GMFM-88 Total (%) | To report on the progress of physiotherapy. | Treatment evaluation | Describing measurement properties other than CP (citing the user’s manual)  “GMFM-88 was used for evaluation as it is considered reliable and valid for use in conditions other than cerebral palsy” |  |
| Developmental Disorders | Vodakova et al.^200^ | 2022 | Czech Republic | Single-subject study | Autism Spectrum Disorders | 7 |  | 9.4 (2.0) years | GMFM-88 Total (%) | To evaluate the effect of a seven-week intervention program of the Halliwick method in the development of gross motor skills. | Treatment evaluation | NA (citing CP study) |  |
|  | Salem et al.^201^ | 2012 | United States | Single-blind, randomized controlled trial | Developmental delay | 40 |  | Experimental group: 49.3 (5.6) months  Control group: 48.0 (5.8) months | GMFM-88 D-E (%) | To provide information regarding gross motor function and assess changes as a result of the intervention. | Treatment evaluation | Describing measurement properties other than CP (citing the user’s manual)  “Evidence of the validity and reliability of the GMFM for use in children with motor disorders and children with disabilities has been established.” |  |
|  | Sant et al.^202^ | 2021 | India | Case report | Developmental delay | 1 |  | 7 months | GMFM-88 A-E (%)  GMFM-88 Total (%) | To evaluate the effect of early intervention on high-risk infants with delayed gross motor function along with longer Neonatal Intensive Care Unit stay. | Treatment evaluation | NA (citing CP study) |  |
|  | Lowe et al.^203^ | 2015 | United States | Randomized controlled trial | Developmental Delay | 21 | 20 | Treatment: 36.67; 26–51 months  Control: 39.50; 27–48 months | GMFM-88 D&E (raw) | To examine the effect of body weight supported treadmill training on gross motor skill development. | Treatment evaluation | Describing measurement properties other than CP (citing the user’s manual)  “The GMFM has established reliability and validity in pediatric populations, specifically children with cerebral palsy and Down Syndrome.” |  |
|  | Ha et al.^204^ | 2021 | Korea | Case report | Developmental delay | 1 |  | 11 months | GMFM-88 Total (%) | To investigate the effect of the Vojta approach. | Treatment evaluation | Describing validity in CP (citing CP study) |  |
|  | Lee et al.^78^ | 2017 | South Korea | Within-subjects study | Developmental Delay without CP  (Subtypes)  Arnold–Chiari malformation  Dandy–Walker syndrome  Partial agenesis of corpus callosum  Microcephaly  CHARGE syndrome  Prader Willi syndrome  Mitochondrial disease  Unspecified | 18 |  | 48.73 (18.47) months | GMFM | To evaluate the effectiveness of intensive neurodevelopmental treatment on gross motor function for the children having developmental delay, with or without CP. | Treatment evaluation | Describing validity in CP (citing CP study) |  |
|  | Winchester et al.^205^ | 2002 | United States | A repeated-measures within-participants design | Developmentally delayed children  (Subtypes)  Spina bifida  Down Syndrome  Down Syndrome & Autism  Traumatic Brain Injury | 7 | 5 | 57.8, 76.3, 86.5, 86.5 and 90.5 months | GMFM-88 C-E (raw & %) | To evaluate the effects of a therapeutic riding program on gross motor function. | Treatment evaluation | Describing measurement properties other than CP (citing Russell 1998 study and citing non-measurement study other than CP)  This test has been validated on  children ages 2 to 12 to detect changes in gross motor function in children with developmental delays secondary to diagnoses, such as cerebral palsy, brain injury and Down syndrome. |  |
|  | Ha et al.^206^ | 2022 | Korea | Randomized controlled trial | Hypotonia who were diagnosed with developmental delay | 20 |  | Experimental group: 45.00 (18.95) months  Control group: 51.70 (27.42) months | GMFM-88 B (%)  GMFM-88 Total (%) | To determine the effect of Vojta therapy. | Treatment evaluation | Describing validity in CP (citing CP study) |  |
|  | Hielkema et al.^27^ | 2013 | Netherlands | Prospective study | Infants at very high risk for CP | 12 | 6 | Median 3.5; 1.5–7 months | GMFM-88  GMFM-66  GMFM adapted version | To evaluate longitudinal applicability of the GMFM in infants younger than 2 years. | Clinical characterization | Describing validity in CP (citing CP study) |  |
|  | Arndt et al.^68^ | 2008 | United States | Randomized controlled trial | Infants with posture and movement dysfunction | 10 |  | Intervention group: 8.95 (2.93); 5.25 - 12.0 months  Control group: 8.9 (3.59); 4.0 - 11.75 months | GMFM-88 | To evaluate the efficacy of a NDT-based sequenced trunk co-activation protocol. | Treatment evaluation | Describing validity in CP (citing CP study) |  |
|  | Schlichting et al.^207^ | 2022 | Brazil | Pre-post Feasibility Experimental Study | Infat at risk for delayed neuromotor development | 10 |  | Median 27.5 weeks; 5–61 weeks | GMFM-88 A-C (%)  GMFM-88 Total (%) | To verify the effects of a telerehabilitation program on gross motor function. | Treatment evaluation | Describing validity in CP (citing CP study) |  |
|  | Ko et al.^208^ | 2014 | Korea | Cross-sectional study | Mental retardation | 9 |  | 53.8 (17.9) months | GMFM-88 A-E (%)  GMFM-88 Total (%) | To compare gross motor functions between CP and mental retardation subjects. | Clinical characterization | Describing validity in CP (citing CP study) |  |
|  | Kolobe et al.^209^ | 1998 | United States | Prospective study | Motor delay | 18 |  | Adjusted age 12.9 (6.2); 4.2–22.0 months | GMFM-88 Total (%) | To compare the GMFM and the Peabody Developmental Gross Motor Scale as measures of change. | Clinical characterization | Describing measurement properties other than CP (citing the user’s manual)  “The GMFM was validated on 136 children with CP, 25 children with acute head injury, and 34 children without motor delays who ranged in age from 1 month to 4.3 years.” |  |
|  | An et al.^210^ | 2022 | United States | Longitudinal, prospective cohort study | Motor delays | 92 | 56 | 10.23 (2.63); 7–16 months | GMFM-88 B (raw) | Examined whether the emergence of object permanence in infants is associated with sitting development. | Clinical characterization | Describing measurement properties other than CP (citing Russell 1998 and Linder-Lucht M study)  “it has been validated for use with children with developmental delays secondary to diagnoses such as Down syndrome and brain injury.” |  |
|  | Elbasan et al.^211^ | 2011 | Turkey | Cross-sectional study | Motor mental retardation | 35 |  | 12.23 (3.8); 5–17 years | GMFM-88 Total (%) | To examine the impact of visual perception and motor functions on the activities of daily living. | Clinical characterization | Describing measurement properties other than CP (citing CP and Russell study)  “It was reported to be a valid, reliable and sensitive method as much as video-tape recordings for demonstrating the changes in motor functions, i.e. the effectiveness of treatment, in children with cerebral palsy and other disabilities” |  |
|  | onvicine et al.^212^ | 2005 | (Brazil) | Retrospective comparative study | Pre-term infant | 5 |  | 2 and 4 months assessment points | GMFM-88 (6 items focused on head control) | To compare head control acquisition between preterm and full-term infants. | Treatment evaluation | NA (no citing) |  |
|  | Indravadan et al.^79^ | 2017 | India | Within-subjects study | Premature infant | 30 |  | NA | GMFM | To assess the effect of early physical therapy treatment. | Treatment evaluation | NA (no citing) |  |
|  | Kepenek et al.^213^ | 2016 | Turkey | Prospective single-blinded study | Preterm infant | 22 |  | Outcome measured corrected 12 months of age | GMFM-88 A-D (%) | To compare the results with neurologic examination findings. | Treatment evaluation | NA (citing Russell study) |  |
|  | Mann et al.^80^ | 2012 | India | Randomized controlled trial | Preterm infants | 30 |  | 33.25 weeks | GMFM | To evaluate the efficacy of early developmental intervention programme over the home intervention programme for gross motor function. | Treatment evaluation | NA (no citing) |  |
| Neoplastic Diseases and Post-treatment Complications | Wright et al.^214^ | 1998 | Canada | Cross-sectional study | Acute lymphoblastic leukemia (ALL) | 36 |  | Median 9.3; 5.5–14.5 years | GMFM-88 D-E (%) | To determine the long-term effects of cancer treatment in childhood on gross motor skills. | Treatment evaluation | NA (no citing) |  |
|  | Wiernikowski et al.^69^ | 2005 | Canada | Prospective Observational Study | ALL  Non-Hodgkin’s lymphoma | 10 |  | Median 7.2; 3.6–14.6 years | GMFM-88 D-E | To assess the impact of alendronate on gross motor function. | Treatment evaluation | NA (no citing) |  |
|  | Gohar et al.^215^ | 2011 | United States | Within-subjects study | ALL | 9 |  | Median 4.0; 2–14 years | GMFM-88 Total (%) | To examine the feasibility of an in-hospital physical therapy program and home exercise program during the first four phases of medical treatments. | Treatment evaluation | NA (citing CP study and Wright study) |  |
| Metabolic/Degenerative Disorders | Diaz et al.^70^ | 2021 | United States | Phase 1/2 open-label study | Arginase 1 deficiency | 16 | 14 | Total: 15 (8.5); 5–31 years | GMFM-88 D-E | To evaluate safety, pharmacokinetics, and pharmacodynamic activity of repeat dosing with pegzilarginase. | Treatment evaluation | NA (no citing) |  |
|  | Gavazzi et al.^50^ | 2022 | United States | Prospective natural history study | Beta-propeller protein-associated neurodegeneration | 27 | 11 | 1.53–24.61 years | GMFM-66-IS | To inform clinical care. | Treatment evaluation | NA (citing CP study) |  |
|  | Assadi et al.^81^ | 2010 | United States | Prospective study | Canavan disease | 6 |  | 9.5 months | GMFM | To test the efficacy of treatment with lithium. | Treatment evaluation | NA (no citing) |  |
|  | Schaefers et al.^51^ | 2021 | Netherlands | Case Series | Ceroid lipofuscinosis type 2 (CLN2) | 2 |  | 47 and 23 months | GMFM-66 | To report gross motor function progress in two siblings with classic late-infantile CLN2 disease treated with cerliponase alfa for 2 years. | Treatment evaluation | NA (no citing) |  |
|  | Sessa et al.^216^ | 2016 | Italy | Non-randomized, open-label, phase 1/2 trial | Early-onset metachromatic leukodystrophy | 9 |  | 7–59 months | GMFM-88 Total (%) | To assess efficacy of haemopoietic stem-cell gene therapy in gross motor function. | Treatment evaluation | NA (no citing) |  |
|  | Paschall et al.^217^ | 2021 | United States | Cross-sectional Study | Glycogen storage disease (GSD) type III | 22 | 16 | Median 11.52; 3.7–17.7 years | GMFM-88 A-E (%)  GMFM-88 Total (%) | To investigate the early musculoskeletal findings in children with GSD IIIa, compared to a cohort of adults with GSD IIIa. | Natural history and disease progression | NA (no citing) |  |
|  | Martinelli et al.^218^ | 2012 | Italy | Single-site, open-label phase 2A study (within-subjects study) | Leigh syndrome | 10 |  | 6.3; 1–13 years | GMFM-88 Total (%) | To report on gross motor function of children treated with EPI-743. | Treatment evaluation | Describing measurement properties other than CP (citing CP study)  “The GMFM has been validated as an outcome measurement instrument in intervention trials of children with neuromuscular disorders.” |  |
|  | Tucker^219^ | 2022 | United States | Case report | Long-Chain 3-Hydroxyacyl-CoA Dehydrogenase Deficiency | 1 |  | 15 years | GMFM-88 Total (%) | To describe effectiveness of Robotic-Assisted Gait Training and Aquatic Physical Therapy on gross motor function. | Treatment evaluation | NA (no citing) |  |
|  | Dali et al.^220^ | 2010 | Denmark | Open-label phase I single-center clinical trial study | Metachromatic leukodystrophy (MLD) | 13 |  | Median 34; 25–59 months | GMFM-88 Total (raw) | To examine using multislice spectroscopic imaging and the findings compared with clinical status. | Treatment evaluation | Describing validity in CP (citing CP study) |  |
|  | Dali et al.^221^ | 2015 | Denmark | Open-label, nonrandomized, uncontrolled, Phase I clinical trial | MLD | 13 |  | 37.5 months | GMFM-88 Total (raw) | To present the Patient's gross motor function. | Treatment evaluation | Describing measurement properties other than CP (citing CP study)  “This measures a child’s best abilities across several gross motor domains and is validated to describe gross motor skills and changing abilities over time in children with neurological disorders.” |  |
|  | Beschle et al.^222^ | 2020 | Germany | Prospective study | MLD | 12 |  | 11.6; 5–18.2 years | GMFM-88 Total (%) | To analyze the clinical follow-up 2 years after hematopoietic stem cell transplantation. | Treatment evaluation | Describing validity in CP (citing CP study) |  |
|  | Dali et al.^223^ | 2020 | Denmark | Multicenter, open-label, dose-escalation study | MLD | 24 |  | Cohort 1: 31.5 (11.50); 22.0–54.0 months  Cohort 2: 47.3 (20.11); 30.0–80.0 months  Cohort 3: 52.2 (31.17); 23.0–107.0 months  Cohort 4: 48.5 (24.22); 19.0–81.0 months | GMFM-88 Total (%) | To examine the safety and tolerability of intrathecally delivered recombinant human arylsulfatase. | Treatment evaluation | NA (no citing) |  |
|  | Dali et al.^224^ | 2021 | Denmark | Open-label, nonrandomized, dose-escalation trial | MLD | 13 |  | 37.5 months | GMFM-88 Total (%) | To evaluate the safety and efficacy of intravenous recombinant human arylsulfatase A. | Treatment evaluation | Describing measurement properties other than CP (citing CP and Russell study)  “This measures a child’s best abilities across several  gross motor domains and is validated to describe gross  motor skills and changing abilities over time in children  with neurological disorders.” |  |
|  | Fumagalli et al.^52^ | 2022 | Italy | Prospective, non-randomized, phase 1/2 clinical study | MLD | 60 |  | Intention-to-treat set  - Late infantile: 12.81 (4.3) months  - Early juvenile: 64.86 (33.4) months  Natural history cohort  - Late infantile: 20.64 (4.7) months  - Early juvenile: 51.98 (19.2) months | GMFM-88 Total (%) | To investigate the safety and efficacy of atidarsagene autotemcel (arsa-cel). | Treatment evaluation | NA (no citing) |  |
|  | Oswald et al.^52^ | 2023 | Australia | N-of-1 trial (single Case Study) | Mitochondrial Phenylalanyl-tRNa-Synthetase Deficiency  (Mitochondrial FARS2 Deficiency) | 1 |  | 3 years | GMFM-66 | To measure effects of oral phenylalanine on gross motor function for a child with FARS2 deficiency. | Treatment evaluation | NA (no citing) |  |
|  | Groeneweg et al.^226^ | 2020 | Netherlands | Retrospective study | Monocarboxylate transporter 8 deficiency | 151 | 86 | Median 4.8 (0.44–66.8) years | GMFM-88 Total (%) | To systematically assess the phenotypic characteristics and natural history of patients. | Natural history and disease progression | NA (citing Russell study) |  |
|  | Narita et al.^227^ | 2016 | Japan | Multicenter, open-label pilot study (within-subjects study) | neuronopathic Gaucher disease | 5 |  | 28, 20, 15, 3, 25 years | GMFM-88 A-E (%)  GMFM-88 Total (%) | To assess effects of ambroxol treatment on gross motor function. | Treatment evaluation | NA (no citing) |  |
|  | Fujii et al.^228^ | 2014 | Japan | Semi-quantitative clinical evaluation study | Oxidative phosphorylation disorders | 4 | 3 | 8 years 4 months, 8 months and 1 year 8 months | GMFM-88 A-B (%)  GMFM-88 Total (%) | To evaluate the efficacy of pyruvate therapy. | Treatment evaluation | NA (citing CP study) |  |
|  | Sarret et al.^71^ | 2018 | France | Cross-sectional study | Pelizaeus-Merzbacher disease | 35 |  | Median 8.6; 2.3–43.5 years | GMFM-88 | To compare gross motor function among three groups, and correlate with diffusion tensor imaging parameters. | Natural history and disease progression | NA (citing Russell study) |  |
|  | Brandalize et al.^72^ | 2004 | Brazil | Program evaluation study | Phenylketonuria (PKU)  Persistent hyperphenylalaninemia | 32 |  | 0–6 years | GMFM-88 | To evaluate motor development in early-treated PKU patients. | Treatment evaluation | Describing validity in CP (citing CP study) |  |
|  | Verhoog et al.^229^ | 2008 | Netherlands | Cross-sectional study | Sjögren–Larsson syndrome | 17 | 11 | GMFM: 1 year 11months–15 years 2 months | GMFM-88 A-E (%)  GMFM-88 Total (%) | To describe motor capacity. | Treatment evaluation | NA (citing Russell study) |  |
| Other Conditions | Church et al.^230^ | 2020 | United States | Retrospective review study | Idiopathic clubfoot  Arthrogryposis with associated clubfoot | 117 |  | 4.8 (0.8) years | GMFM-88 D (raw) | To compare the effectiveness of the Ponseti method over a 5-year span. | Treatment evaluation | NA (no citing) |  |
|  | Shin et al.^231^ | 2020 | South Korea | Retrospective Observational study | Infants with tracheostomy  Infants without tracheostomy | 165 |  | Tracheostomy: 12.6 (6.3) months  No tracheostomy: 10.3 (12.7) months | GMFM-88 Total (%) | To investigate how tracheostomy affects early motor development. | Treatment evaluation | NA (citing CP study) |  |
|  | Torpey et al.^232^ | 2000 | NA | Case report | Sequelae of septic hip | 1 |  | 42 months | GMFM-88 B (raw & %) | To investigate the short-term effectiveness of the S.W.A.S.H. orthosis. | Treatment evaluation | Describing measurement properties other than CP (citing Russell and the user's manual)  “it also was validated on children with acute head injury and children without motor delay.” |  |
| Abbreviations: ABI, acquired brain injury; AGS, Aicardi Goutières syndrome; AHC, alternating hemiplegia of childhood; ALL, acute lymphoblastic leukemia; AS, Angelman syndrome; AT, ataxia telangiectasia; CDKL5, Cyclin-Dependent Kinase-Like 5; CHARGE, CHARGE syndrome; CLN2, ceroid lipofuscinosis type 2; COPM, Canadian Occupational Performance Measure; COVID, Coronavirus Disease; CP, cerebral palsy; CZS, congenital Zika syndrome; DMD, Duchenne muscular dystrophy; DS, Down syndrome; FARS2, Phenylalanyl-tRNA Synthetase 2; FCMD, Fukuyama congenital muscular dystrophy; GAN, giant axonal neuropathy; GMFM, Gross Motor Function Measure; GSD, glycogen storage disease; HIV, human immunodeficiency virus; HNRNPH2, Heterogeneous Nuclear Ribonucleoprotein H2; HSP, hereditary spastic paraparesis; ICC, Intraclass Correlation Coefficient; IOPD, infantile-onset Pompe disease; IQR, Interquartile Range; MECP2, Methyl CpG Binding Protein 2; MLD, metachromatic leukodystrophy; NA, not available; NDT, neurodevelopmental treatment; NMDA, N-methyl-d-aspartate; OBPP, obstetric brachial plexus palsy; OI, osteogenesis imperfecta; PD, Pompe disease; PT, Physical Therapy; PWS, Prader-Willi syndrome; RAGT, robot assisted gait training; RCT, Randomized Controlled Trial; SD, standard deviation; SDR, Selective Dorsal Rhizotomy; SMA, spinal muscular atrophy; TBI, traumatic brain injury; TBCD, Tubulin Beta Class D; VRTT, virtual reality plus treadmill training; ZIKV, Zika virus.  ^a^ For non-English speaking countries, a country name in parentheses indicates that the paper was written in that country's native language  ^b^ Sample sizes are shown when GMFM data were available  ^c^ Age data presented for GMFM sample | | | | | | | | | | | | | |
